# Analysis of allelic variants of *RhMLO* genes in rose and functional studies on susceptibility to powdery mildew related to clade V homologs

**DOI:** 10.1007/s00122-021-03838-7

**Published:** 2021-05-02

**Authors:** Peihong Fang, Paul Arens, Xintong Liu, Xin Zhang, Deepika Lakwani, Fabrice Foucher, Jérémy Clotault, Juliane Geike, Helgard Kaufmann, Thomas Debener, Yuling Bai, Zhao Zhang, Marinus J. M. Smulders

**Affiliations:** 1 Plant Breeding, Wageningen University and Research, 6708 PB Wageningen, The Netherlands; 2 Beijing Key Laboratory of Development and Quality Control of Ornamental Crops, Department of Ornamental Horticulture, College of Horticulture, China Agricultural University, Beijing, 100193 China; 3 IRHS, Agrocampus-Ouest, INRAE, Université D’Angers, SFR 4207 QuaSaV, 49071 Beaucouzé, France; 4 Institute of Plant Genetics, Molecular Plant Breeding Unit, Leibniz Universität Hannover, Hannover, Germany

## Abstract

**Key message:**

Rose has 19 MLO genes. Of these, RhMLO1 and RhMLO2 were shown to be required for powdery mildew infection, which suggests their potential as susceptibility targets towards disease resistance.

**Abstract:**

Powdery mildew, caused by *Podosphaera pannosa*, is one of the most serious and widespread fungal diseases for roses, especially in greenhouse-grown cut roses. It has been shown that certain *MLO* genes are involved in powdery mildew susceptibility and that loss of function in these genes in various crops leads to broad-spectrum, long-lasting resistance against this fungal disease. For this reason, these *MLO* genes are called susceptibility genes. We carried out a genome-wide identification of the *MLO* gene family in the *Rosa chinensis* genome, and screened for allelic variants among 22 accessions from seven different *Rosa* species using re-sequencing and transcriptome data. We identified 19 *MLO* genes in rose, of which four are candidate genes for functional homologs in clade V, which is the clade containing all dicot *MLO* susceptibility genes. We detected a total of 198 different allelic variants in the set of *Rosa* species and accessions, corresponding to 5–15 different alleles for each of the genes. Some diploid *Rosa* species shared alleles with tetraploid rose cultivars, consistent with the notion that diploid species have contributed to the formation of tetraploid roses. Among the four *RhMLO* genes in clade V, we demonstrated using expression study, virus-induced gene silencing as well as transient RNAi silencing that two of them, *RhMLO1* and *RhMLO2*, are required for infection by *P. pannosa* and suggest their potential as susceptibility targets for powdery mildew resistance breeding in rose.

**Supplementary Information:**

The online version contains supplementary material available at 10.1007/s00122-021-03838-7.

## Introduction

Rose is one of the most important ornamental plants worldwide, holding great economic and cultural value. It was domesticated both in China and in Europe several thousand years ago for its application in gardens, use in medical treatment and usage for fragrance (Debener and Byrne 2014). Today, roses are widespread and are sold as the most popular garden plants or cut flowers with various colours, growth types and long-lasting flowering period. One of the reasons for this wide variation is the crossing of different rose species by a large number of breeders for a long period of time. Because of this activity, wild species with varying ploidy levels have contributed to the genome of tetraploid cut and garden roses (Koopman et al. 2008; Dubois and Sakr 2012; Zhang et al. 2013; Vukosavljev et al. 2013; Smulders et al. 2019).

Powdery mildew causes significant economic losses of agricultural crops as well as ornamental plants. In rose it is caused by *Podosphaera pannosa*. It was estimated that up to 40% of the pesticides applied on rose are used to control this disease (Debener and Byrne 2014). In order to reduce the use of pesticides, an alternative to overcome this problem can be an introgression program focusing on dominant plant resistance genes (*R*-genes); however, it can be difficult to transfer these genes into cultivated species due to interspecific crossability barriers (Fu et al. 2009). Furthermore, *R*-gene-mediated race-specific resistance may be easily overcome by new races of the pathogen in a short period (Pavan et al. 2010).

Recently, impairing plant susceptibility genes (*S* genes), which are plant genes contributing to susceptibility to pathogens, has been proposed as a novel breeding strategy (Pavan et al. 2010). The *MLO* (*Mildew resistance Locus O*) gene is a remarkable example of an *S* gene. It was first observed in barley that mutations in a particular *MLO* gene confer resistance to *Blumeria graminis f. sp. Hordei* (Jørgensen 1992; Piffanellii et al. 2004). As a typical plant *S* gene, the presence of the barley *MLO* gene is required for the powdery mildew infection. When the function of barley *MLO* gene is lost, local callose deposition occurs in the epidermal cells, causing the cell wall to thicken, so that the fungus fails to penetrate the epidermal cells of the plant to form a haustorium and obtain nutrients (Wolter et al. 1993). As a consequence, the plant acquires resistance to powdery mildew (Consonni et al. 2006; Eichmann and Huckelhoven 2008; Elliott et al. 2002; Kusch and Panstruga 2017). Loss of barley *MLO* gene function produced resistance to all known physiological races of barley powdery mildew, and *mlo* has been used in barley breeding since 1979 without being overcome by the pathogen (Büschges et al. 1997; Jørgensen 1992; Lyngkjaer et al. 2000). Following barley, natural *mlo* mutants have been identified in tomato, pea, melon and cucumber (Bai et al. 2008; Berg et al. 2015; Humphry et al. 2011; Nie et al. 2015; Cheng et al. 2015; Pavan et al. 2013). The pea *mlo* mutant (*er1*) has been worldwide used in breeding for powdery mildew resistance since it was first reported in 1948 (Harland 1948). These examples show that *mlo*-based resistance can be identified in various plant species and is effective to a broad-spectrum powdery mildew species. In a number of plant species, a reduced susceptibility to powdery mildew was generated by the knockdown of particular *MLO* genes, i.e. in apple and grape by RNAi (Pessina et al. 2016a, b) and in pepper by virus-induced gene silencing (VIGS) (Zheng et al. 2013).

The susceptibility *MLO* gene family encodes proteins that belong to a plant-specific membrane protein family containing seven-transmembrane helices (Devoto et al. 2003; Kim et al. 2002; Panstruga, 2005). Up to 20 *MLO* homologs have been identified in several plant species. Phylogenetically, *MLO* isoforms experimentally shown to be functional as a *S* gene for powdery mildew are grouped together in one clade, clade IV for monocots and clade V for dicots (Zheng et al. 2016). In rose, only four *MLO* genes isolated from *Rosa multiflora* have been reported (Kaufmann et al. 2012), two of which (*RhMLO1* and *RhMLO2*) were up-regulated in the rose-powdery mildew pathogen interaction (Qiu et al. 2015a). An antisense *RhMLO1* transgenic rose line showed postponed conidia development upon powdery mildew infection, but as the fragment used for RNAi covered a region that is also present in *RhMLO2*, specificity to *RhMLO1* silencing remained unclear (Qiu et al. 2015b).

At present, next-generation sequencing is extensively used to accelerate genetic, genomic, transcriptomic and epigenetic analysis. It becomes convenient and cheap to generate sequences from a large variety of samples. A reference genome and resequenced genomes of several related species or accessions enable identification of candidate genes and alleles prior to validation of the functional allele (Smulders et al. 2019, 2020). This has been recently done for *MLO* genes in various species, including soybean (Deshmukh et al. 2014), *Cicer arietinum* and *Medicago truncatula* (Deshmukh et al. 2017), *Populus trichocarpa* (Filiz and Vatansever 2018), pumpkin (Win et al. 2018), lentil (Polanco et al. 2018), bitter gourd (Chen et al. 2021), for the promotor region of *MLO* genes in melon and other species (Andolfo et al. 2019), and for other susceptibility genes (Pirrello et al. 2021). In rose overviews have been generated for *WRKY* genes (Liu et al. 2019), NBS-LRR genes (Lopez Arias et al. 2020), the disease resistance gene family *Rdr1* (Menz et al. 2020), AP2/ERF transcription factors (Li et al. 2021), and S-RNases and F-box genes involved in self-incompatibility (Vieira et al. 2021). These inventories may be used as a basis to find or generate loss-of-function alleles of relevant MLO genes.

In this study, we identified putative *RhMLO* orthologs in the *R. chinensis* cv. ‘Old Blush’ (OB) genome sequence. In order to analyse the allelic variation of these *RhMLO* genes, we also explored resequencing data of a set of seven wild rose species (Hibrand Saint-Oyant et al. 2018) and transcriptome data of cut and garden roses (Dubois and Sakr 2012; Koning-Boucoiran et al. 2015). In addition, we used two different approaches for a functional analysis of the four clade V *MLO* homologs in rose: a transient knockdown approach through VIGS, followed by inoculation with powdery mildew as well as a transient RNAi silencing in petals and subsequent mildew inoculation. These two approaches used different strains of powdery mildew and different rose varieties. We show that, in addition to *RhMLO1, RhMLO2* also acts as a susceptibility factor to powdery mildew, while *RhMLO3* and *RhMLO4* seem not to be involved or only with a minor effect.

## Materials and methods

### Identification of Rosa chinensis ‘Old Blush’ homologs of MLO genes

We refer here to all *MLO* genes from the genus *Rosa* as *RhMLO* genes, following the naming convention set by Kaufmann et al. (2012), but we add a reference to the *Rosa* species in which they were found. The protein sequences of four *RhMLOs* from *Rosa multiflora* (*RhMLO1-4* in Kaufmann et al. 2012; GenBank accession numbers JX847131-JX847134) and all *MLO* genes used by Pessina et al. (2014), namely 18 *FvMLOs* from *Fragaria vesca*, 19 *PpMLOs* from *Prunus persica*, 21 *MdMLOs* from *Malus domestica* as well as 15 *AtMLOs* from *A. thaliana* http://www.arabidopsis.org/, were used for the identification of *MLO* gene members in the rose reference genome sequence of *Rosa chinensis*. All protein sequences of *Rosa chinensis* ‘Old Blush’ (Hibrand Saint-Oyant et al. 2018) were downloaded from GDR (Genome Database for Rosaceae, Jung et al. (2019); https://www.rosaceae.org/). Using BLASTp the best hits were identified against this set of *MLO* genes from different plant species. We used an e-value of 1e^−5^ as minimum, and report all e-values in Supplementary Excel File S1.

Based on the recently released high-quality *F. vesca* genome v4.0.a1 (Edger et al. 2018) and the updated gene annotation v4.0.a2 (Li et al. 2019), we replaced *FvMLO11* from v1.0 (Shulaev et al. 2010) by *FvH4_1g11630.1* because the protein sequence of *FvMLO11* hits two different sequences named *FvH4_1g11630.1* and *FvH4_1g11620.1* with 100% (545 aa alignment) and 97.8% (413 aa alignment) identity, respectively, in v4.0.a1. *FvH4_1g11630.1* has a gene structure consisting of 14 exons, which code for a protein with seven transmembrane domains, corresponding to the other characterized *MLO* genes (which have 12–15 exons), while *FvH4_1g11620.1* only has 11 exons and the predicted protein lacks a typical transmembrane domain. *FvH4_1g11620.1* may therefore represent an assembly error. The position and number of transmembrane domains were predicted using HMMER (https://www.ebi.ac.uk/Tools/hmmer/).

#### Bioinformatic analysis of MLO gene family in rose

The chromosomal location and predicted intron/exon structure of each *RhMLO* gene were extracted from the available genomic information of *Rosa chinensis* genome HapOB2 v1.0 in GDR. We visualized the chromosomal location and intron/exon structure by MapChart 2.32 and online tool GSDS 2.0 (http://gsds.cbi.pku.edu.cn/), respectively.

A phylogenetic analysis was performed for the *RhMLO* proteins obtained from the BLAST search. For this, next to the *MLO* sequences obtained from rose, apple, strawberry, peach and Arabidopsis, we also included a series of *MLO* homologs that have been functionally associated with powdery mildew susceptibility: *HvMLO* (Z83834), *OsMLO2* (AF384030), *TaMLO1_A1b* (AX063298), *TaMLO_B1* (AF361932), *SlMLO1* (AAX77013), *CaMLO2* (AFH68055), *PsMLO1* (FJ463618), *MtMLO1* (HQ446457), *LjMLO1* (AAX77015), VvMLO6 (Genoscope ID: GSVIVT00018217001; http://www.genoscope.cns.fr/externe/GenomeBrowser/Vitis/entry_ggb.html), *VvMLO7* (Genoscope ID: GSVIVT00018219001), *CsMLO1* (Cucurbit Genomics Database ID: Csa5M623470.1; http://cucurbitgenomics.org/organism/2) and *NtMLO1* (AIT98396) (Buschges et al. 1997; Elliott et al. 2002; Bai et al. 2008; Feechan et al. 2008; Winterhagen et al. 2008; Humphry et al. 2011; Zheng et al. 2013; Berg et al. 2015; Nie et al. 2015; Pessina et al. 2016b).

*MLO* protein sequences were aligned first with Clustalx 1.83 and then further aligned by Clustalw in MEGA7. Sequences were trimmed at the C and N terminal parts. Different amino acid substitution models were tested, and a Poisson model with uniform rates was chosen as most suitable. Then, the alignment was used to generate phylogenetic trees in MEGA7 using ML (maximum likelihood) and UPGMA (unweighted pair group method with arithmetic mean) methods with a bootstrap value of 1000.

#### Expression pattern of RhMLO genes

To determine the expression pattern of *RhMLO* genes identified, their full-length protein sequences were used in a tBLASTn analysis of the *ROSAseq* transcriptome database https://lipm-browsers.toulouse.inra.fr/plants/R.chinensis (Dubois and Sakr 2012) with the parameters: e-value 1e-10, identity 90% or more. As next-generation sequencing often yields sequences to only parts of the gene, more than one cluster of target sequences in transcriptome database could match each *RhMLO*, in such case we used the hit with the highest score.

#### Identification of alleles of RhMLO genes

Allelic variation across candidate *RhMLO* genes was analysed in assembled contigs of twenty-two resequenced *Rosa* accessions of the diploid rose species *R. persica, R. moschata, R. xanthina spontanea, R. chinensis var. spontanea, R. laevigata*, *R. minutifolia alba* and *R. rugosa* (Hibrand Saint-Oyant et al. 2018; assemblies unpublished). In addition, transcriptome data from earlier studies (Dubois and Sakr 2012; Koning-Boucoiran et al. 2015) were also used. The origin of all sequence information used is listed in Table 1.

**Table 1:** The 22 accessions of *Rosa* species and varieties used in this study

Accession code (no.)	Rosa species, variety or accession	Ploidy	Data type	Group	Origin of species/ Breeder of cultivar	References
1	*R.chinensis cv.‘Old Blush’*	2 ×	WGS/Transcriptome	Wild rose	China	(Hibrand Saint-Oyant et al. 2018; Dubois and Sakr 2012)
2	*R. chinensis var. spontanea*	2 ×	Re-sequencing	Wild rose	China	(Hibrand Saint-Oyant et al. 2018)
3	*R. laevigata*	2 ×	Re-sequencing	Wild rose	China-Taiwan	(Hibrand Saint-Oyant et al. 2018)
4	*R. minutifolia*	2 ×	Re-sequencing	Wild rose	North America	(Hibrand Saint-Oyant et al. 2018)
5	*R. moschata*	2 ×	Re-sequencing	Wild rose	Asia Minor	(Hibrand Saint-Oyant et al. 2018)
6	*R. persica*	2 ×	Re-sequencing	Wild rose	Central Asia	(Hibrand Saint-Oyant et al. 2018)
7	*R. rugosa*	2 ×	Re-sequencing	Wild rose	Northern China-Japan-Korea	(Hibrand Saint-Oyant et al. 2018)
8	*R. xanthina spontanea*	2 ×	Re-sequencing	Wild rose	Asia	(Hibrand Saint-Oyant et al. 2018)
9	P540	4 ×	Transcriptome	Cut rose	Terra Nigra	(Koning-Boucoiran et al. 2015)
10	P867	4 ×	Transcriptome	Cut rose	Terra Nigra	(Koning-Boucoiran et al. 2015)
11	Adelaide Hoodless (AH)	4 ×	Transcriptome	Garden rose	Marshall	(Koning-Boucoiran et al. 2015)
12	Diamond Border (DB)	4 ×	Transcriptome	Garden rose	Olesen	(Koning-Boucoiran et al. 2015)
13	Graham Thomas (GT)	4 ×	Transcriptome	Garden rose	Austin	(Koning-Boucoiran et al. 2015)
14	Heritage (He)	4 ×	Transcriptome	Garden rose	Austin	(Koning-Boucoiran et al. 2015)
15	J.P. Connell (JPC)	4 ×	Transcriptome	Garden rose	Svejda	(Koning-Boucoiran et al. 2015)
16	Morden Blush (MB)	4 ×	Transcriptome	Garden rose	Marshall	(Koning-Boucoiran et al. 2015)
17	Morden Centennial (MC)	4 ×	Transcriptome	Garden rose	Marshall	(Koning-Boucoiran et al. 2015)
18	Morden Fireglow (MF)	4 ×	Transcriptome	Garden rose	Marshall	(Koning-Boucoiran et al. 2015)
19	Nipper (Ni)	4 ×	Transcriptome	Garden rose	Harkness	(Koning-Boucoiran et al. 2015)
20	Prairie Joy (PJ)	4 ×	Transcriptome	Garden rose	Marshall	(Koning-Boucoiran et al. 2015)
21	Red New Dawn (RND)	4 ×	Transcriptome	Garden rose	Robichon	(Koning-Boucoiran et al. 2015)
22	*Rosa multiflora* Rh88	2 ×	Transcriptome	Garden rose		(Koning-Boucoiran et al. 2015)

For the seven resequenced rose species (accessions No. 2–8 in Table 1), we used the predicted protein sequences. Sequences with an amino acid identity higher than 75% in a BLASTp to the 19 *RhMLOs* in the *R. chinensis* genome sequence were selected and checked manually, because in some cases two or more partial sequences from a single accession matched the query sequence, which may represent different parts of the same *MLO* gene. In such cases, we named these short sequences-a, -b and -c. We have tentatively assumed they were from the same allele, unless there was evidence for two alleles in that accession. Subsequently, the corresponding DNA sequences were retrieved from the resequencing database.

For diploid and tetraploid accessions No. 9 to 22 (Table 1) only RNA-seq data were available. The 19 *RhMLO* genes from *R. chinensis cv.* ‘Old Blush’ identified were used (without introns) in a tBLASTn analysis with a cut-off at 90% identity, and the longest sequences were kept.

All DNA sequences were aligned using Seqman Pro and MegAlign (DNASTAR) and alignments were corrected manually. Alleles of the same gene were distinguished from different genes manually, based on SNPs in the coding region. Criteria used to assign alleles to a single gene: alleles from the same gene should be > 90% and preferably > 95% similar, different genes have a different location in the assembled genome, maximum two alleles per locus/gene in a diploid, four in a tetraploid genotype. This produced a non-redundant list of candidate genes. It was not necessary to assume that any of the *Rosa* species had more than 19 genes.

### Structural analysis of RhMLO1-RhMLO19

A multiple sequence alignment was made to highlight transmembrane regions and conserved domains using Clustalx 1.83 software. The online tool HMMER (https://www.ebi.ac.uk/Tools/hmmer/) was used to identify transmembrane domains. The gene structures of *RhMLO1*-*RhMLO19* were analysed through the online Gene Structure Display Server (http://gsds.cbi.pku.edu.cn/).

### Functional analyses of clade V RhMLO genes using VIGS

To test whether the four *MLO* genes (*RhMLO1-4*) from clade V act as susceptibility gene for powdery mildew, a VIGS experiment was conducted for these genes. For this, rose plantlets (*Rosa hybrida* ‘Samantha’) were propagated in tissue culture on Murashige and Skoog (MS) medium supplemented with 3% (w/v) sucrose, 1.0 mg/l 6-benzylaminopurine (6-BA), 0.05 mg/L naphthalene acetic acid (NAA), and 1.0 mg/L GA_3_ for 60 d. Healthy, strong plantlets were transferred to rooting medium comprising half-strength MS medium supplemented with 3% sucrose and 0.1 mg/L NAA for 30 days.

#### Vector construction for VIGS

For construction of the VIGS vectors, 349-, 279-, and 398-bp fragments from the coding sequence region of *RhMLO1*, *RhMLO2*, and *RhMLO3/4* (the fragment is from a region in which *RhMLO3* and *RhMLO4* are identical) were PCR amplified using primers RhMLO1-VIGS-F and RhMLO1-VIGS-R, RhMLO2-VIGS-F and RhMLO2-VIGS-R, RhMLO3/4-VIGS-F and RhMLO3/4-VIGS-R, respectively (Supplementary Table S1). The generated fragments were inserted into the pTRV2 vector as described previously (Liu et al. 2002). The pTRV2, pTRV2-RhMLO1, pTRV2-RhMLO2, and pTRV2-RhMLO3/4 constructs were transformed into *Agrobacterium tumefaciens* strain GV3101. The transformed *A. tumefaciens* strains were cultured in Luria–Bertani (LB) medium supplemented with 10 mM MES, 20 μM acetosyringone, 25 μg/ml rifampicin, and 50 μg/ml kanamycin. The cultures were collected and resuspended in infiltration buffer (10 mM MES, 10 mM MgCl_2_, and 200 μM acetosyringone, pH 5.6) to a final OD_600_ of 1.5. Mixtures of cultures containing an equal ratio (v/v) of pTRV1 (Liu et al. 2002) and each recombinant pTRV2 construct were placed in the dark for 3–5 h at room temperature and were then used for vacuum infiltration.

#### Preparation and analysis of infiltrated plants

Rose plantlets were placed in deionized water for 2 days and then immersed in the bacterial suspension and infiltrated under a vacuum at 0.7 MPa twice. Plantlets infiltrated with an *A. tumefaciens* strain containing the empty virus were used as controls (TRV-00). After infiltration, the plantlets were washed in water five times and in deionized water twice and then placed in the dark at 8 °C for 3 d. The plantlets were transplanted into pots and grown at 23 ± 2 ℃ for 16 h in the light and 8 h in the dark. At 30 days post-vacuum infiltration, RNA was isolated from infiltrated plantlets using the OMEGA Plant RNA Kit (OMEGA, China) to determine the expression of the target gene (*RhMLO1*, *RhMLO2*, or *RhMLO3/4* combined). The *R. hybrida* ubiquitin gene *RhUBI2* (Supplementary Table S1) was used as an internal control. RT-qPCR reactions (10 μl volume) were performed using the KAPA SYBR FAST Universal RT-qPCR kit (Takara, China) in the ABI Real-time PCR System (Thermo Fisher Scientific). Cycling conditions were as follows: 3 min of denaturation at 95 °C, followed by 40 cycles of 95 °C for 3 s, 60 °C for 30 s, and 72 °C for 30 s. Each reaction was performed in triplicate, and products were verified by melting curve analysis. The transcript levels of genes were determined using the 2^−ΔΔ*CT*^ method. The plants with down-regulated target gene expression were retained for further pathogen assays.

#### Powdery mildew infection assays

The *P. pannosa* strain CAU8311, causing powdery mildew in rose, was cultivated on living rose leaves in an illumination incubator. Infected rose leaves were collected in sterilized water and thoroughly vibrated using a vortex mixer to separate *P. pannosa* from the leaves. The *P. pannosa* spore suspension was adjusted to a final concentration of 3 × 10^5^ conidia/ml in sterilized water for foliar application. Approximately 30-day-old infiltrated rose plantlets were inoculated with *P. pannosa*. The plants were transferred into the illumination incubator at 16ºC in the dark for 24 h and then at 22/16 °C, with a 16/8-h light/dark cycle. To test powdery mildew resistance of the infiltrated plants, the average number of visible *P. pannosa* colonies that formed on each plant leaves was evaluated at 7, 10, 13, 16 and 25 days post-inoculation (dpi). A Student’s *t* test was used for statistical analysis. Differences were considered statistically significant at *p* < 0.05 (*) or *p* < 0.01 (**). For fungal biomass analysis of infiltrated plants, at 25 days post-inoculation total DNA was isolated from whole plant leaves and the fungal biomass was determined by RT-qPCR of the *P. pannosa* internal transcribed spacer (ITS) relative to the rose *Actin5* gene *RhActin5*, using primers ITS-F and ITS-R, RhActin5-F and RhActin5-R, respectively (Supplementary Table S1). The RT-qPCR reaction was performed in an ABI Real-time PCR System (Thermo Fisher Scientific) with cycling conditions: 3 min at 95 °C, followed by 40 cycles of 95 °C for 3 s, 60 °C for 30 s, and 72 °C for 30 s.

### Functional analyses of clade V RhMLO genes using transient RNAi

#### Vector construction for the transient RNAi in petals

As a basis for the RNAi experiments the vector p9U10-RNAi (DNA-cloning service, Hamburg, Germany) was used. In this vector, double stranded RNA is generated by cloning the target fragment between two 35S promoters in reverse orientation (Schmidt et al. 2012). The vector was linearized via BamHI/HindIII double digest and gel-purified to remove a dummy fragment from the GUS gene cloned between the two 35S promoters. Fragments from the most variable last exon of the four *RhMLO* genes (283 bp for *RhMLO1*, 291 bp for *RhMLO2*, and 311 bp for *RhMLO*3/4, Supplementary Figure S3) were amplified via proof reading PCR with overhangs specific to the BamHI/HindIII restriction site of the vector. Cloning into the vector was then performed using the In-Fusion™ HD Cloning Plus Kit (Takara Bio, Mountain View, USA). Plasmids were transformed into *E. coli* DH10B by electroporation, and after isolation of plasmids, the sequences were checked via custom Sanger sequencing (GATC, Cologne, Germany). Plasmids with correctly inserted MLO fragments were then electroporated into *Agrobacterium tumefaciens* strain GV3101 via electroporation.

#### Transient RNAi silencing in rose petals

Fresh streaks of the Agrobacterium strains harbouring either GUS (negative control), *RhMLO1*, *RhMLO2* or *RhMLO3/4* RNAi constructs were precultured in 10 ml YEP medium overnight. From these cultures Erlenmeyer flasks with 25 ml each were inoculated with 200 µl of the overnight culture and incubated with shaking at 28 °C overnight. From this culture a third culture was inoculated the next day so that the starting OD_600_ was 0.2 and then grown until an OD_600_ of 1.0 to 1.5 was reached. The final cultures were centrifuged for 1 h at 3500 rpm, and the pellet was resuspended in infiltration buffer (2.13 g/l MES, 2.03 g/l MgCl2·6H2O, pH 5.6) at an OD_600_ of 0.5. Prior to the infiltrations the suspension of each MLO construct was mixed in equal amounts with the suspension of a strain harbouring an empty pRedU10-35 s construct expressing dsRed under the Arabidopsis Ubiquitin promoter (the same culture conditions were used). Prior to the infiltration petals of the variety Pariser Charme were harvested from unopened floral buds and placed on wet filter paper in translucent plastic boxes. A small incision was placed at the base of the petal through which the suspension was infiltrated into the petal with a 1-ml syringe without needle. The boxes were incubated at 20 °C for 6 days before they were inoculated with conidia of rose powdery mildew sampled from infected greenhouse plants.

Only those areas of the infiltrated petals were sampled for either qPCR or for the disease assay that expressed the dsRed signal approx. 6 days after infiltration.

#### qPCR of infiltrated petals

For qPCR 100 mg of dsRed positive parts of the petals were excised and frozen in liquid nitrogen. RNA was extracted using the RNeasy® Plant Mini Kit (Qiagen, Germany) according to manufacturer’s instructions, but using DDT instead of beta-mercaptoethanol. DNA was digested using the DNA-free™ DNA Removal Kit (Ambion, USA). For cDNA synthesis, the High-Capacity cDNA Reverse Transcription Kit (Applied Biosystems USA) was used. Quantitative real-time PCR was conducted using the Takyon Rox SYBR Master-Mix dTTP Blue kit (Eurogentec, Belgium) (10 µl reaction volume) and cycling conditions: 95 °C 1 min, followed by 40 cycles of 95 °C 10 and 59°-64 °C 60 s (StepOnePlus cycler from Applied Biosystems, USA). Each infiltration was repeated four times, and in each experiment, three petals were sampled and combined into one RNA pool. Each reaction was performed in triplicate, and the melting curve was used for product verification. As internal controls, the genes UBC and SAND were used.

The obtained data were used for primer efficiency calculation of each reaction using LinRegPCR software (Version 2016.1, Ramakers et al. 2003). Based on this, the RQ value was determined using R (version 3.2.4, R Core Team 2016). Data were log-transformed and on the basis of a linear model, significant differences in mean comparison and ANOVA analysis to the control were determined (*α* = 0.05). The *p *values of the mean comparison were adjusted using the Dunnett test.

#### Infection assay on rose petals

Powdery mildew conidia were obtained from naturally infected greenhouse plants by washing them off infected leaves in distilled water with 0.05% Tween 20. The concentration of conidial suspensions was determined in counting a sample with a Fuchs-Rosenthal chamber and adjusted to a concentration of 500,000 conidia/ml. Suspensions were sprayed onto petals using an atomizer followed by immediately drying the liquid in the airstream of a fume hood. After drying of petal surfaces was complete, vitality of a suspension kept in an Eppendorf tube was checked by staining with phenosafranin. Samples were taken 3 dpi by cutting out those parts of the petal that displayed dsRed fluorescence. Samples were fixed on filter paper soaked in ethanol/acetic acid (3:1) with the inoculated side up. Fixed samples were washed 3 × in PBS buffer avoiding contact of the inoculated area with the buffer and then stained with the WGA-AlexaFlour488 stain (Invitrogen, Carlsbad, USA) at 10 µg/ml in PBS. Microscopy was done with a Zeiss AxioScope A1 (Zeiss, Jena, Germany) with the filter set 38 (excitation: 493 nm, emission: 520 nm) at a magnification of 100 × and 200x. Four independent experiments at different dates using independently prepared inoculum were conducted, each comprising 6 (experiment1) or 8 (experiments 2–4) petals. All germinated spores were counted on the excised area of the petal, and the fraction of those which formed a mycelium was determined for each petal per experiment. An ANOVA was computed in R based on a generalized linear model, assuming a Poisson distribution for the ratio of colonies formed in relation to the number of germinated conidia. This was followed by a Dunnett post hoc test for significance in pairwise comparisons between the GUS control and the RNAi constructs.

## Results

### In silico* identification of the rose RhMLO gene family*

A blast search for *MLO* homologs of the *R. chinensis* genome sequence with *F. vesca*, *P. persica*, *M. domestica* and *A. thaliana MLO* gene models produced 19 significant matches in rose (Table 2, Supplementary Excel File S1). We named them *RhMLO1*-*19* (tentative names for *RhMLO5-19*). Generally, for each of the *MLO* genes from the other species there was one highly similar counterpart gene in rose, except for *RhMLO3* and *RhMLO4*, both on chromosome 1, which have their highest similarity both to *FvMLO12*, although below 90% similarity. The two genes are only 86.8% similar to each other, and their gene structure is also different.

**Table 2:** Members of the *RhMLO* gene family in *Rosa chinensis* ‘Old Blush’

Gene	Accession number (Rosa chinensis V1.0)	Chr	Start position	End position	Length (aa)	*R. multiflora* orthologs (identity %)	*F. vesca* orthologs (identity %)	Chr. *of F. vesca* orthologs	*P. persica* orthologs (identity %)	*M. domestic*a orthologs (identity %)	Arabidopsis orthologs (identity %)
*RhMLO1*	RC5G0516400	5	59,433,529	59,440,731	591	RhMLO1 (99.49)	FvMLO1 (FvH4_6g15610.1) (94.04)	6	PpMLO1 (85.88)		
*RhMLO2*	RC3G0207400	3	29,087,200	29,091,431	588	RhMLO2 (99.15)	FvMLO4 (93.19)	6	PpMLO3 (79.59)	MdMLO11 (74.49) MdMLO19 (76.34)	
*RhMLO3*	RC1G0418100	1	51,670,283	51,673,832	563	RhMLO3 (98.76)	FvMLO12 (88.15)	7	PpMLO4 (70.93)	MdMLO5(68.62) MdMLO7(68.23)	AtMLO2 (63.90) AtMLO6 (62.09) AtMLO12 (62.61)
*RhMLO4*	RC1G0417800	1	51,642,152	51,645,544	519	RhMLO4 (94.22)	FvMLO12 (83.06)	7	PpMLO4 (73.01)	MdMLO5(70.44) MdMLO7(71.74)	AtMLO2 (64.74) AtMLO6 (64.11) AtMLO12 (67.89)
*RhMLO5*	RC2G0000700	2	111,682	118,694	519		FvMLO5(74.91) FvMLO6 (91.33) FvMLO7(80.645)	1 3 3	PpMLO6 (72.24) PpMLO17(64.26)	MdMLO1(61.931) MdMLO6(63.14)	AtMLO15 (57.74)
*RhMLO6*	RC1G0223800	1	30,491,553	30,500,060	411		FvMLO2 (62.07)	7	PpMLO10 (68.45)	MdMLO6(61.75) MdMLO9(60.68) MdMLO13(73.08)	AtMLO15 (54.44)
*RhMLO7*	RC3G0207200	3	29,055,941	29,060,557	541		FvMLO3 (90.78)	6	PpMLO8(71.96) PpMLO18 (62.24)	MdMLO16 (71.74)	
*RhMLO8*	RC6G0428300	6	53,802,760	53,804,437	508		FvMLO8 (88.65)	2	PpMLO7 (76.73)	MdMLO6 (72.93)	AtMLO1 (73.52) AtMLO15 (58.58)
*RhMLO9*	RC7G0392800	7	39,644,544	39,651,717	562		FvMLO9(79.51) FvH4_5g28810.1 (94.66)	5	PpMLO11 (88.34)		AtMLO4 (69.59)
*RhMLO10*	RC2G0114500	2	9,454,443	9,460,124	587		FvMLO10 (91.09)	1	PpMLO2 (78.67)	MdMLO4(74.18) MdMLO15(62.22)	AtMLO7 (60.76) AtMLO8 (61.79) AtMLO10 (65.85)
*RhMLO11*	RC2G0135000	2	11,262,809	11,266,568	550		FvH4_1g11630.1 (78.57)	1	PpMLO15 (73.72)	MdMLO2(62.34) MdMLO3(60.17) MdMLO8(54.55)	
*RhMLO12*	RC7G0349700	7	33,341,027	33,345,063	516		FvH4_5g25860.1 (82.63)	5	PpMLO16 (78.34)	MdMLO12 (71.24)	AtMLO5 (59.39) AtMLO9 (62.93)
*RhMLO13*	RC3G0132400	3	21,150,054	21,156,558	641		FvMLO13 (76.16)	7	PpMLO13 (79.34)	MdMLO20 (58.243)	
*RhMLO14*	RC5G0517300	5	59,600,438	59,605,166	624		FvMLO14 (FvH4_3g32830.1) (75.00)	3	PpMLO14 (67.29)	MdMLO21 (63.18)	AtMLO3 (46.745)
*RhMLO15*	RC5G0086800	5	6,344,433	6,348,265	521		FvMLO15 (91.62)	3	PpMLO9 (80.24)	MdMLO18 (75.94)	
*RhMLO16*	RC5G0301800	5	26,801,289	26,808,352	552		FvMLO16 (84.13)	3	PpMLO5 (85.71)	MdMLO10(82.80) MdMLO17(75.36)	AtMLO11 (69.57) AtMLO14(69.22)
*RhMLO17*	RC7G0349100	7	33,266,427	33,271,498	567		FvMLO17 (FvH4_5g25790.1) (81.44)	5	PpMLO12 (77.28)		
*RhMLO18*	RC7G0367400	7	35,516,970	35,521,212	499		FvMLO18 (74.84)	5	PpMLO19 (65.67)	MdMLO14 (60.56)	AtMLO13 (66.00)
*RhMLO19*	RC4G0104700 RC4G0104600	4	13,127,278	13,140,269	524		FvH4_5g25790.1 (84.26)	5			

In addition, one of the matched sequences from *R. chinensis*, RC4G0104600, encoded a protein of 321 amino acids, which is much shorter than all *MLO* homologs reported in, for example, the genomes of Arabidopsis and grapevine (Devoto et al. 2003; Feechan et al. 2008). The adjacent annotated sequence, RC4G0104700, is also a part of an *MLO* gene. We have merged them in order to recreate a full-length *MLO* gene, called *RhMLO19*. Details on genomic localization, length of the predicted proteins, and the level of similarity to other known *MLO* homologs in this study are provided in Table 2.

**Fig. 1:**
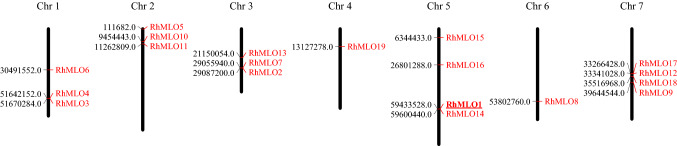
Physical position of 19 *RhMLO* genes on *Rosa chinensis* ‘Old Blush’ chromosomes. *RhMLO1* on chromosome 5, which has been functionally characterized as a susceptibility gene, is underlined. The figure was generated using MapChart2.32

The physical position of *RhMLO1-RhMLO19* across all seven *R. chinensis* chromosomes is depicted in Fig. 1. We found four cases of adjacent genes (*RhMLO3* and *RhMLO4*, *RhMLO2* and *RhMLO7*, *RhMLO1* and *RhMLO14*, *RhMLO12* and *RhMLO17*). Except for *RhMLO3* and *RhMLO4* the pairs of adjacent genes were, however, not from the same clade (see below).

As reported previously, the orthologous relationship between rose and strawberry is very close (Shulaev et al. 2010; Hibrand Saint-Oyant et al. 2018). We made a synteny analysis between *R. chinensis* ‘Old Blush’ and *F. vesca* chromosomal regions containing *MLO* homologs. The locations of 15 of the 19 *RhMLO* homologs (Table 2) are in accordance with known syntenic relations of the chromosomes. *RhMLO5* on chromosome 2 showed homology to both *FvMLO6* and *FvMLO7* on chromosome 3, which is unexpected as rose linkage group 5 is syntenic to *F. vesca* linkage group 3. In addition, the chromosome locations of *RhMLO1, RhMLO13* and *RhMLO19*, located, respectively, on chromosome 5, 3 and 4, are homologous to *FvMLOs* on chromosome 6, 7 and 5, respectively. This is surprising as rose linkage group 3, 4 and 5 are syntenic to *F. vesca* linkage group 6, 4, and 3, respectively (Vukosavljev et al. 2016; Hibrand Saint-Oyant et al. 2018).

### Phylogenetic relations of the rose RhMLO gene family

**Fig. 2:**
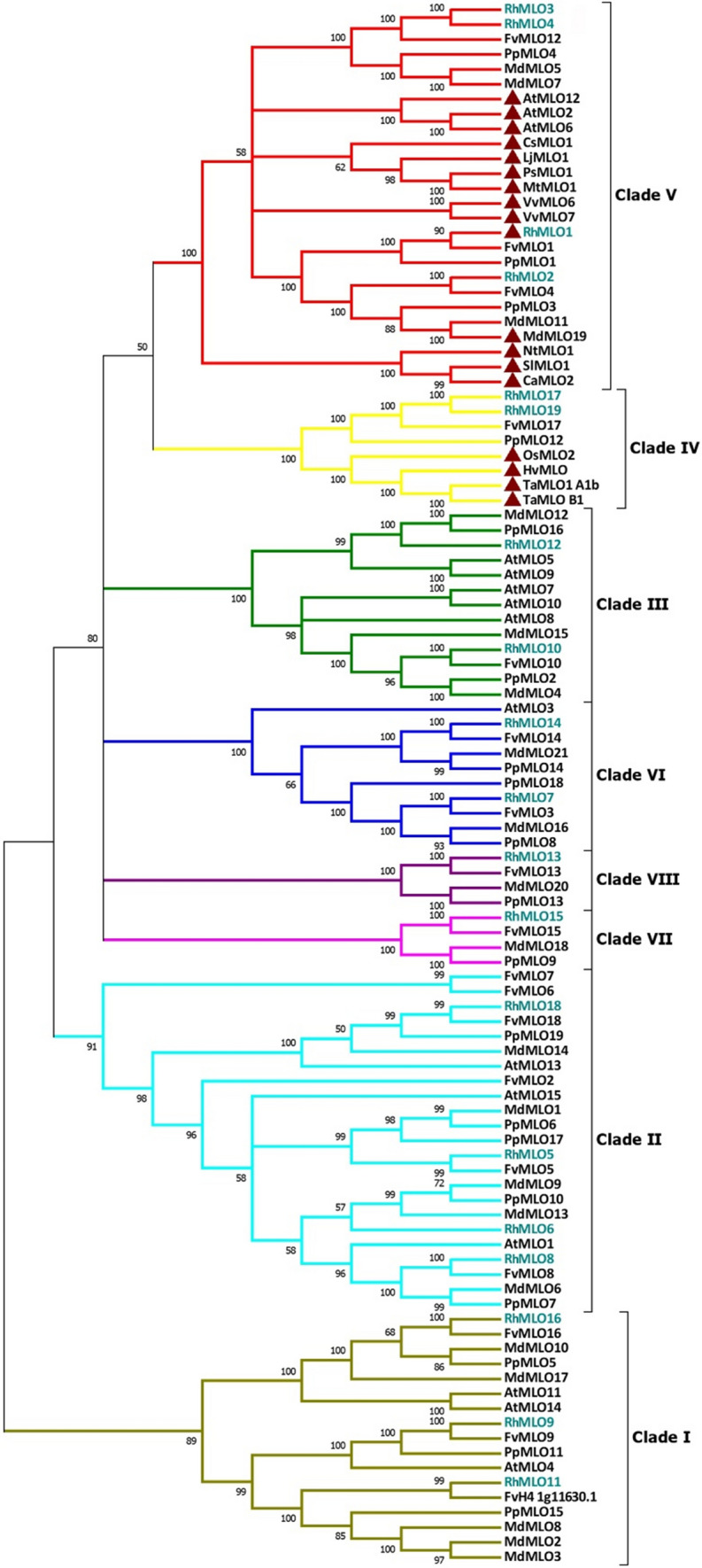
Phylogenetic tree of *RhMLO* genes. Phylogenetic relationships of predicted RhMLO amino acid sequences to MLO proteins of other pant species including several Rosaceae *MLO* (Pessina et al. 2014; Edger et al. 2018) and some dicot (Bai et al. 2008; Feechan et al. 2008; Winterhagen et al. 2008; Humphry et al. 2011; Zheng et al. 2013; Berg et al. 2015; Nie et al. 2015; Pessina et al. 2016b) and monocot *MLO* genes (Büschges et al. 1997; Elliott et al. 2002). 15 *AtMLO* amino acid sequences were obtained from The Arabidopsis Information Resource (TAIR, http://www.arabidopsis.org/). Displayed is only topology and cut-off value for the consensus tree to 50%. The 19 *RhMLOs* are highlighted in blue. Proteins that have previously been functionally characterized as susceptibility genes are marked by red triangles. Numbers at each node represent bootstrap support values (1000 replications)

A phylogenetic analysis was performed to establish the relationship between the *RhMLO* proteins and the *MLO* proteins of several other plant species (*Arabidopsis thaliana*), peach (*Prunus persica*), strawberry (*Fragaria vesca*), apple (*Malus domestica*), tomato (*Solanum lycopersicum*), pepper (*Capsicum annuum*), cucumber (*Cucumis sativus*), tobacco (*Nicotiana tabacum*), pea (*Pisum sativum*), barrelclover (*Medicago truncatula*), lotus (*Lotus japonicus*), grapevine (*Vitis vinifera*), barley (*Hordeum vulgare*), wheat (*Triticum aestivum*) and rice (*Oryza sativa*). The UPGMA (Fig. 2) and ML (not shown) trees had similar topology. The UPGMA tree contains eight different clades named from I to VIII following the classification of previous studies in Arabidopsis and *Rosaceae MLO* protein families (Feechan et al. 2008; Pessina et al. 2014). Four *RhMLO* homologs (*RhMLO1*, *RhMLO2*, *RhMLO3* and *RhMLO4*) were found in Clade V, which includes all dicot *MLO* proteins shown to be required for powdery mildew susceptibility (Consonni et al. 2006; Bai et al. 2008; Humphry et al. 2011; Zheng et al. 2013; Berg et al. 2015; Nie et al. 2015; Qiu et al. 2015a, b; Fujimura et al. 2016; Pessina et al. 2016a, b). Two homologs from rose (*RhMLO17* and *RhMLO19*) were found in Clade IV, which contains the MLO proteins acting as susceptibility factor in monocots.

### Structural organization of RhMLO genes

**Fig. 3:**
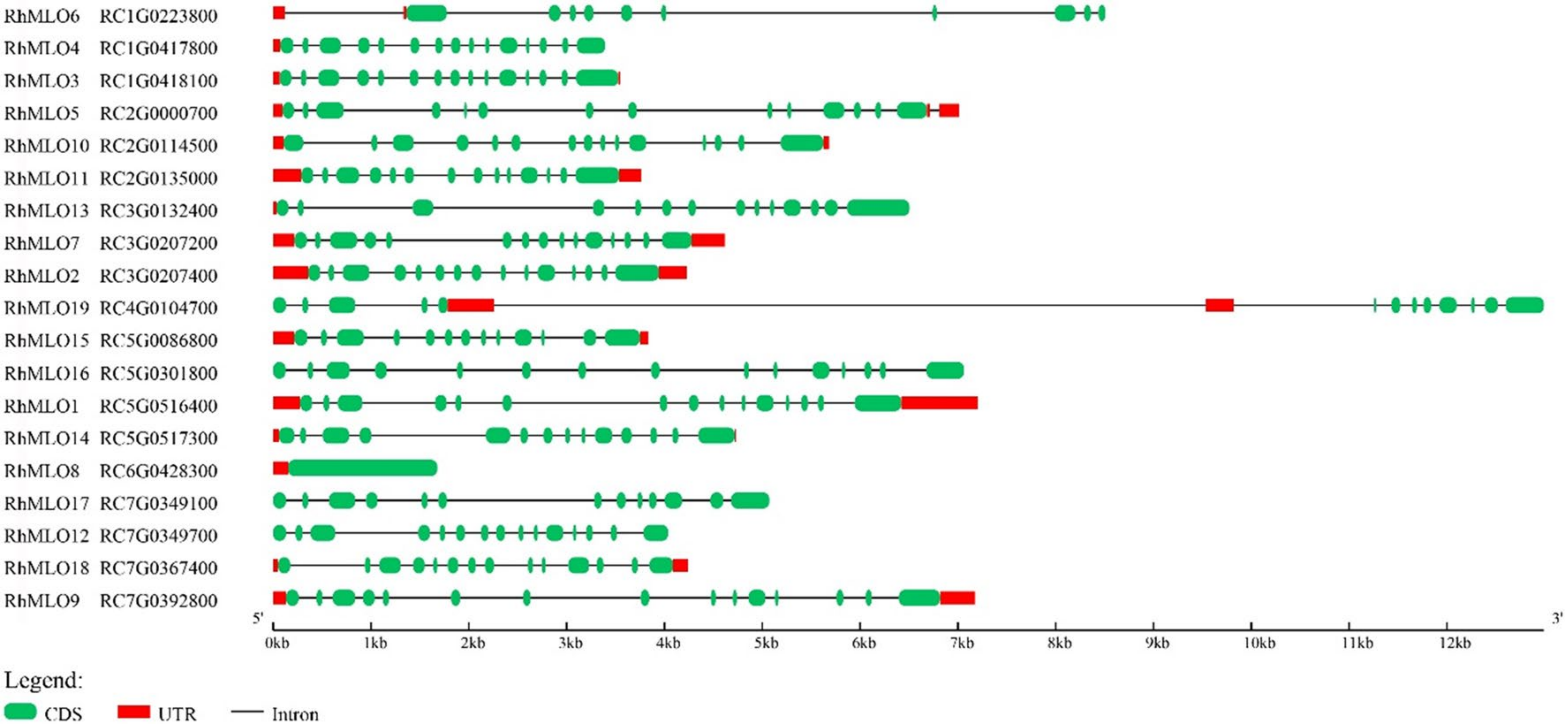
Gene structure of 19 rose *MLO* genes in the R. chinensis genome sequence. Gene models (5′–3′) were derived from the GFF3 file at https://www.rosaceae.org/species/rosa/chinensis/genome_v1.0. The gene structure of RhMLO19 was based on the combination of RC4G0104700 and RC4G0104600

All *RhMLO* genes had seven transmembrane domains, except for *RhMLO9*, *RhMLO13* and *RhMLO19* that have six (Table 3). In all proteins except *RhMLO6* we detected a conserved calmodulin-binding site (CaMBD). Furthermore, in some proteins (RhMLO1, RhMLO2, RhMLO10, RhMLO17 and RhMLO19) the conserved domain II (D/E-F-S/T-F) was detected in the highly polymorphic C terminal part as previously found by Panstruga (2005) (Supplementary Figure S1**)**. Most *MLO* genes consist of 13–15 exons except *RhMLO6* (11 exons) and *RhMLO8* (1 exon) (Fig. 3). The single exon structure of *RhMLO8* is an exception within the *MLO* family in rose, but its homologs in apple and peach also consist of a single exon.

**Table 3:** Structural organization of the transmembrane domains (TMs) of the predicted RhMLO proteins

Gene	Location of TM1 (aa)	Location of TM2 (aa)	Location of TM3 (aa)	Location of TM4 (aa)	Location of TM5 (aa)	Location of TM6 (aa)	Location of TM7 (aa)	No of TMs	No of Exons	Length of gDNA (bp)
*RhMLO1*	16–40	61–79	158–179	283–303	309–333	367–391	406–431	7	15	7203
*RhMLO2*	16–39	60–79	165–186	290–309	321–340	374–397	417–438	7	15	4232
*RhMLO3*	15–39	60–81	145–166	270–289	295–320	354–375	395–418	7	15	3550
*RhMLO4*	19–42	63–82	149–170	274–297	303–324	358–382	397–422	7	15	3393
*RhMLO5*	12–33	59–76	160–180	268–287	293–311	352–376	396–416	7	15	7013
*RhMLO6*	16–36	62–79	152–176	261–280	286–305	343–366	386–407	7	11	8508
*RhMLO7*	16–40	60–76	166–187	290–309	315–333	374–397	417–438	7	15	4617
*RhMLO8*	16–36	62–80	145–166	275–294	300–318	356–379	399–420	7	1	1678
*RhMLO9*	20–41	62–80	154–174	274–295	301–318	357–381	–	6	15	7174
*RhMLO10*	41–62	83–104	170–191	293–315	321–343	377–401	421–441	7	15	5682
*RhMLO11*	15–39	60–79	153–174	277–295	301–318	357–380	400–421	7	14	3760
*RhMLO12*	18–45	66–84	163–184	284–303	309–327	368–391	411–432	7	15	4037
*RhMLO13*	15–35	64–88	140–164	274–292	298–316	–	408–428	6	14	6505
*RhMLO14*	16–40	61–83	178–197	333–350	356–373	414–436	475–500	7	14	4729
*RhMLO15*	20–43	64–82	132–154	253–272	278–296	337–361	376–401	7	13	3833
*RhMLO16*	12–33	63–85	157–178	279–297	303–320	359–383	403–423	7	15	7064
*RhMLO17*	20–41	62–81	166–188	290–313	325–343	384–405	411–436	7	13	5072
*RhMLO18*	12–32	62–79	147–168	272–291	297–315	354–377	397–418	7	14	4243
*RhMLO19*	20–41	62–81	–	235–258	270–288	329–348	368–393	6	14	3474

### Expression patterns of RhMLO genes

Gene expression was found in various transcriptome datasets for 11 of the 19 *RhMLO* genes. A heatmap (Supplementary Figure S2) shows that *RhMLO3* and *RhMLO4* were detected at high levels in leaves under water stress, while *RhMLO5*, *RhMLO10* and *RhMLO15* accumulated in young roots. *RhMLO8* was expressed in all floral samples, especially in floral meristem and early floral organ development.

### Allelic variants in wild and cultivated roses

We produced an overview of the allelic variants of the MLO genes across the rose species based on both the available genome sequences and the RNAseq data. Alleles may go undetected in the RNAseq data if they are not expressed, or if read numbers are too low, so this analysis may underestimate the real allelic diversity.

After BLASTp or tBLASTn, a total of 300 different complete or partial sequences were detected across the 19 *RhMLO*s in the 22 rose accessions (Supplementary Table S2 & Supplementary Table S3). Because *RhMLO19* has a high similarity (87.7%) to *RhMLO17*, the sequences that matched with *RhMLO19* all matched with *RhMLO17* as well (Supplementary File 1), and of these only DL_g32145.t1 had a higher similarity to *RhMLO19* (93.8%) than to *RhMLO17* (93.1%). For the sake of the diversity analysis, we have treated all these sequences as putative alleles of *RhMLO17*.

Among the 18 *RhMLO* genes with allele information, *RhMLO1* had the highest number of sequences (23), while *RhMLO4* had the lowest (6). After aligning the DNA sequences, we focused on the occurrence of SNPs among the sequences. For ease of visualisation, sequences were reduced to a concatenated sequence of all SNP positions. The length of these runs of SNPs found for the same *RhMLO* gene in different accessions is dependent on the actual length of sequences found. Therefore, we inserted ‘N’ to fill missing SNP positions in the assembled reads of a particular haplotype (Supplementary Table S4**)** and indicate the position for all SNPs and indels in Supplementary Excel File S2. Some short runs of SNPs were set aside because they were not unique for one haplotype (e.g. CCACCCG in *RhMLO1* could be part of the longer haplotypes H2, H3, H7 or H8). Resolving these short runs will require generating longer sequence reads.

From the 300 sequences, a total of 198 different SNP haplotypes, further referred to as allelic variants or alleles, were found with an average of 10 alleles per gene. *RhMLO8* and *RhMLO18* showed the highest number of alleles (putatively 16 alleles). To find more information about the relationship among the rose accessions, the sharing of identical alleles deserves closer attention. Allele H1 in *RhMLO1* was present in diploid species *R. chinensis cv.‘Old Blush’* and *R. chinensis var. spontanea*, together with tetraploid garden roses AH and MF. This is consistent with the notion that *R. chinensis var. spontanea* is one of the ancestors of *R. chinensis cv.‘Old Blush’* and that these diploid species from the section Chinenses have contributed to the makeup of tetraploid modern roses. The situation that a SNP haplotype was found in both a diploid species and a tetraploid cultivar occurred also for *RhMLO2*, *RhMLO4*, *RhMLO5*, *RhMLO6*, *RhMLO7*, *RhMLO8*, *RhMLO9*, *RhMLO10* and *RhMLO15* (Supplementary Table S4). *RhMLO8* and *RhMLO9* were found in all 22 *Rosa* accessions (Supplementary Table S4) and are thus best covered and most suitable to check for common haplotypes between diploid species and tetraploid cultivars.

### Specific analysis of the RhMLO genes in clades IV and V

For *RhMLO1*, among the 271 detected SNPs, we observed 25 non-synonymous SNPs and two indels with an effect on the resulting amino acid sequence (alignment in Supplementary PDF S1). Some amino acid changes were caused by two or three sequential SNPs, such as Y34L (TAC → TTA) and I35C (ATA → TGT). One insertion (+ 105E) and one deletion (Q512-) were in non-conserved regions.

For *RhMLO2*, among 161 detected SNPs, we found 17 non-synonymous SNPs and two indels (Supplementary PDF S2). The first indel with a 36-bp deletion was in a conserved region (NAFQLAFFAWTW386-). The other 12-bp deletion (HTRD570-) was in non-conserved regions, but the amino acid asparagine (D) belongs to the C-terminal D/E-F-S/T-F tetra-peptide sequence, which is one of several motif characteristic of *MLO* orthologs.

For *RhMLO3*, among 337 detected SNPs, we found 39 non-synonymous SNPs and five indels (Supplementary PDF S3). Two insertions, namely + 134 T and + 464L, were in conserved regions. For *RhMLO4*, among 142 detected SNPs, we found 15 non-synonymous SNPs and no indels (Supplementary PDF S4). For *RhMLO17*, among 78 detected SNPs, we found 8 non-synonymous SNPs and one indel (Supplementary PDF S5). The only deletion (VVVGIS290-) was in a conserved region.

### RhMLO1 gene silencing in rose using VIGS

**Fig. 4:**
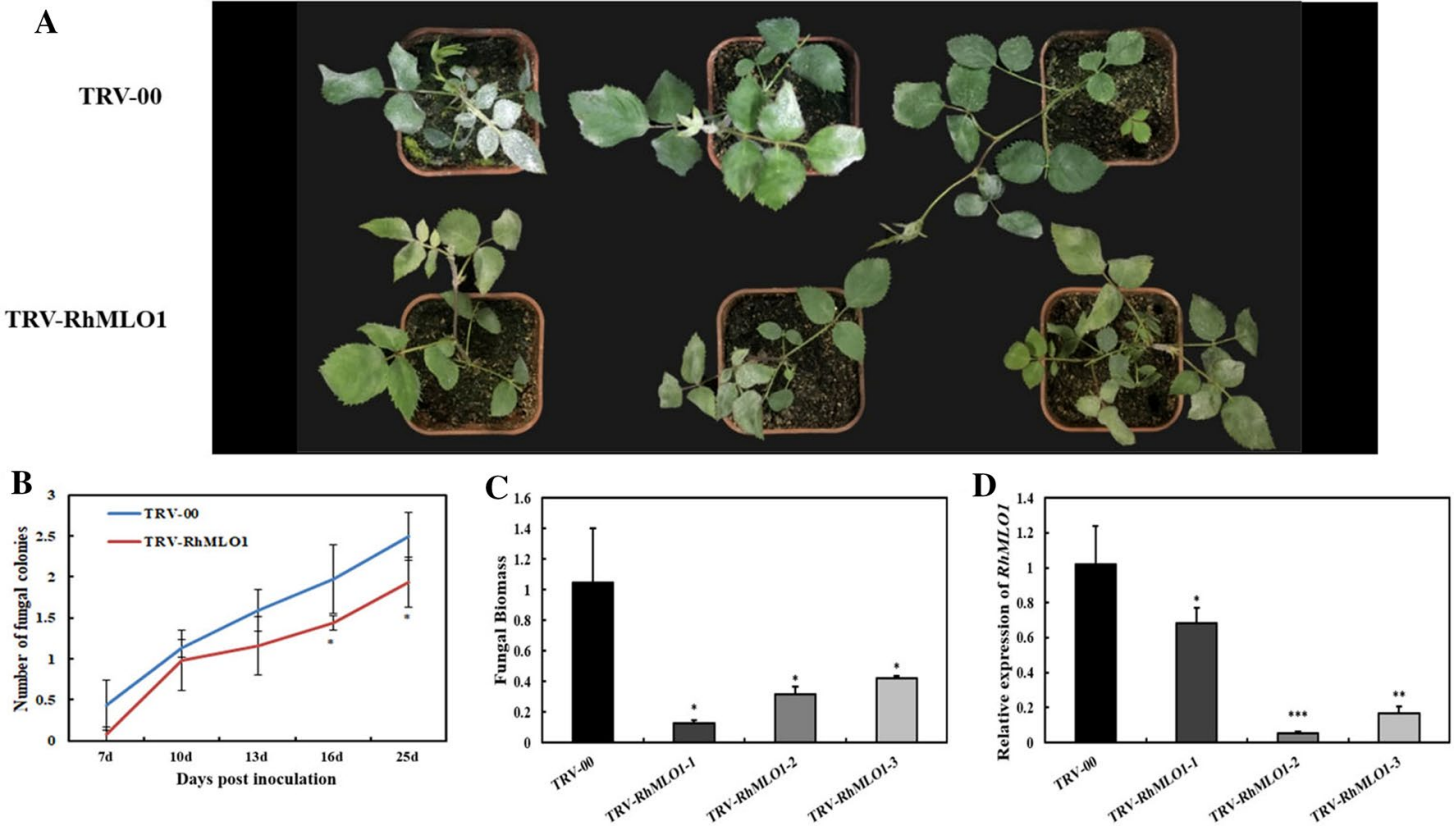
Silencing *RhMLO1* in rose enhances resistance to powdery mildew. **a** Typical appearance of control (TRV-00) and TRV-RhMLO1-infiltrated rose plants upon inoculation with powdery mildew 25 days post-inoculation (dpi). **b** Quantification of powdery mildew symptoms in control (TRV-00) plants and TRV-RhMLO1-infiltrated plants, shown as the average number of fungal colonies on each rose leaf. **c** Fungal biomass determined by RT-qPCR in control (TRV-00) and three independent lines of TRV-RhMLO1-infiltrated rose plants. Bars represent *P. pannosa* ITS transcript levels relative to rose *RhActin5* transcript levels for equilibration. Lines 1, 2, and 3 represent three independent TRV-RhMLO1-infiltrated plants. The samples were taken at 25 dpi. **d** RT-qPCR was performed to monitor expression of *RhMLO1* in both control (TRV-00) and TRV-RhMLO1-infiltrated rose plants (TRV-*RhMLO1–1*, *1–2*, and *1–3*). The total RNA was isolated from infiltrated plantlets at 30 days post-vacuum infiltration, prior to powdery mildew inoculation. The rose ubiquitin gene *RhUBI2* was used as an internal control. Asterisks indicate significant differences according to Student’s *t* test (*P < 0.05, **P < 0.01, ***P < 0.001)

To test the practicality of using VIGS to study the *MLO* gene function in rose for powdery mildew resistance, we set out at CAU in Beijing to suppress the expression of the endogenous *RhMLO1* gene, which was suspected to play an important role in the susceptibility of rose towards powdery mildew (Qiu et al. 2016). To this end, the 349-bp *RhMLO1* sequence was cloned and used. Compared to control plants treated with an empty TRV vector (TRV-00), plants infiltrated with TRV-RhMLO1 showed an increased resistance against powdery mildew, evidenced by decreased fungal growth (Fig. 4a, b) and biomass (Fig. 4c). Prior to the pathogen inoculation, RT-qPCR of independently infiltrated plants showed a significant decrease in the abundance of *RhMLO1* transcripts in the leaves of plants treated with TRV-RhMLO1 at 30 days post-vacuum infiltration (Fig. 4d). The suppressed growth of powdery mildew on the VIGS-silencing rose plants resembled what could be seen on plants with stable RNAi down-regulation of endogenous rose *RhMLO1* (Qiu et al. 2016). This further confirmed the involvement of *RhMLO1* in powdery mildew susceptibility of rose. Importantly, these results also highlight the feasibility of using this VIGS method for studying the function of *MLO* genes and powdery mildew resistance in rose.

### RhMLO2 is involved in powdery mildew susceptibility

**Fig. 5:**
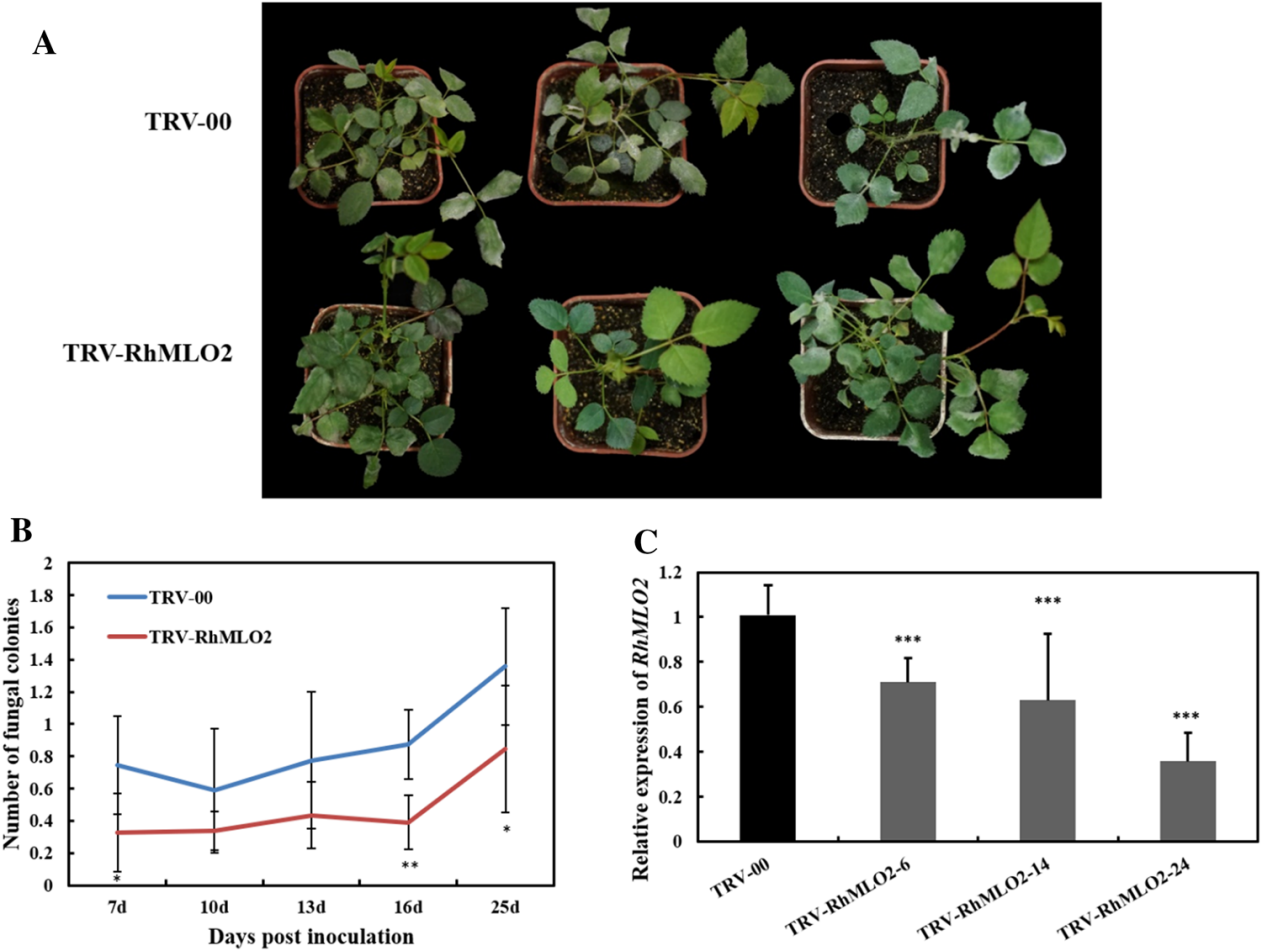
Silencing *RhMLO2* in rose enhanced resistance to powdery mildew. **a** Typical appearance of control plants and rose plants infiltrated with TRV-RhMLO2 upon inoculation with powdery mildew at 25 dpi. **b** Quantification of powdery mildew symptoms in TRV-RhMLO2-infiltrated and control plants. Quantification of symptom development is shown as an average number of fungal colonies on each rose leaf. **c** RT-qPCR was performed to monitor expression of *RhMLO2* in both control (TRV-00) and TRV-RhMLO2-infiltrated plants. The total RNA was isolated from infiltrated plantlets at 30 days post-vacuum infiltration, prior to powdery mildew inoculation. The rose ubiquitin gene *RhUBI2* was used as an internal control. Asterisks indicate significant differences according to Student’s *t* test (*P < 0.05; **P < 0.01; ***P < 0.001)

*RhMLO2* is a close homolog of *RhMLO1*. As the function of *RhMLO2* in powdery mildew infection in rose has not been previously reported, we assessed its role also by VIGS. At 25 days post-infection, fewer fungal colonies were observed in *R. hybrida* ‘Samantha’ plantlets treated with TRV-RhMLO2 (Fig. 5a) compared to control plants treated with the empty vector TRV-00. The symptom development was quantified by evaluating the average number of fungal colonies on each rose leaf (Fig. 5b). The abundance of the *RhMLO2* transcript was substantially reduced in TRV-RhMLO2-treated plants (Fig. 5c), suggesting that *RhMLO2* is also involved in susceptibility of rose towards powdery mildew.

### *RhMLO3* and *RhMLO4* are likely not required for powdery mildew infection in rose

**Fig. 6:**
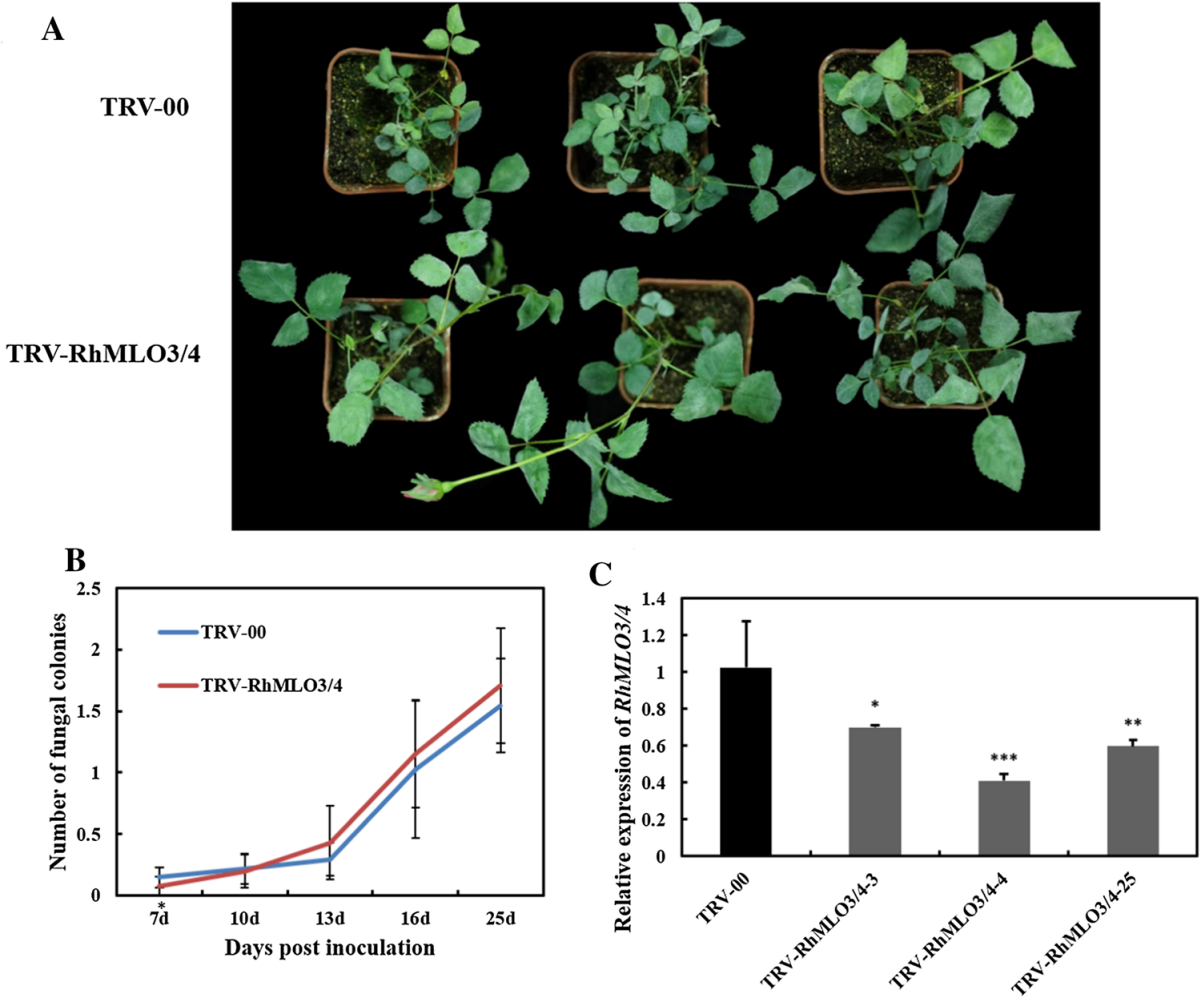
Silencing *RhMLO3/4* in rose does not affect resistance to powdery mildew. **a** Typical appearance of control (TRV-00) and TRV-RhMLO3/4-infiltrated rose plants upon inoculation with powdery mildew at 25 dpi. **b** Quantification of powdery mildew symptoms in control (TRV-00) plants and in different independent TRV-RhMLO3/4-infiltrated plants, shown as the average number of fungal colonies on each rose leaf. **c** RT-qPCR was performed to monitor expression of *RhMLO3/4* in both control (TRV-00) and in different independent TRV-RhMLO3/4-infiltrated rose plants. The total RNA was isolated from infiltrated plantlets at 30 days post-vacuum infiltration, prior to powdery mildew inoculation. The rose ubiquitin gene *RhUBI2* was used as an internal control. Asterisks indicate significant differences according to Student’s *t* test (*P < 0.05, **P < 0.01, ***P < 0.001)

We also assessed whether *RhMLO3* and *RhMLO4* are involved in rose powdery mildew susceptibility using VIGS. *RhMLO3* and *RhMLO4* share 95.5% sequence identity. Therefore, a 398-bp coding sequence shared by *RhMLO3* and *RhMLO4* was cloned into the vector pTRV2 to generate TRV-RhMLO3/4. The powdery mildew resistance of plants infiltrated with TRV-RhMLO3/4 was then assessed by quantifying the number of powdery mildew colonies per leaf following fungal inoculation. RT-qPCR confirmed that the abundance of the *RhMLO3* and *RhMLO4* transcripts was substantially reduced in the leaves of different independent plants infiltrated with TRV-RhMLO3/4 (Fig. 6c). However, there was no significant decrease in fungal colony numbers upon *RhMLO3/4* silencing (Fig. 6a, b), suggesting that *RhMLO3* and *RhMLO4* may be not involved in powdery mildew resistance in rose.

### RNAi silencing

**Fig. 7:**
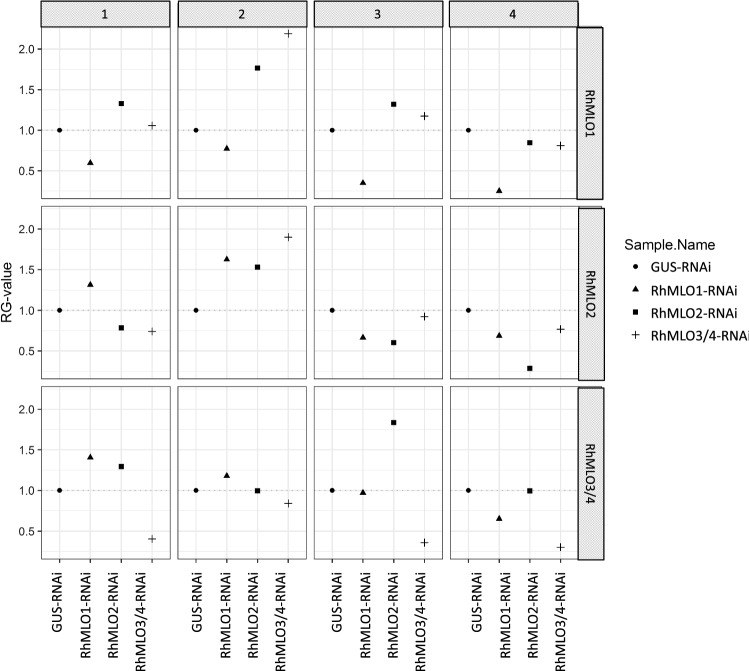
Analysis of gene expression via qPCR for three RNAi constructs for *RhMLO* genes. Columns depict the four repeats, rows the expression values for *RhMLO1*, *RhMLO2* and *RhMLO3/4*. The sample names identify the RNAi silencing constructs used in each experiment

To gain independent evidence for the role of *RhMLO1-4* in the interaction with powdery mildew using another approach, we transiently silenced them by RNA interference in petals of the susceptible variety Pariser Charme at the LUH in Hannover. For this we infiltrated RNAi-constructs for *RhMLO1*, *RhMLO2*, *RhMLO3/4* and *GUS* as a control, each mixed with a dsRed construct, into petals. Two days after infiltration petals were inoculated with powdery mildew. Six days after infiltration areas with a strong dsRed signal were excised and used for gene expression analysis via qPCR (Fig. 7). This was repeated four times independently, each time with fresh cultures of the infiltration constructs. For RhMLO1- and RhMLO3/4-RNAi constructs a partial but significant downregulation of the target *RhMLO* genes could be demonstrated in relation to the GUS negative control (Fig. 7), whereas the downregulation of RhMLO2 expression was not significant although it showed a lower average compared to the control (Figure S4). The respective non-target *RhMLO*s were not affected by the treatments.

Six to nine infiltrated petals were inoculated two days after infiltration for each infiltration experiment and, as for the gene expression analysis, only parts of the petals expressing dsRed were sampled six days after infiltration (four days after inoculation). Statistical analysis of all four experiments together with an ANOVA and a Dunnett test for post hoc analyses revealed a statistically significant reduction of the infection in case of the RNAi constructs for *RhMLO1* as well as *RhMLO2* but only a small, not significant reduction for *RhMLO3/4*, thereby confirming the VIGS experiments (Table 4).

**Table 4:** Statistical analysis of the effect of transient *RhMLO* silencing in petals on mycelium growth. Dunnett test on the ratio of mycelial colonies formed by the number of germinated conidia

Contrast	Estimate	SE	Df	*Z* ratio	*p* value
RhMLO1-RNAi–GUS-RNAi	− 0.6743736	0.2347207	3	− 2.873	0.0116*
RhMLO2-RNAi–GUS-RNAi	− 0.5814200	0.2310675	3	− 2.516	0.0329*
RhMLO3/4-RNAi–GUS-RNAi	− 0.4017875	0.2133053	3	− 1.884	0.1517

The column “estimate” describes the extent of the effect of the means for treatment versus control

*p < 0.05

## Discussion

### Genomic organization and evolution between Rosaceae *MLO* homologs

We identified 19 *MLO* homologs in the reference genome of rose through an in silico approach. This number is in line with other genome-wide studies in dicotyledonous species, which found 18 *MLO* homologs in the genome of strawberry, 19 in peach and 15 in Arabidopsis (Devoto et al. 2003; Pessina et al. 2014).

Consistent with the high level of synteny between the rose and woodland strawberry genomes, 15 probable orthologous relationships between *MLO* genes in these two closely related species are in syntenic positions, while four are not. Whether assembly errors play a role remains to be determined.

We found four cases of adjacent homologs. *RhMLO3* and *RhMLO4* may be the result of a recent gene duplication as both of them had their highest similarity to the same *MLO* gene in *F. vesca* (*FvMLO12*). If so, they have already diverged somewhat in their expression pattern, according to our analysis of the ROSAseq data. *RhMLO1* on Chr. 5 is most similar to *FvMLO1* (which might be FvH4_6g15610.1 in *F. vesca* Genome v4.0.a1) on Chr. 6, but these positions are not syntenous, while the adjacent gene *RhMLO14* is in a syntenic position with *FvMLO14* (which might be FvH4_3g32830.1 in *F. vesca* Genome v4.0.a1) on Chr. 3. It may be useful to screen these regions for microhomology, to differentiate putative translocations from assembly errors. For the other two pairs (*RhMLO2* and *RhMLO7*, *RhMLO12* and *RhMLO17*), the genes with the highest similarity in *F. vesca* are also adjacent to each other.

### *RhMLO* homologs and susceptibility to powdery mildew

The expression patterns of *MLO* genes may indicate possible functions of these genes. Previously, Qiu et al. (2015a) found that the leaf expression levels of *RhMLO1* and *RhMLO2* increased more than two folds on treatment with various abiotic stimuli, while *RhMLO3* and *RhMLO4* decreased. Xiang et al. (2019) identified *RhMLO genes* from *Rosa gigantea* and *Rosa longicuspis*, respectively. The two genes which are homologs of *RhMLO1/2* were drastically up-regulated upon powdery mildew inoculation, which is in agreement with observation of other studies showing up-regulated expression of the *MLO S* gene in different plant species (e.g. Zheng et al. 2013, 2016; Pessina et al. 2016a, b). The functionality as an *S* gene for powdery mildew in rose was confirmed for *RhMLO1* and *RhMLO2* by our VIGS experiments as well as by our experiments using transient RNAi. The two sets of experiments were conducted in two different laboratories using different rose genotypes, using different sources of powdery mildew inoculum, different methods for reducing gene expression, and independent biological replications of the experiments rather than technical replications of the measurements.

All results showed a significant effect except the transient RNAi-downregulation of *RhMLO2*, which, although showing a tendency for reduction, was statistically not significant due to the very low basic expression level of *RhMLO2* and the large variation across the four independent experiments conducted. Nevertheless, the effects on the infection with powdery mildew were significant. As transient expression in rose petals is less effective than comparable experiments in leaves of *Nicotiana benthamiana* and given the significance of the functional effects in the infection assays in both VIGS and transient RNAi experiments, in our opinion the sets of experiments strongly support the function of both genes in the rose-powdery mildew interaction. The role of *RhMLO2* in powdery mildew susceptibility is less pronounced than the role of *RhMLO1*.

In the experiments on silencing *RhMLO1*, the line with the highest *RhMLO1* expression shows the lowest fungal biomass (Fig. 4b, c). Such an experimental outcome in a VIGS/pathogen assay is very common and generally accepted. Even by using a stable transgenic RNAi line of rose RhMLO1, Qiu et al. (2015b) found that their RhMLO1-RNAi line 2 had the highest *RhMLO1* expression level with the lowest number of germinated conidia. The transient RNAi approach independently showed that RhMLO1 is a functional gene in powdery mildew susceptibility.

In contrast, the homologs of *MLO3/4* in these species were down-regulated or moderately up-regulated by powdery mildew (Xiang et al. 2019). In our study, silencing of *RhMLO3* and *RhMLO4*, whether performed by VIGS or by RNAi, did not change rose susceptibility to powdery mildew significantly. These results indicate that *RhMLO3/4* may possess distinct biological functions in rose, even though they are grouped in clade V with MLO orthologs proven for their S gene function to powdery mildew. Alternatively, *RhMLO3* and *RhMLO4* may have a minor contribution to powdery mildew infection (as seen in the transient silencing experiments in petals), as it is the case for *AtMLO6* and *AtMLO12*, which are additive to *AtMLO2* (Consonni et al. 2016).

An alternative to the functional down-regulation of *RhMLO1-4* by VIGS and RNAi is a CRISPR knockout of the genes (Geike et al. 2015), as this may give a more clear-cut phenotype. We have developed rose transformation systems but so far only for tetraploid roses and still with suboptimal efficiency. We have made a first attempt for genome editing of *RhMLO* genes in tetraploid rose but due to the low transformation efficiency only a few transgenics for RhMLO2 were generated, in which not all four alleles were edited (not shown). For *RHMLO3* and *-4*, finding targets which are specific for one of the genes is as impossible as it is for the RNAi approach.

### Possible biological functions of other *RhMLO* genes

RNA sequencing has been effectively used for the study of expression patterns and molecular networks in rose for petal development (Han et al. 2017) and disease resistance (Liu et al. 2018). Dubois and Sakr (2012) sampled all rose plant organs and tissues, and leaves also in response to biotic and abiotic stresses. This early study was broad in its sampling, but the sequencing depth was limited. Hence, although it is the broadest source of transcribed *MLO* genes in rose, the data are mostly relevant for presence of gene expression, not for absence. We found 11 of the 19 *RhMLO* genes in the transcriptome data of Dubois and Sakr (2012). There were two plant tissues with relatively high expression levels of *MLO* genes, namely leaves from water-stressed plants and white young roots. Especially *RhMLO3* and *RhMLO4* were highly expressed in leaves from water-stressed plants while *RhMLO1* and *RhMLO2* were not. These results further suggest that *RhMLO3* and *RhMLO4* may play a different role compared to *RhMLO1* and *RhMLO2* in rose.

Two homologs (*RhMLO17* and *RhMLO19*) clustered with the functional *MLO* susceptibility homologs from monocots in clade IV, which also contains *FvMLO17* from strawberry and *PpMLO12* from peach as well as four monocot *MLO* homologs. Whereas the S gene function for powdery mildew has been found for the monocot *MLO* genes in Clade IV, this was not so for the MLO genes in dicots (Kusch and Panstruga 2017). Further, *RhMLO17* and *RhMLO19* were not found in the transcriptome data of Old Blush.

*RhMLO9* and *RhMLO11* are in clade I, along with *AtMLO4* and *AtMLO11* which are involved in root thigmomorphogenesis. *RhMLO10* and *RhMLO12* are grouped in clade II along with *AtMLO7*, which is expressed during pollen tube growth. The available expression data for rose in ROSAseq are insufficient to be able to infer possible functions of the rose genes. Further research will be needed to link tissue-specific gene expression levels at specific developmental stages to a particular (a)biotic stimulus in a variety of different rose genotypes.

### Allelic variants of *RhMLO* genes

Almost all modern rose cultivars are tetraploid, and are presumed to have been arisen by hybridization of a number of wild species through a long breeding history (Smulders et al. 2011). The relationship among cultivated and wild material has been investigated in several studies using different molecular techniques, for instance with AFLP (Koopman et al. 2008), SSRs (Liorzou et al. 2016; Tan et al. 2017; Qi et al. 2018) and SNPs (Zhang et al. 2013; Heo et al. 2017; Yan et al. 2018). The appearance of next-generation sequencing technologies has made it cost-efficient to detect genome-wide polymorphisms (Craig et al. 2008; Smulders et al. 2019) and study the hybrid origin of tetraploid rose more thoroughly.

We identified the alleles of 19 *RhMLOs* in 22 diploid or tetraploid roses using re-sequencing data and transcriptome data, and determined the SNPs among all the alleles in the coding regions. In total, we found 198 alleles for 19 *RhMLO* genes*,* an average of 10 alleles per gene. We detected these alleles on average 1.5 times across the set of genotypes. The inferred alleles have to be confirmed by PCR amplification or cloning and sequencing, but the fact that almost half of them were found in two or more accessions or species, and a few in four accessions and species, indicates that at least part of the predictions are fairly robust. The sharing of haplotypes (e.g. allele H1 of *RhMLO1*) between diploid species and tetraploid cultivars may indicate that these diploid species or close relatives have been involved in the formation of tetraploid roses (Zhang et al. 2013). Further research is needed including more wild species to be sequenced and using long read sequencing at suitable read depth. This will likely overcome the issue of finding partial sequences which makes it difficult to align *MLO* alleles for SNPs and haplotype characterization.

Here, we have corrected sequence alignments and SNP discovery, and then defined alleles manually after aligning. Some programs and algorithms may improve the speed and veracity for data analysis if more genes and accessions are included, such as QualitySNPng (Nijveen et al. 2013) and SAMtools. Indels have been found in some alleles during the procedure of manual correction. Some of these may represent errors of sequencing and assembling, but genetic variation in the form of indels may be related to the functionality of the alleles (Appiano et al. 2015). We did not find any indels in *RhMLO1* and *RhMLO4* in coding regions. However, we did find two long deletions in *RhMLO2*. One of them was in a conserved region of the encoded protein while the other one was relevant to the C-terminal D/E-F-S/T-F tetra-peptide sequence, which is one of several motifs characteristic for *MLO* orthologs. Since *RhMLO2* is a functional gene, these two long indels could pinpoint natural mutants. In addition, two insertions in *RhMLO3* and one deletion in *RhMLO17* were found. These findings indicate possible natural loss-of-function alleles of *RhMLO* genes in clade IV or V, which may be introgressed into commercial breeding programs.

Next to existing loss-of-function alleles, new ones may be generated using random mutagenesis (e.g. with ethyl methanesulfonate, EMS) or by targeted mutagenesis with CRISPR/Cas. The latter technology is very useful for targeted mutagenesis in polyploids (Smulders et al. 2019), as all alleles of a gene or even of multiple genes can be targeted at once. As mentioned, a complication is that transformation systems for tetraploid roses still have suboptimal efficiency. Genome editing may be used to modify elite rose cultivars, but also to increase the frequency of non-functional alleles in a breeding program, so that homozygosity at *S* gene loci may be achieved without affecting the genetic diversity of other genes (Schaart et al. 2021).

## Conclusion

In this study, we have identified 19 *MLO* homologs in rose using the recently published rose genome of the diploid garden rose Old Blush. In an allelic diversity analysis of resequencing data of 22 *Rosa* accessions of eight species, 198 different alleles were observed across the 19 *RhMLO* genes. Some alleles are shared between diploid wild species and tetraploid varieties, in line with the contribution of various diploid rose species to the formation of tetraploid roses. Furthermore, VIGS and transient RNAi assays both implied that the two homologs in clade V, *RhMLO1* and likely also *RhMLO2*, are required for infection by powdery mildew. Based on this, the task ahead is to verify the function of *RhMLO2* through stable RNAi, or using gene editing. For future use, it will also be important to test them on different powdery mildew isolates. Altogether, our results provide possibilities and direction in studying *MLO* functionality in rose.

## Data access

All data are presented in the Supplementary material.

## Supplementary Information

Below is the link to the electronic supplementary material.

Supplementary file1 (RAR 194 KB)

Supplementary file2 (XLSX 53 KB)

Supplementary file3 (XLSX 65 KB)

Supplementary file4 (DOCX 1947 KB)

Supplementary file5 (DOCX 310 KB)

Supplementary file6 (TIF 17 KB)

Supplementary file7 (TIF 115 KB)

Supplementary file8 (PDF 8456 KB)

Supplementary file9 (PDF 8451 KB)

Supplementary file10 (PDF 8541 KB)

Supplementary file11 (PDF 8427 KB)

Supplementary file12 (PDF 8412 KB)

Supplementary file13 (DOCX 17 KB)

Supplementary file14 (DOCX 103 KB)

Supplementary file15 (DOCX 23 KB)

Supplementary file16 (DOCX 43 KB)

Supplementary file17 (DOCX 14 KB)
