# Supplementary material for: Analysis of allelic variants of RhMLO genes in rose and functional studies on susceptibility to powdery mildew related to clade V homologs
Source: Theor Appl Genet. 2021 May 2;134(8):2495–515. doi: 10.1007/s00122-021-03838-7 (PMC8277636; doi:10.1007/s00122-021-03838-7)

**Supplementary Figure S1. Multiple amino acid sequence alignment of *RhMLO*s.** The alignment was generated by Clustalx1.83 using default parameters. Dark blue boxes indicate identical amino acids and pink or blue boxes indicated similar amino acids. Seven transmembrane domains (TM) are underlined by black bars and the position of the calmodulin-binding site (CaMBD) is indicated by a gray bar. Two conserved domains identified by Panstruga (2005) within the highly polymorphic C-termini are highlighted in numbered boxes (I and II).


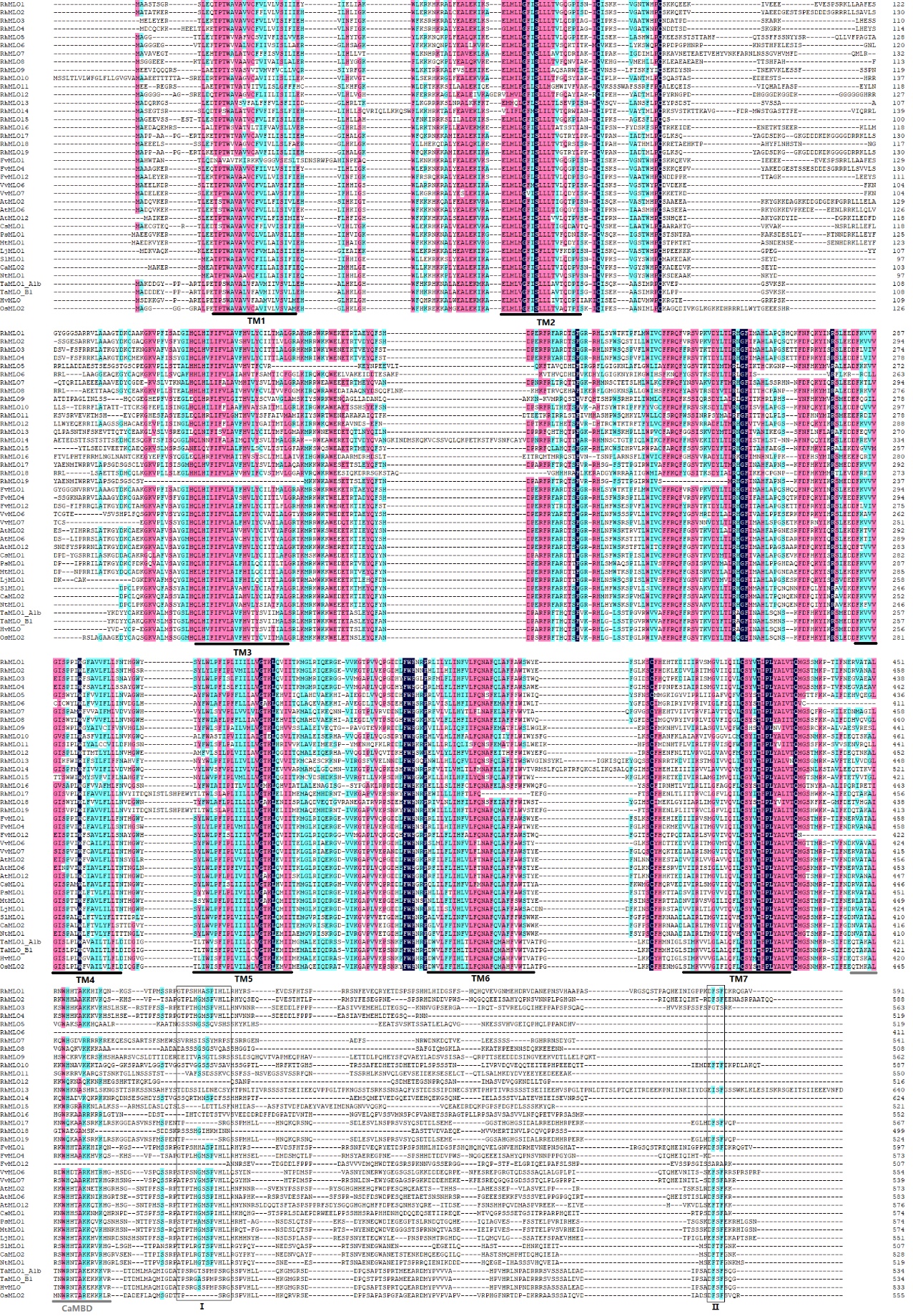

Supplement: Supplementary file 4 — Supplementary file4 (DOCX 1947 KB) [file 122_2021_3838_MOESM4_ESM.docx]
