# Supplementary material for: Analysis of allelic variants of RhMLO genes in rose and functional studies on susceptibility to powdery mildew related to clade V homologs
Source: Theor Appl Genet. 2021 May 2;134(8):2495–515. doi: 10.1007/s00122-021-03838-7 (PMC8277636; doi:10.1007/s00122-021-03838-7)

**Supplementary Figure S2. Expression profiles of transcripts encoding 19 *RhMLO genes* associated with flower** **development and scent.** The heatmap was generated based on available RPKM data from *ROSAseq* (<https://lipm-browsers.toulouse.inra.fr/plants/R.chinensis>) using the Heml analysis tool. Black boxes indicted that these genes were not found in *ROSAseq.*


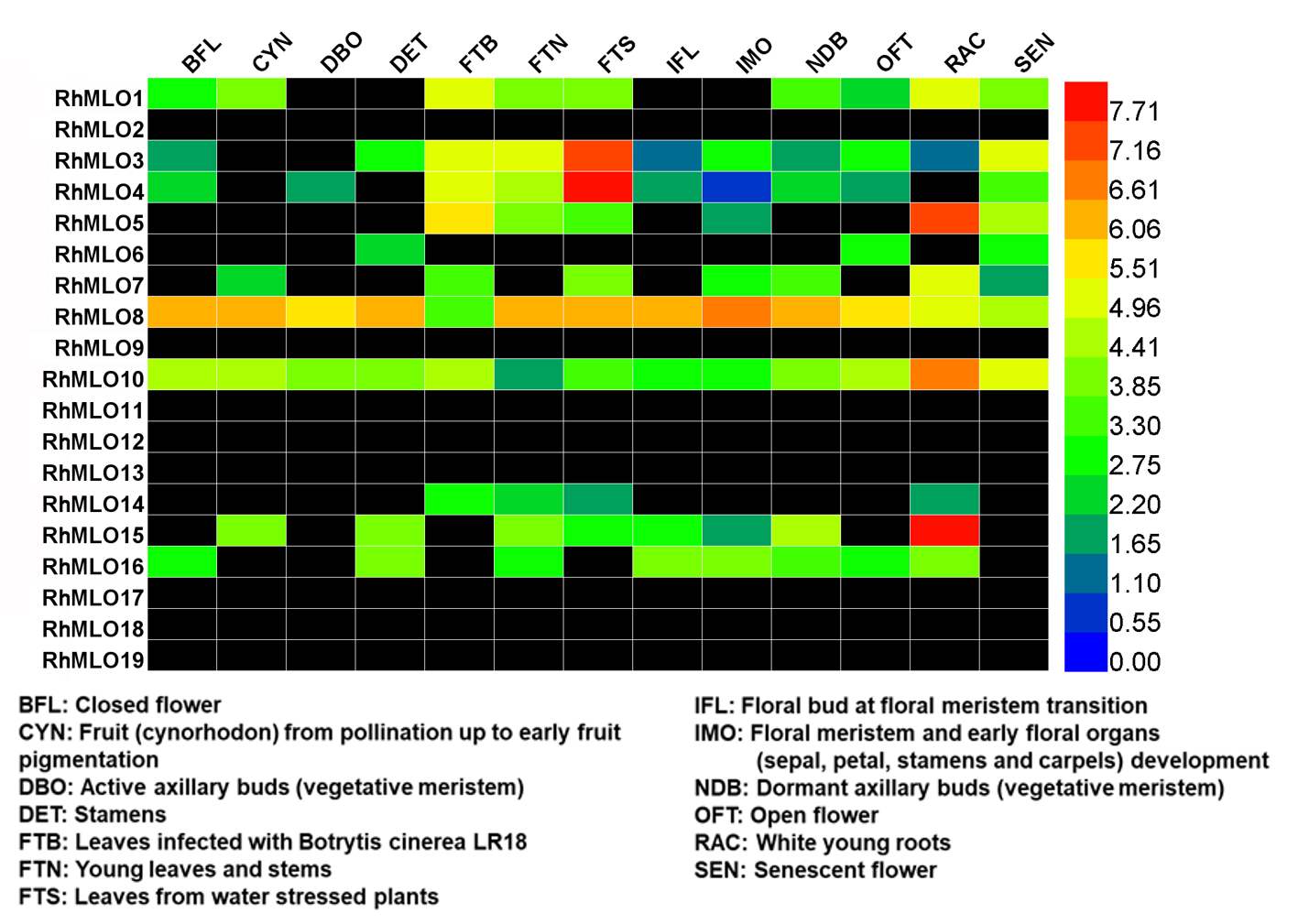

Supplement: Supplementary file 5 — Supplementary file5 (DOCX 310 KB) [file 122_2021_3838_MOESM5_ESM.docx]
