# Supplementary figures and images for: Analysis of allelic variants of RhMLO genes in rose and functional studies on susceptibility to powdery mildew related to clade V homologs
Source: Theor Appl Genet. 2021 May 2;134(8):2495–515. doi: 10.1007/s00122-021-03838-7 (PMC8277636; doi:10.1007/s00122-021-03838-7)

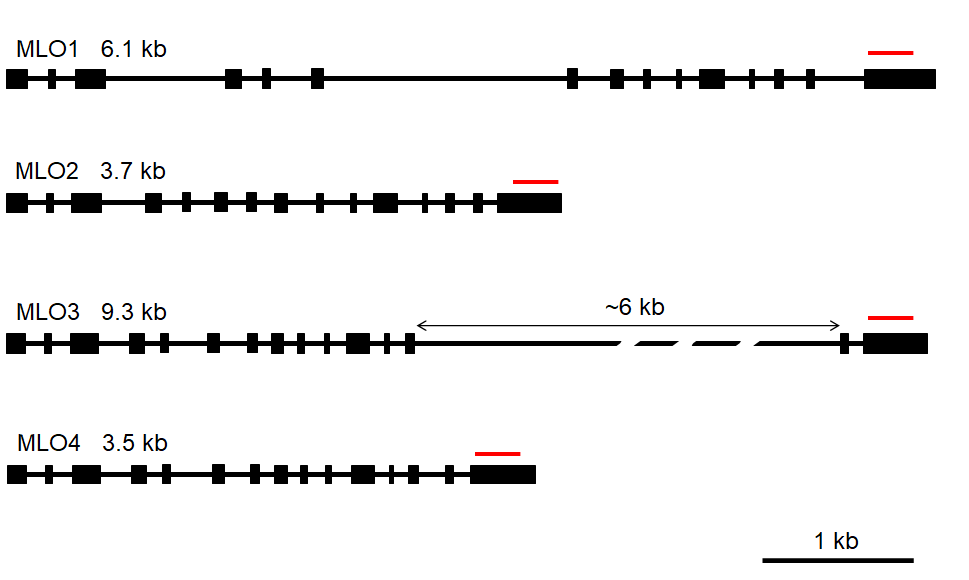

Supplement: Supplementary file 6 — Supplementary file6 (TIF 17 KB) [file 122_2021_3838_MOESM6_ESM.tif]

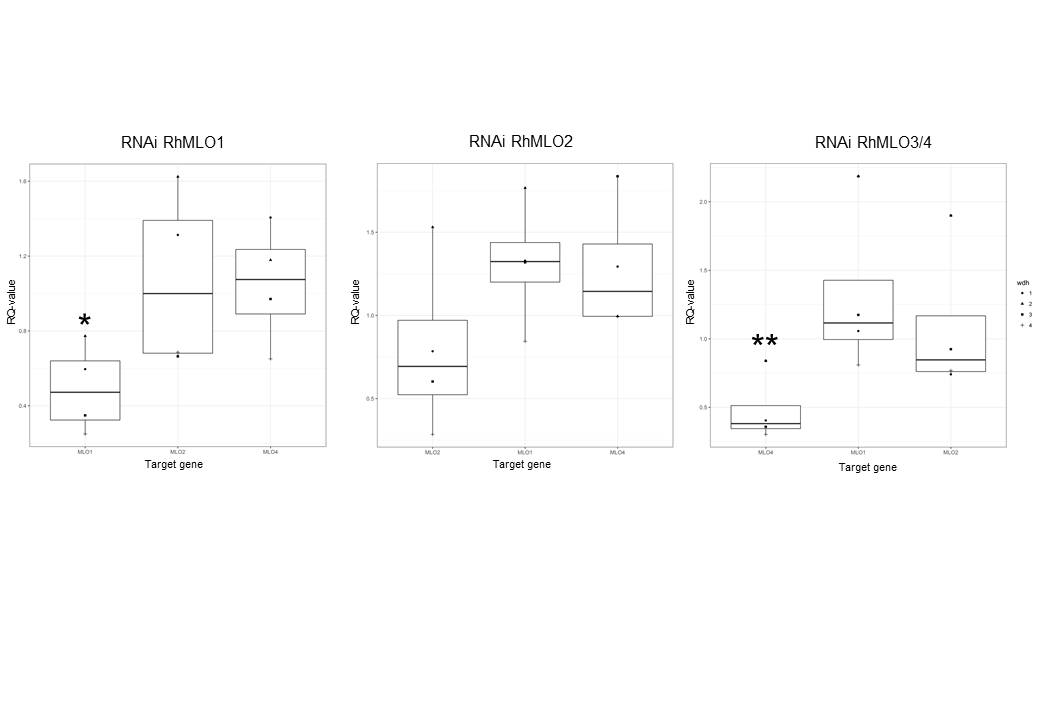

Supplement: Supplementary file 7 — Supplementary file7 (TIF 115 KB) [file 122_2021_3838_MOESM7_ESM.tif]

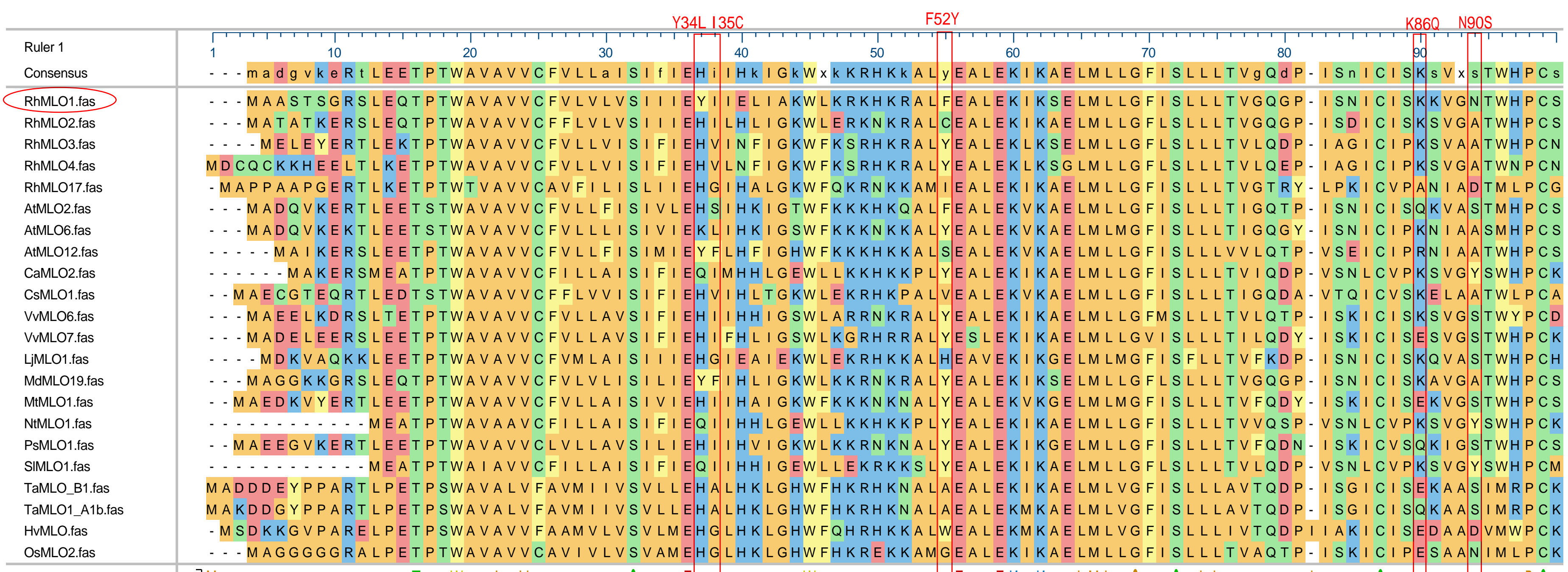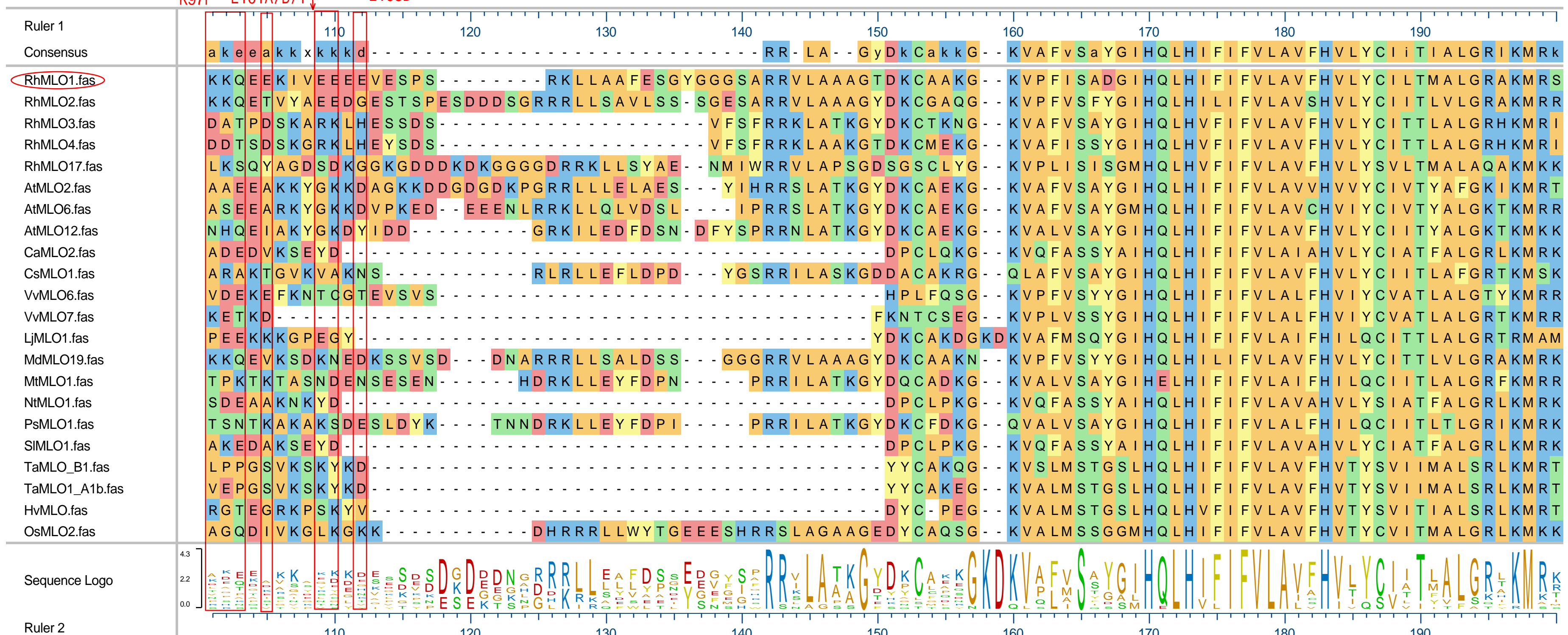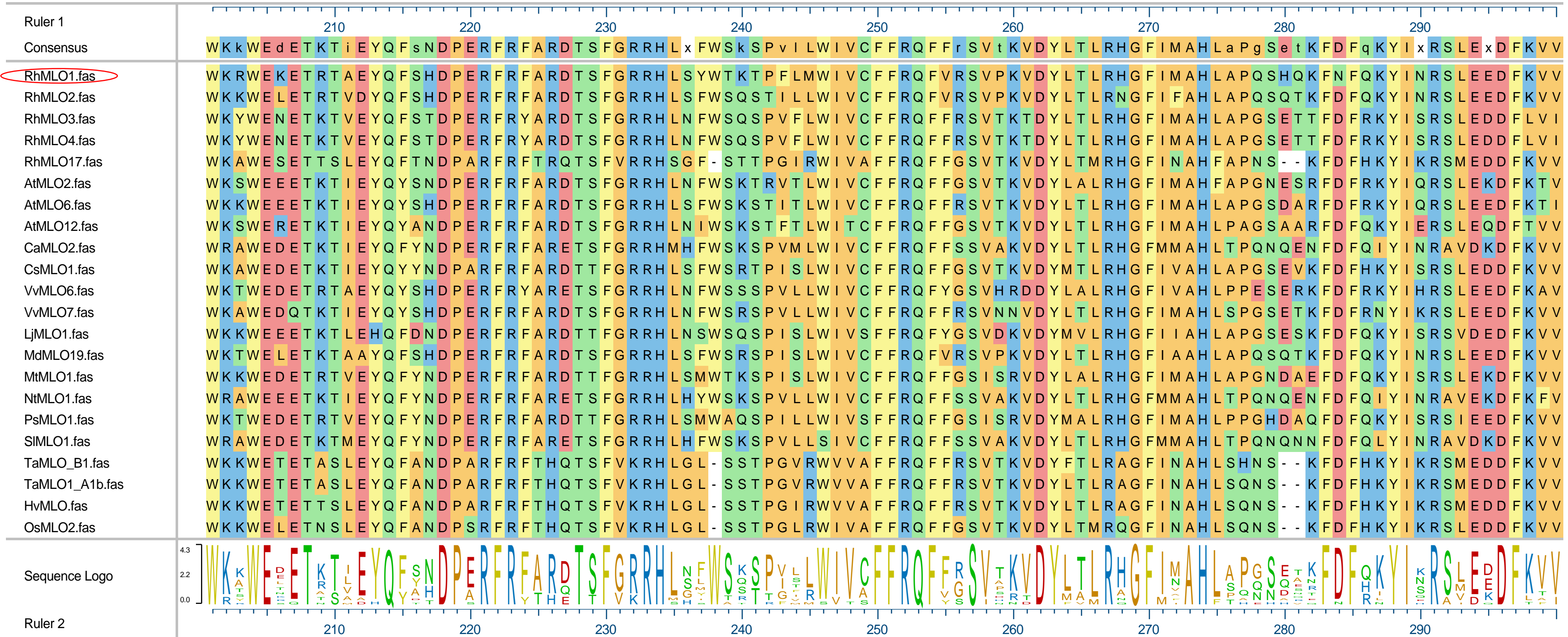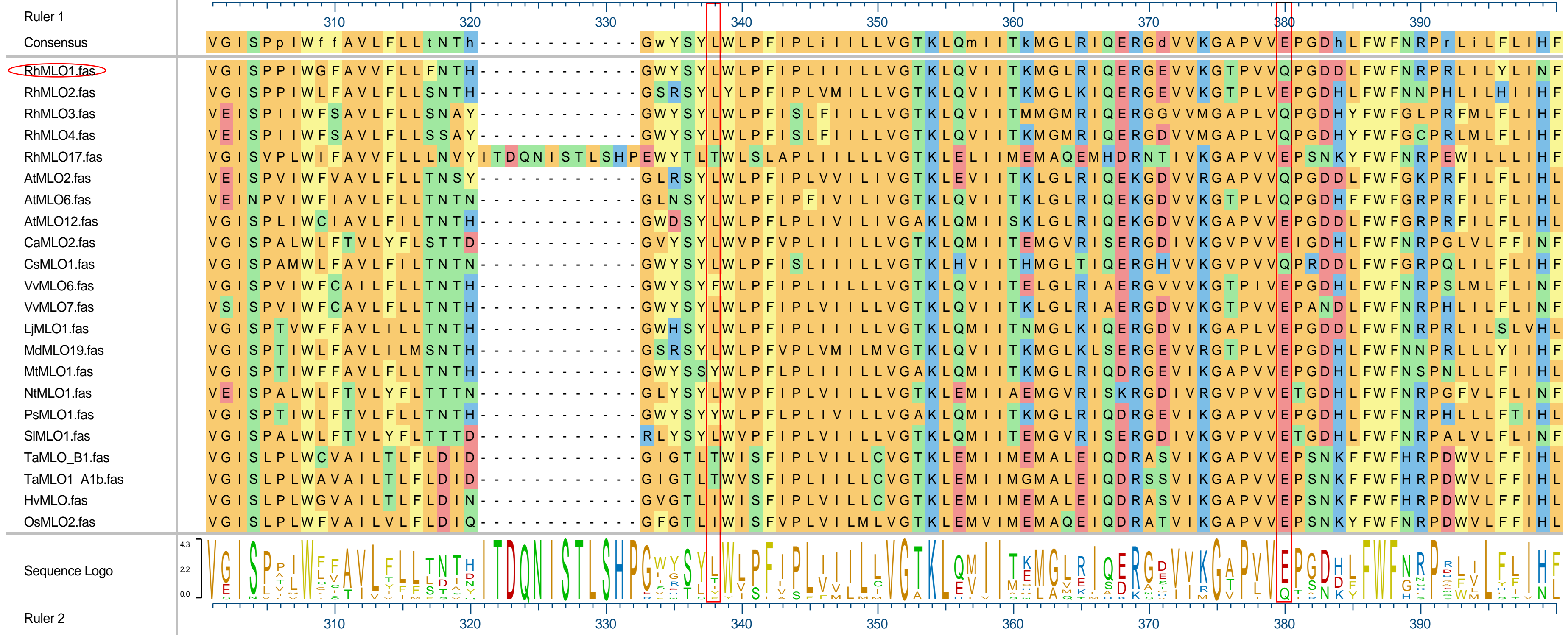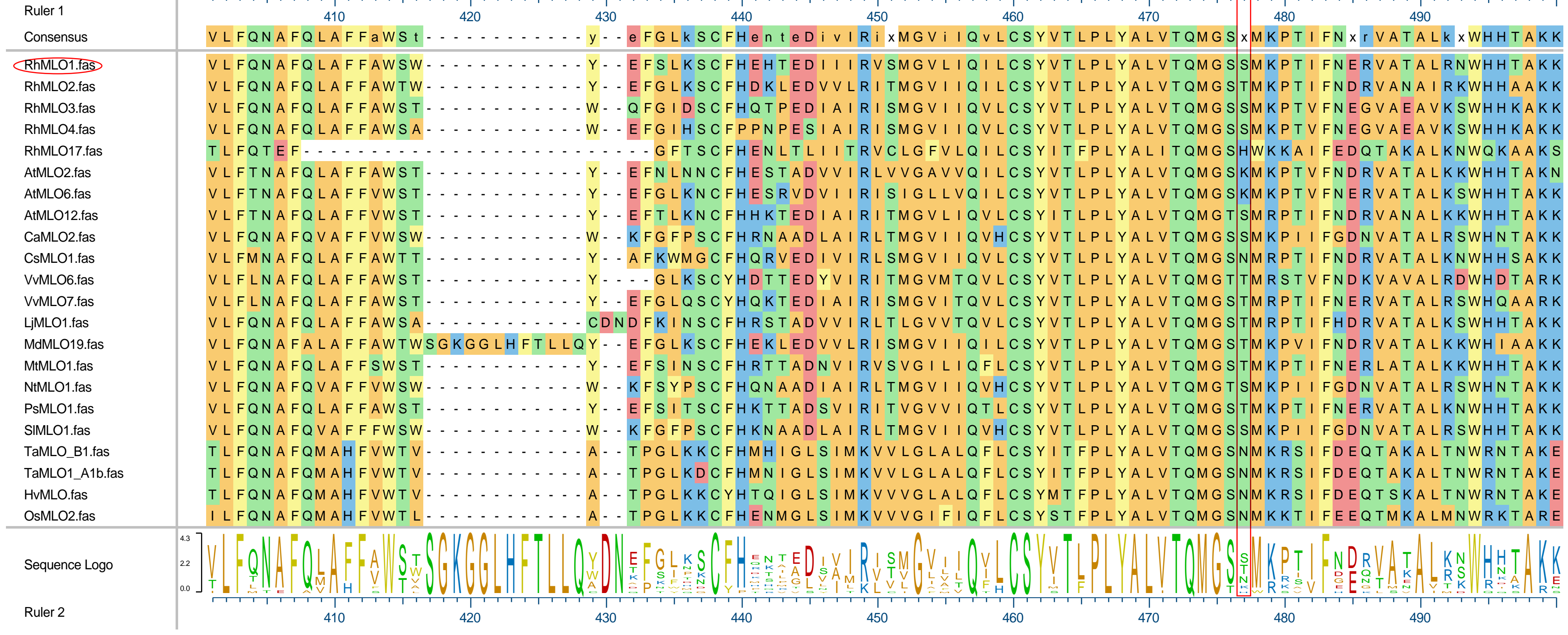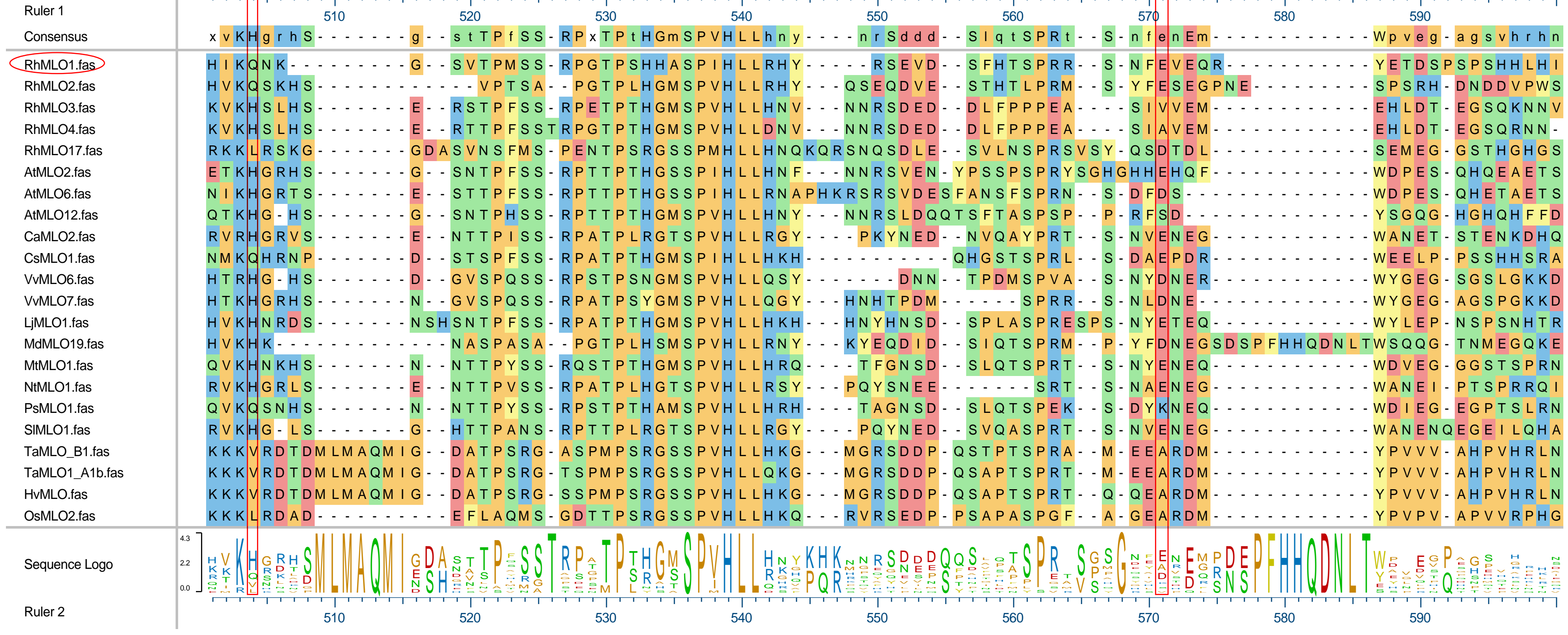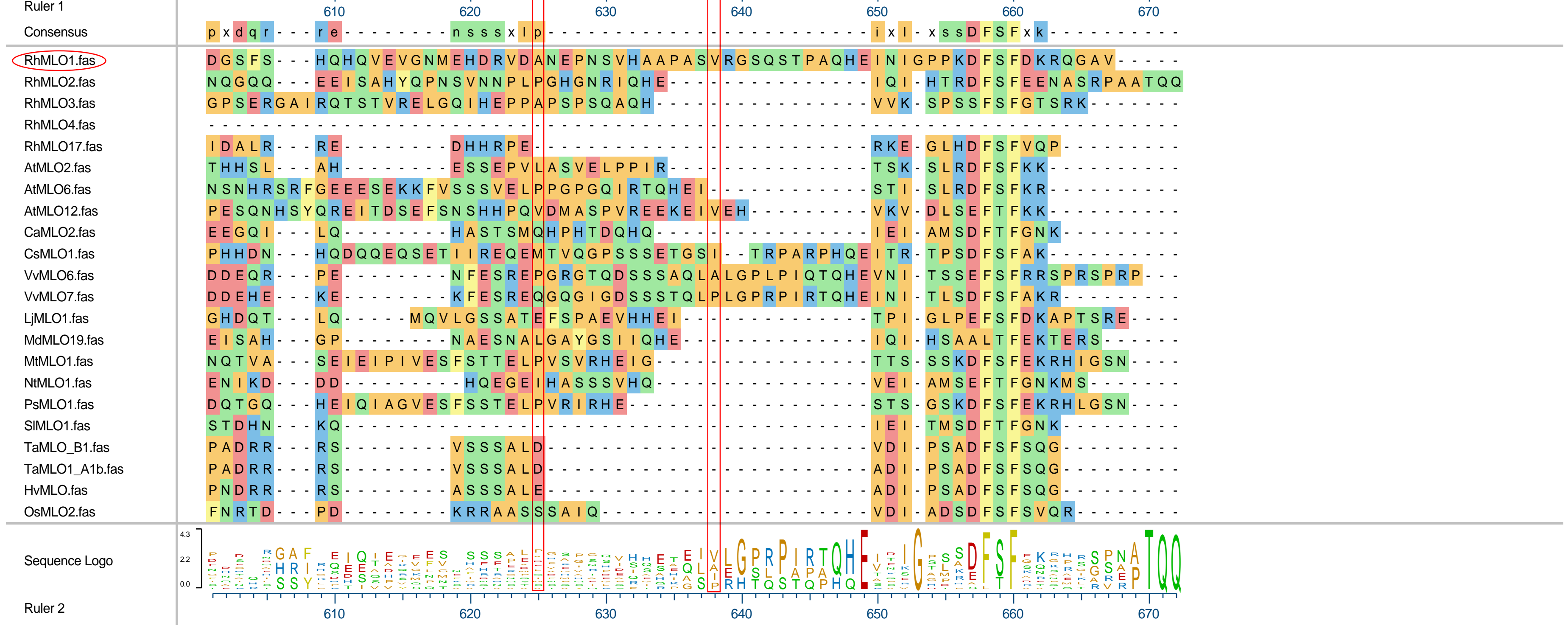

Supplement: Supplementary file 8 — Supplementary file8 (PDF 8456 KB) [file 122_2021_3838_MOESM8_ESM.pdf]

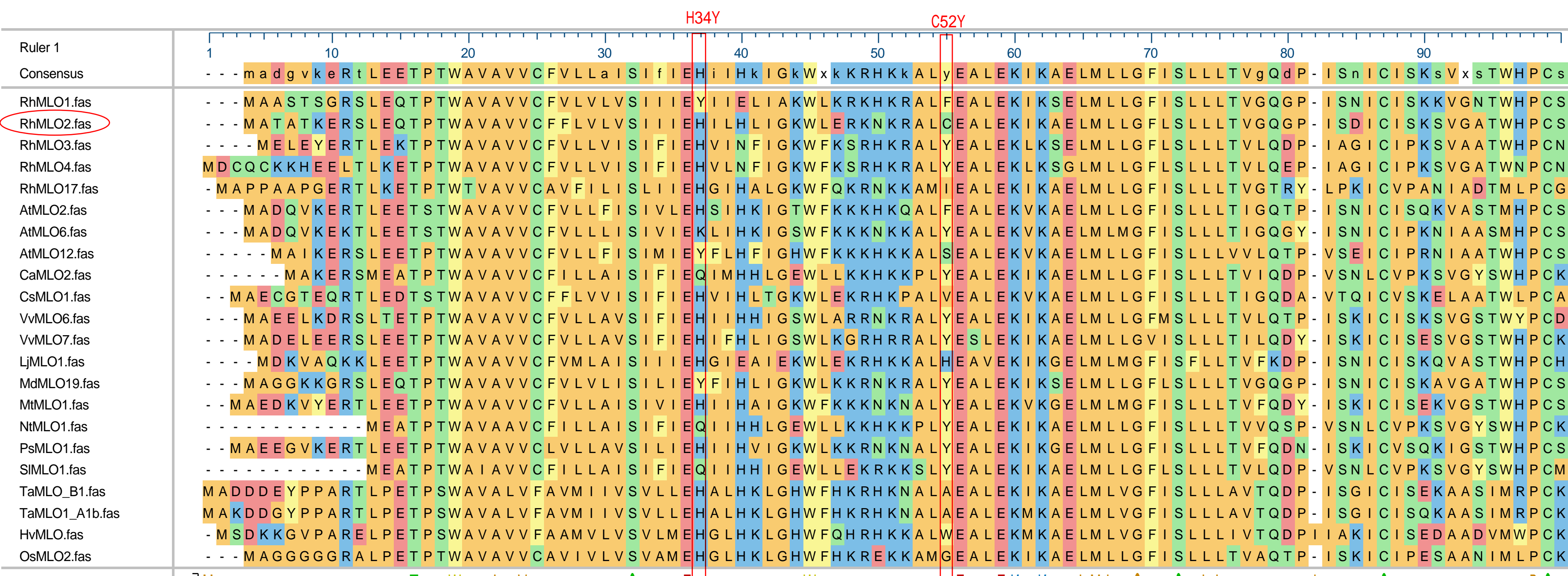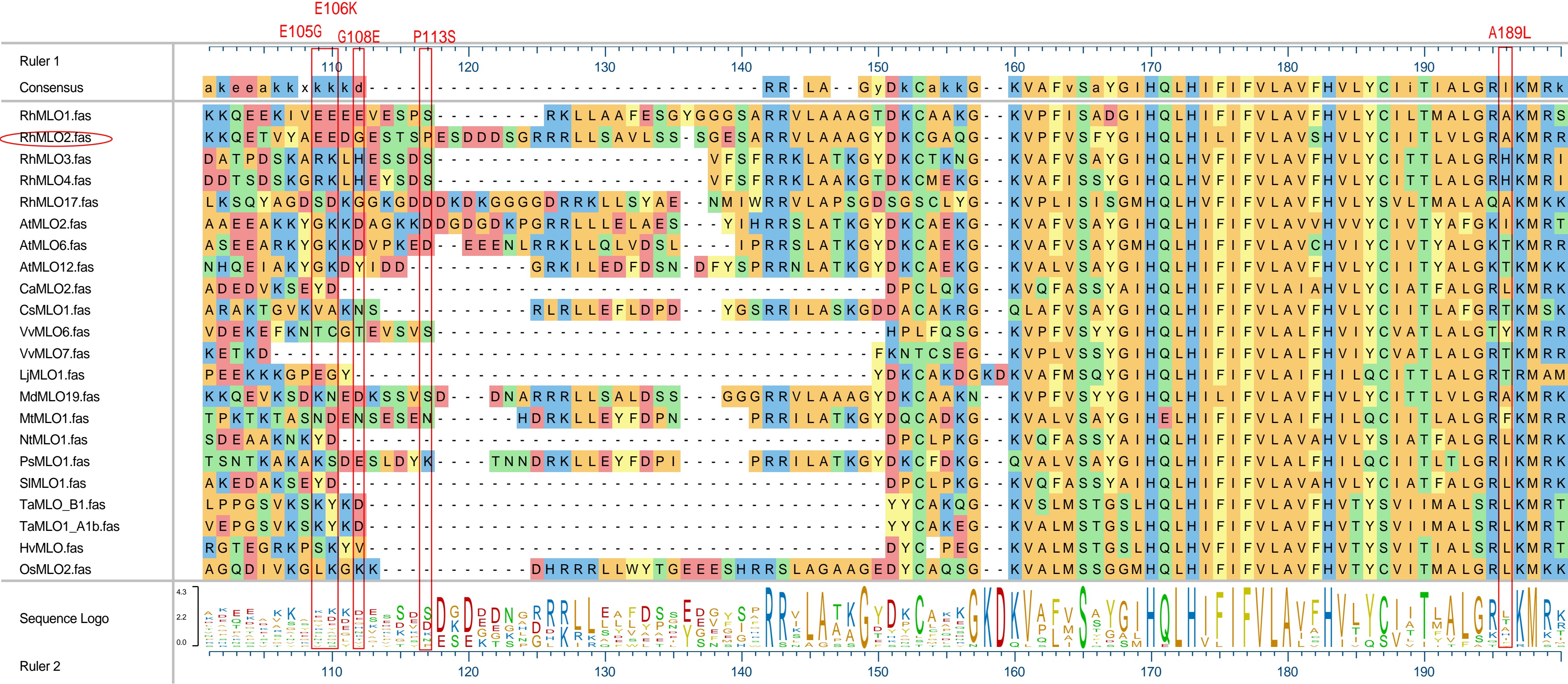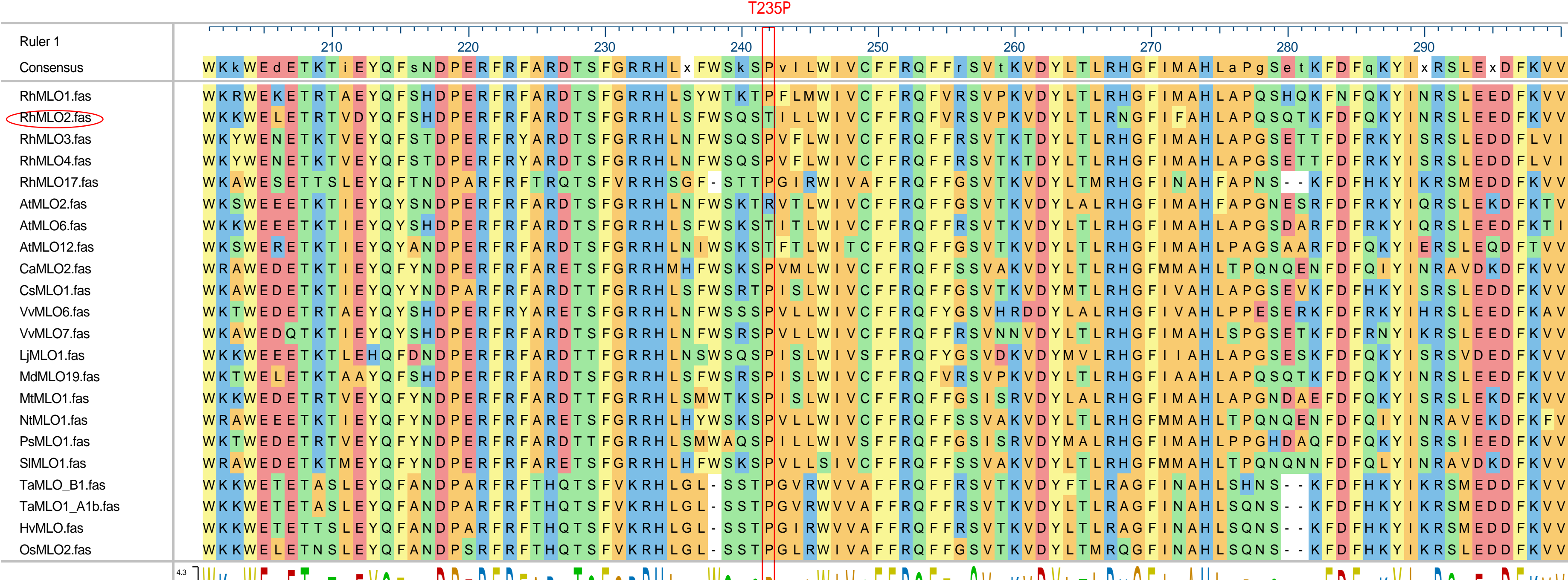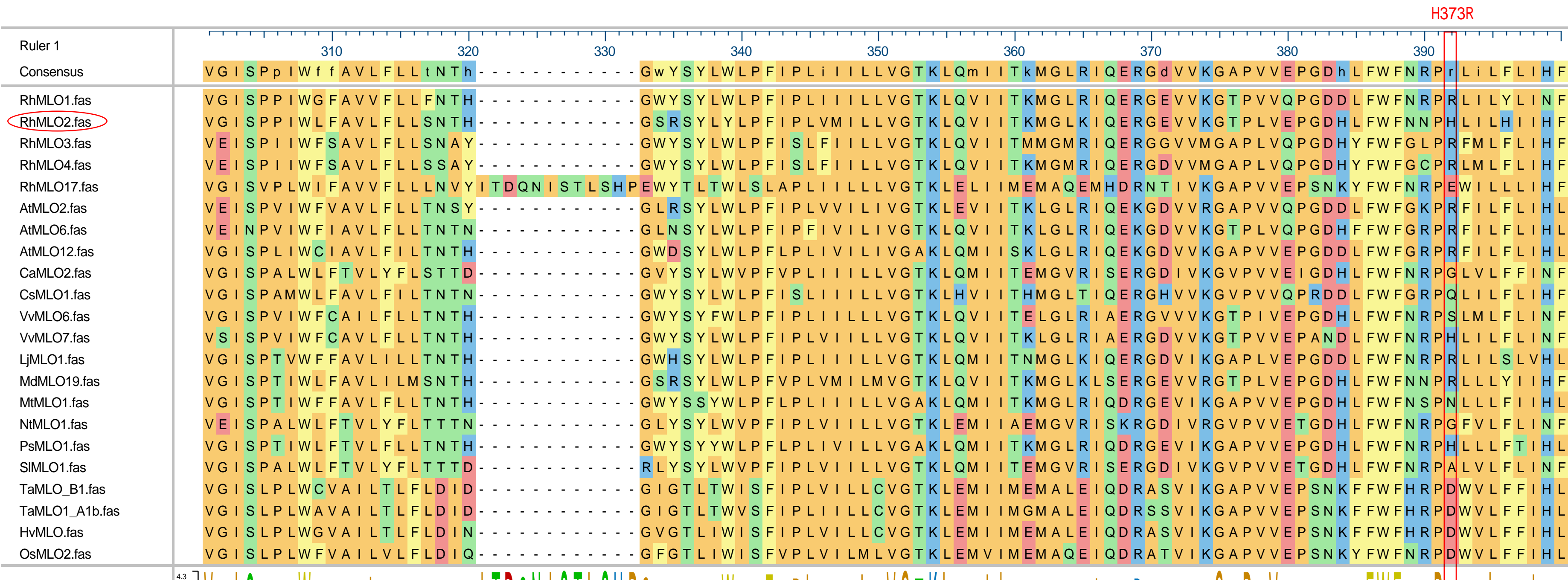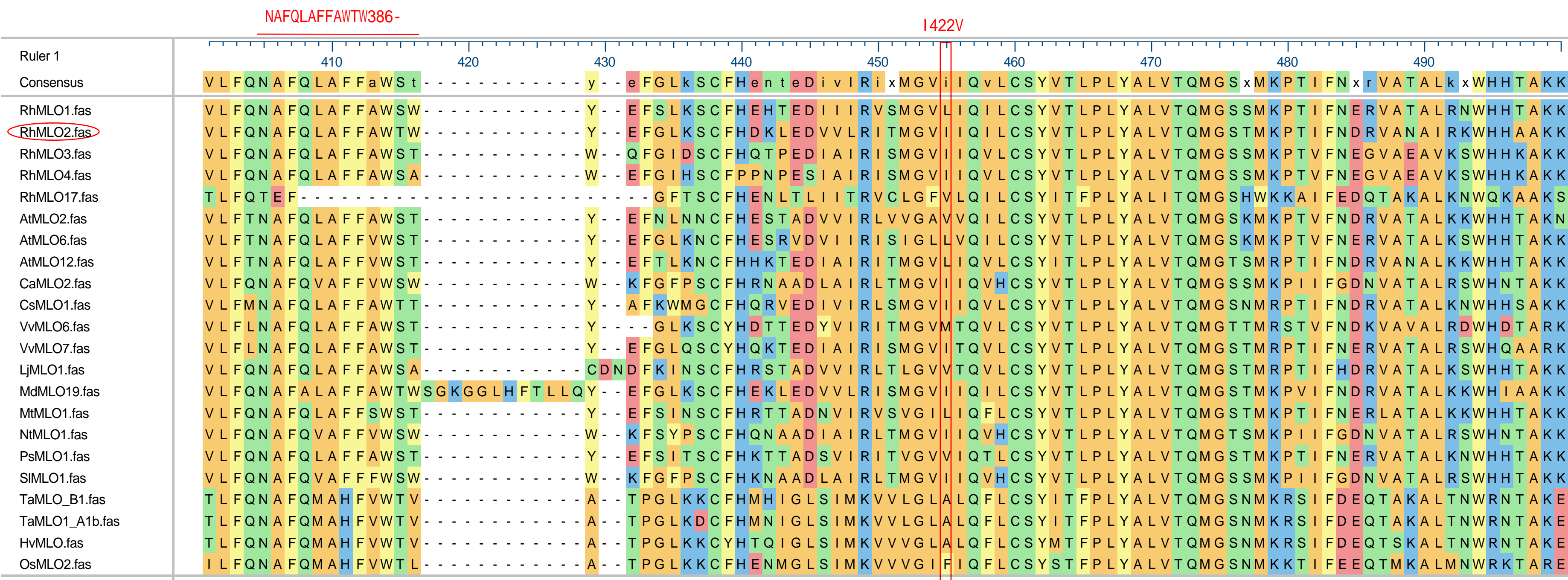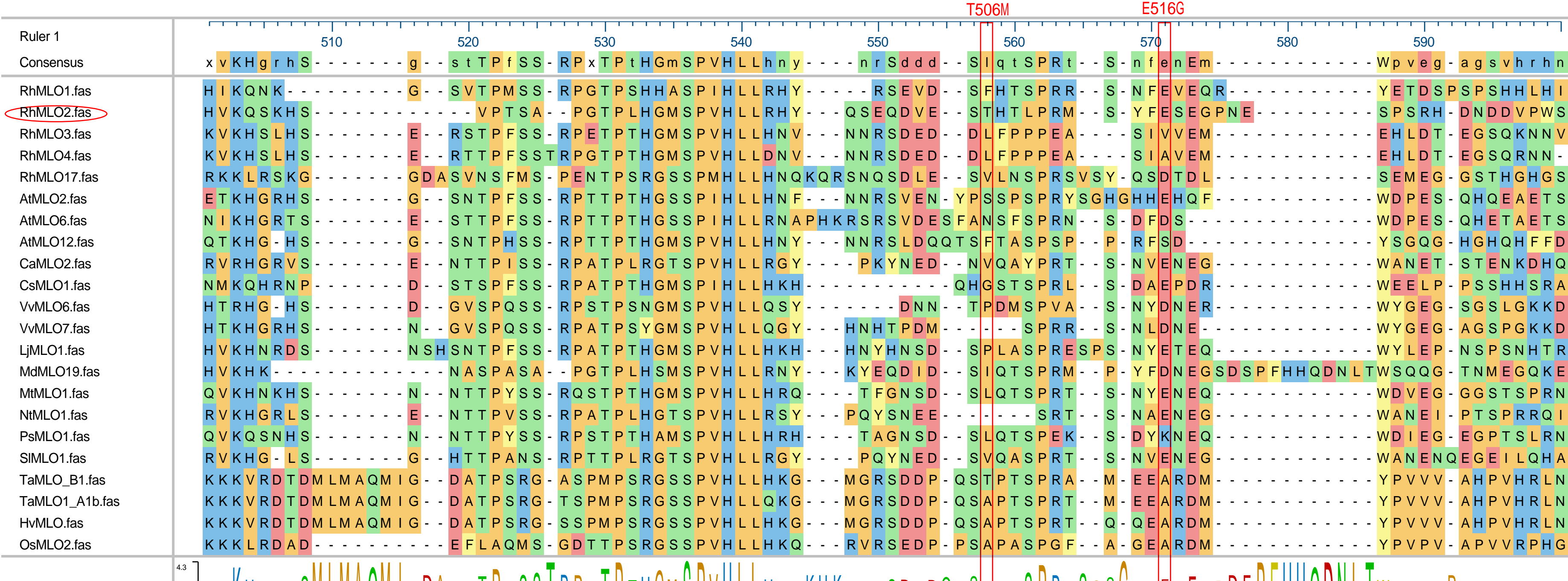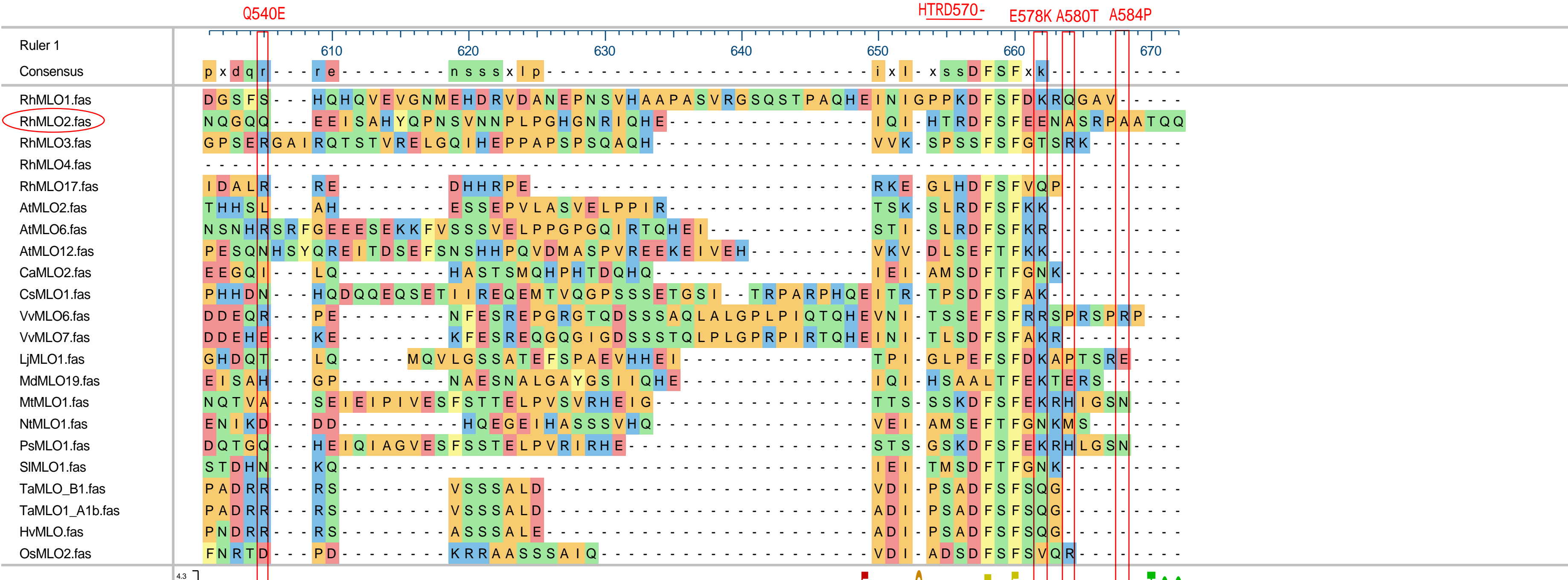

Supplement: Supplementary file 9 — Supplementary file9 (PDF 8451 KB) [file 122_2021_3838_MOESM9_ESM.pdf]

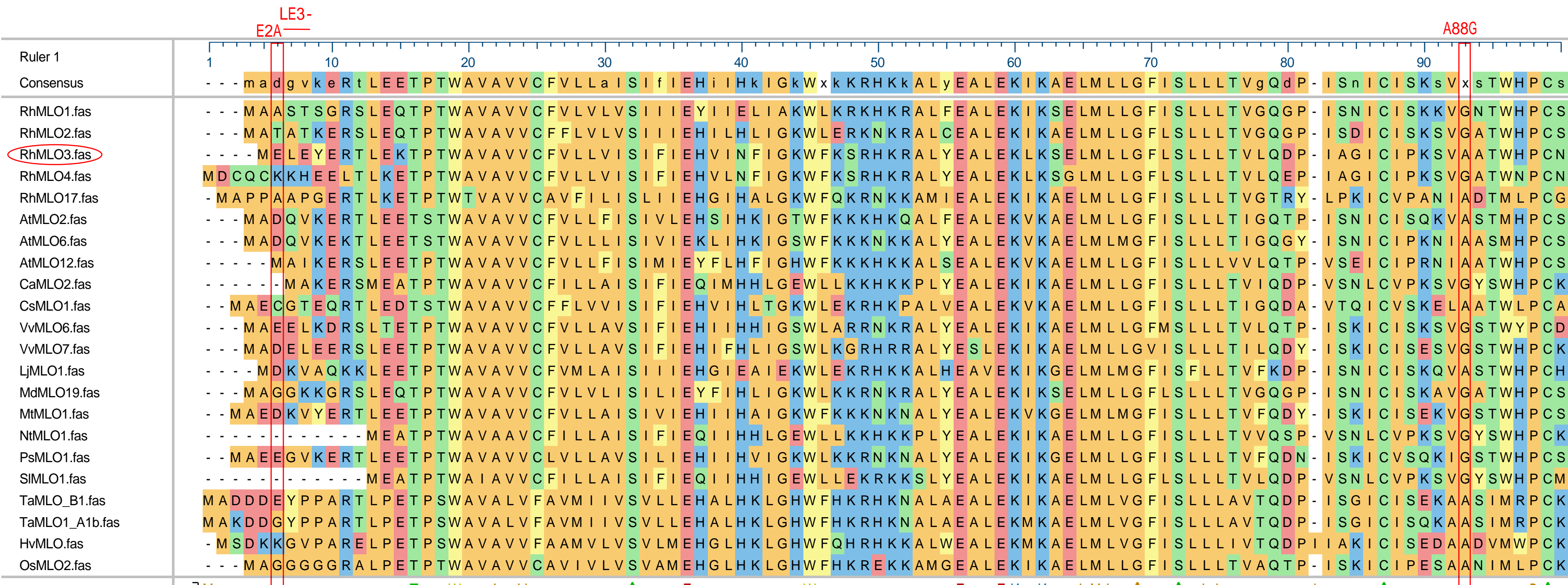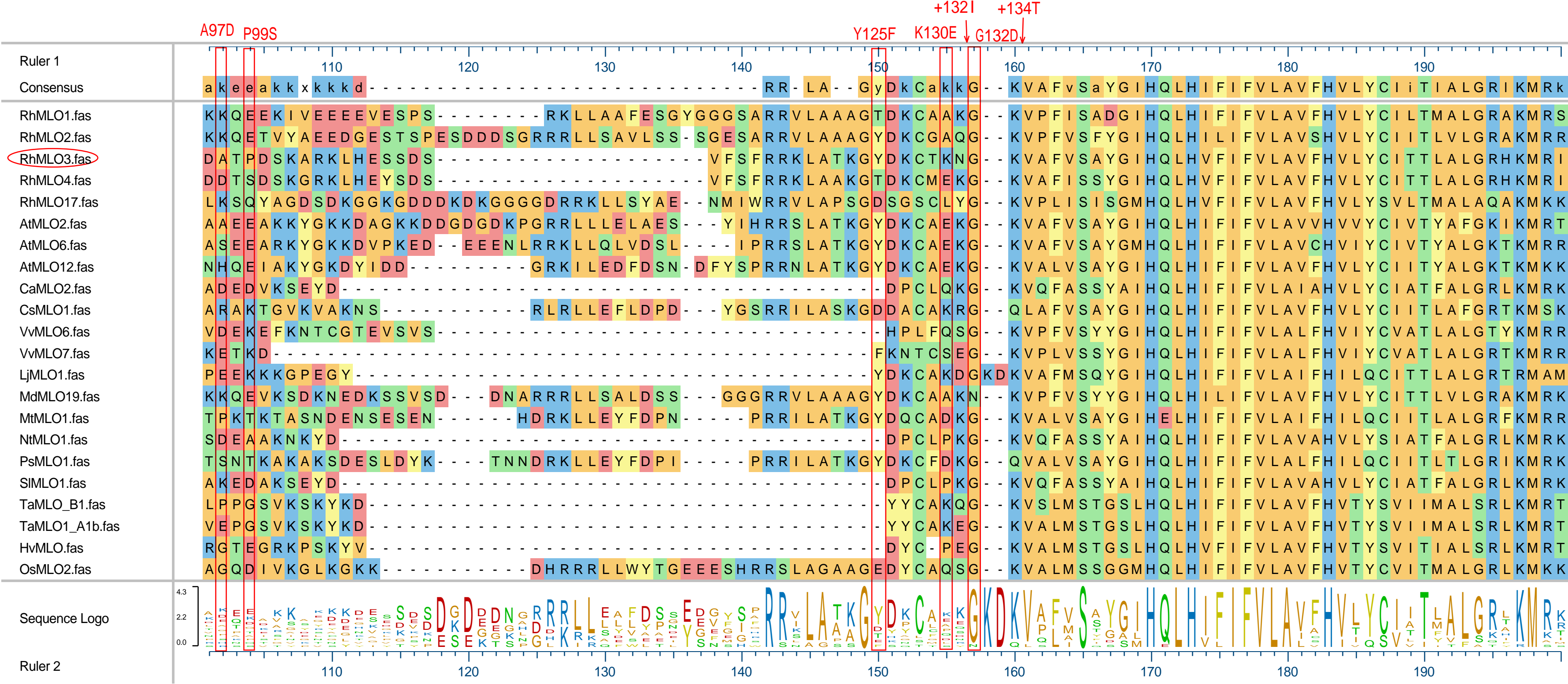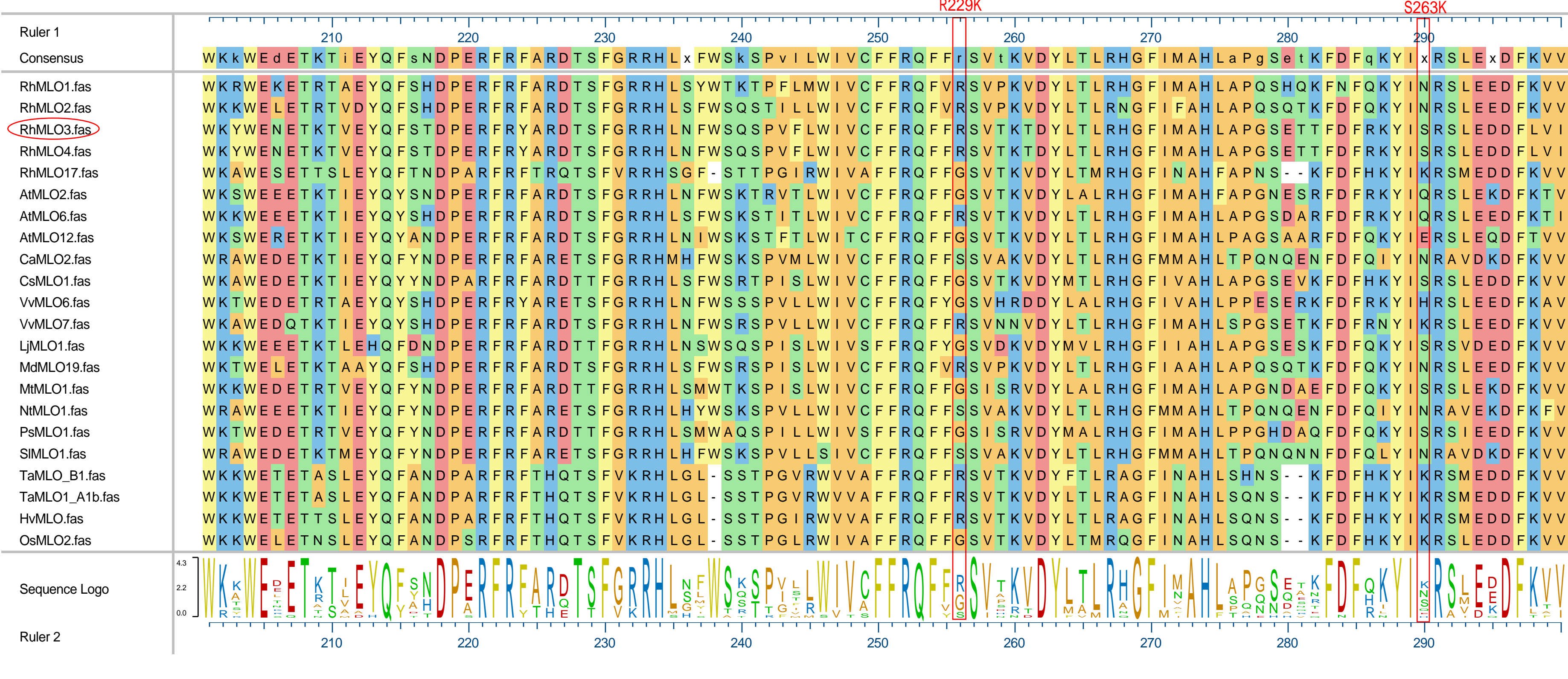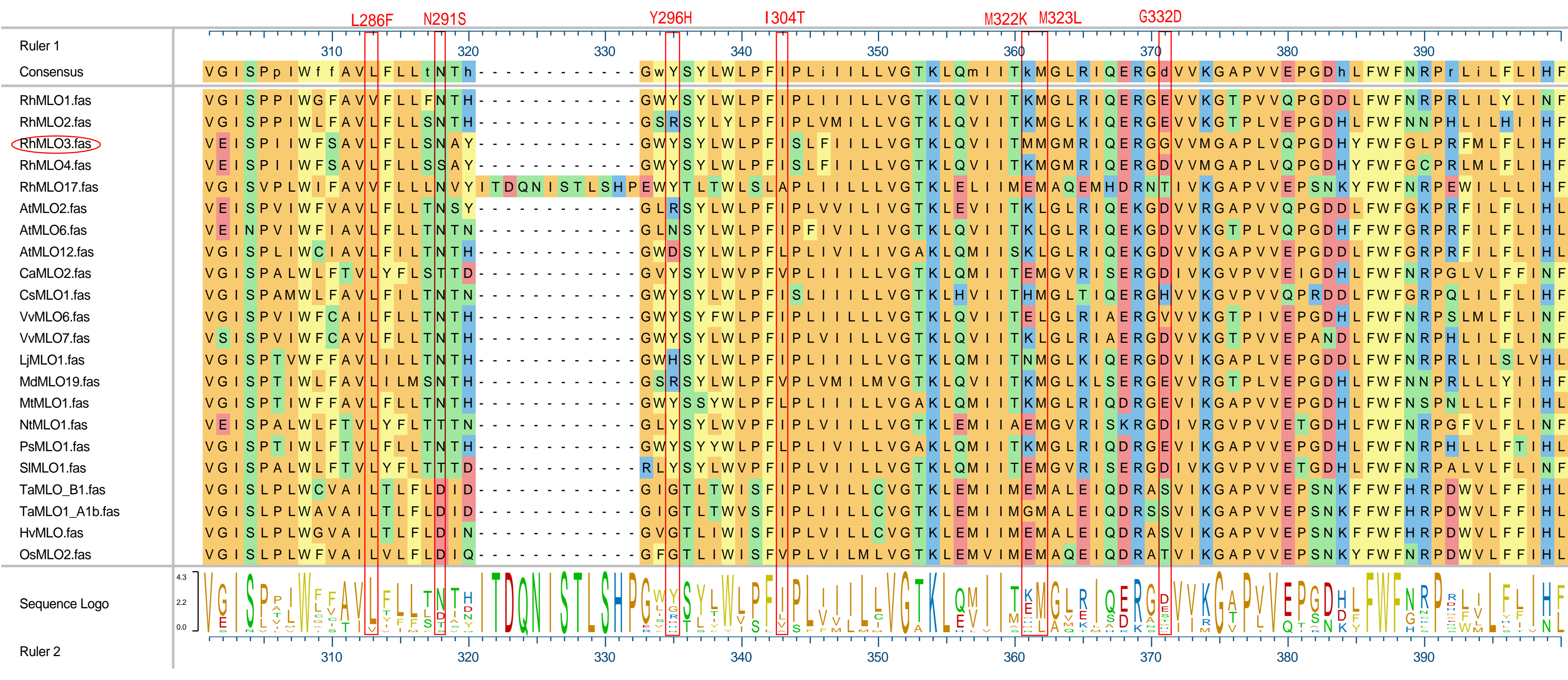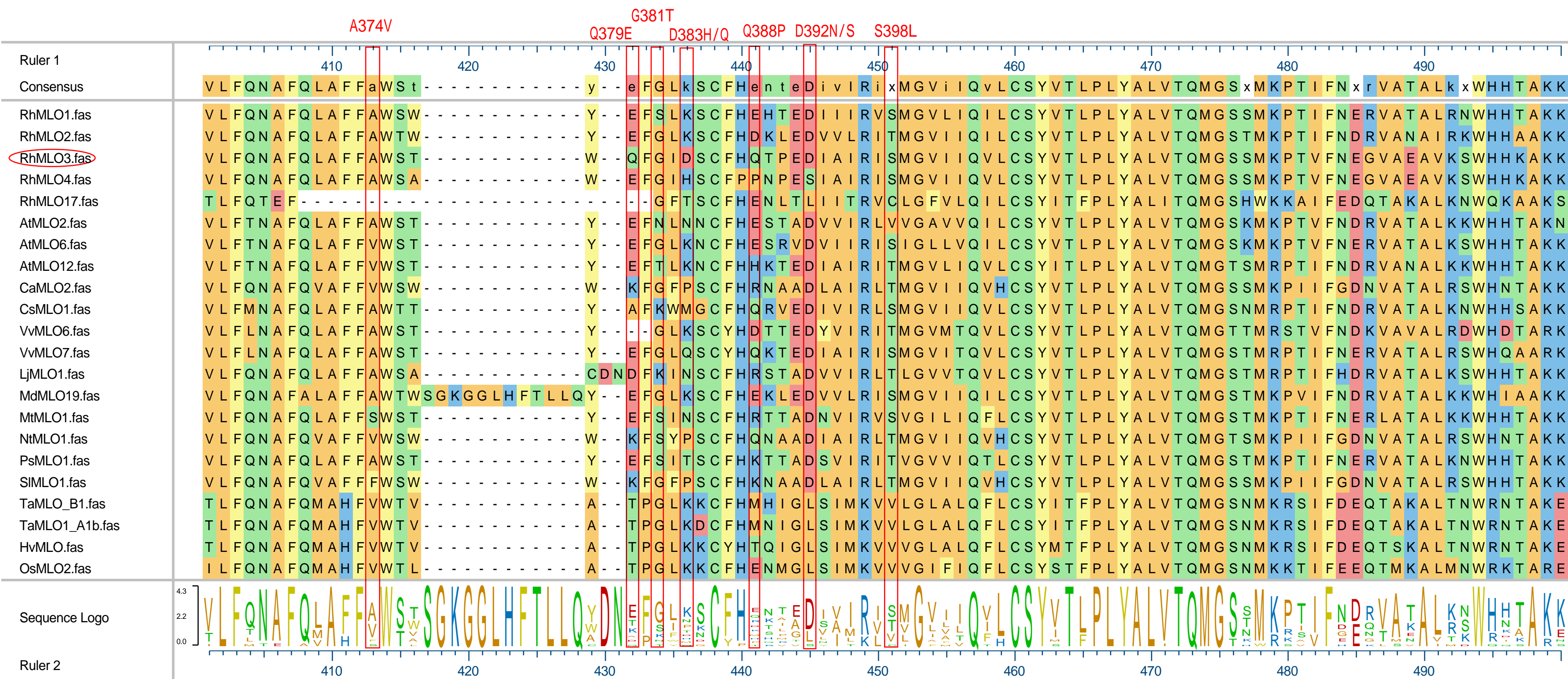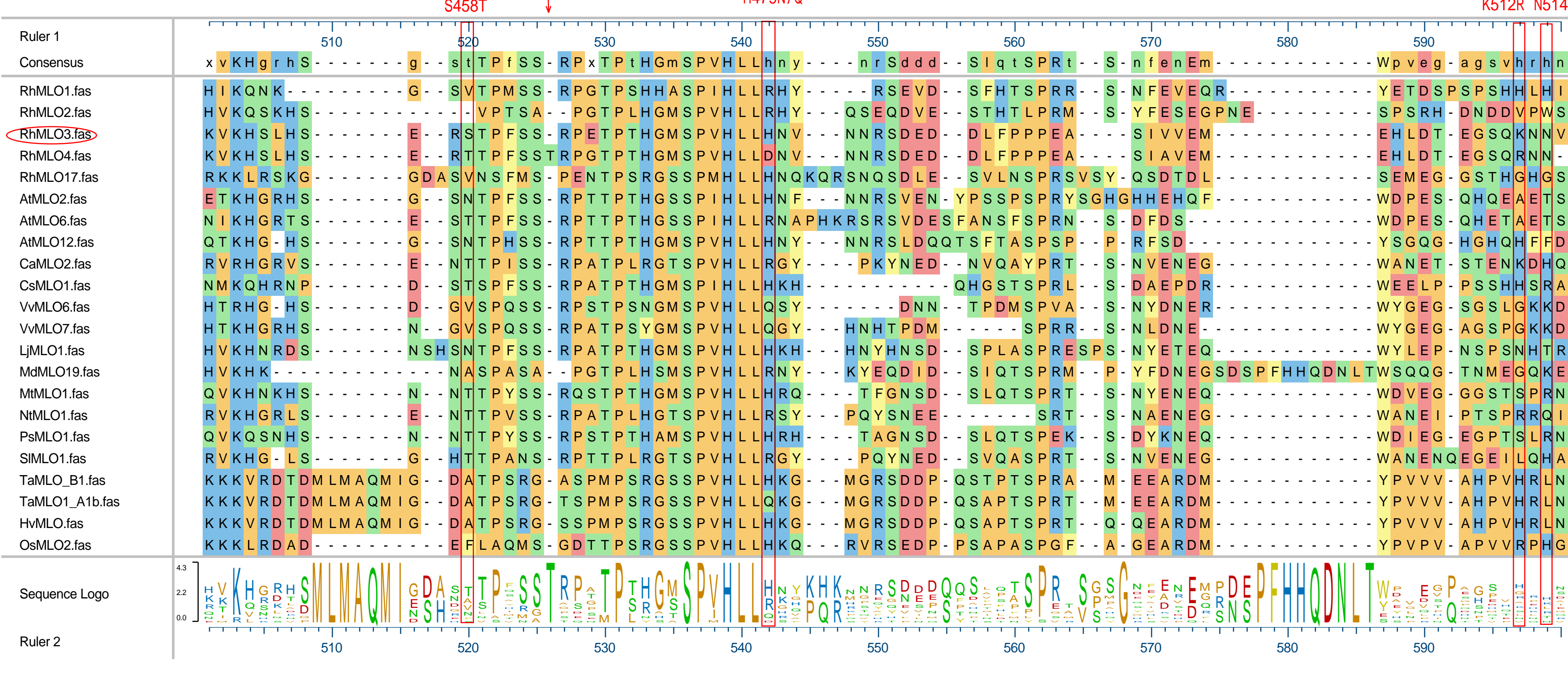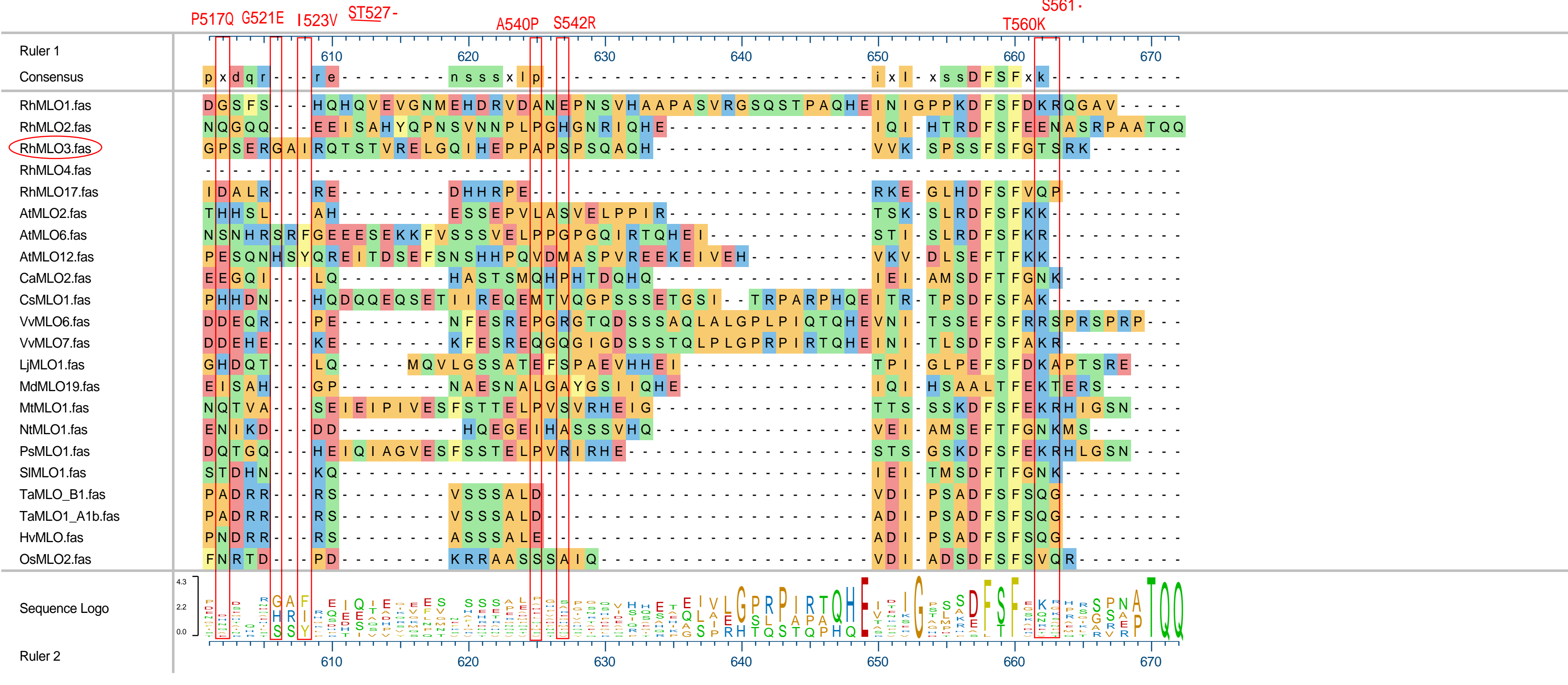

Supplement: Supplementary file 10 — Supplementary file10 (PDF 8541 KB) [file 122_2021_3838_MOESM10_ESM.pdf]

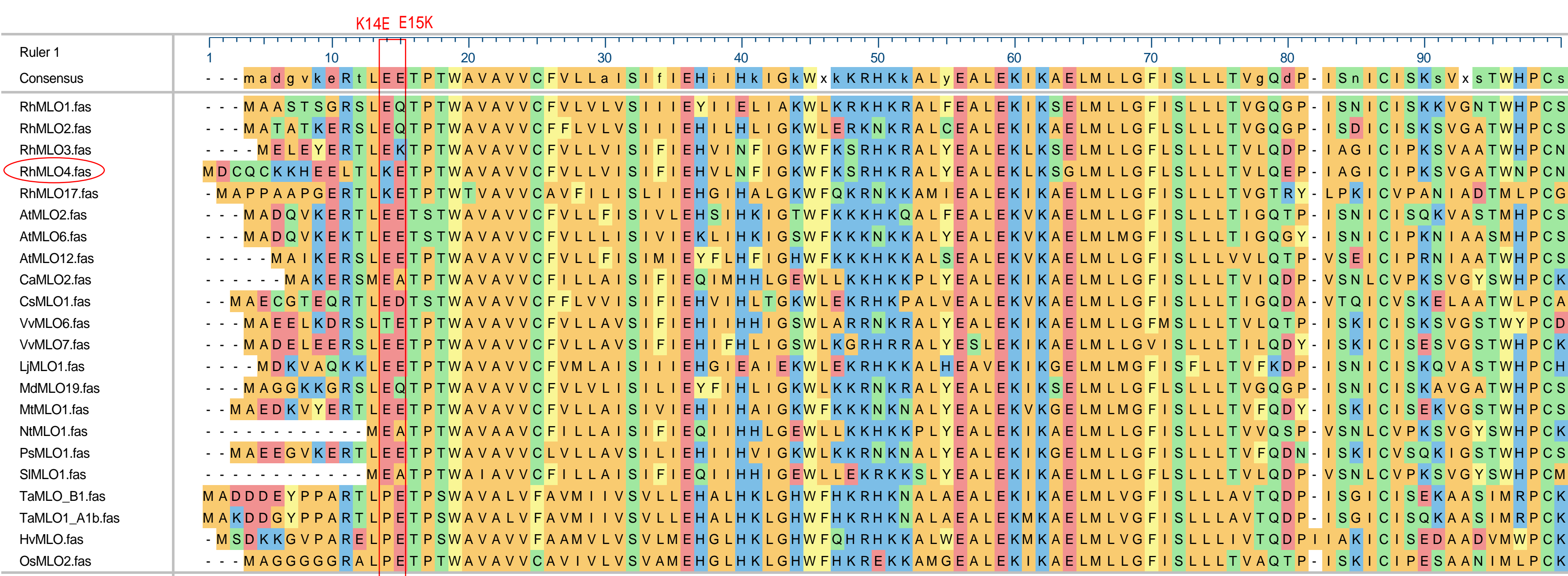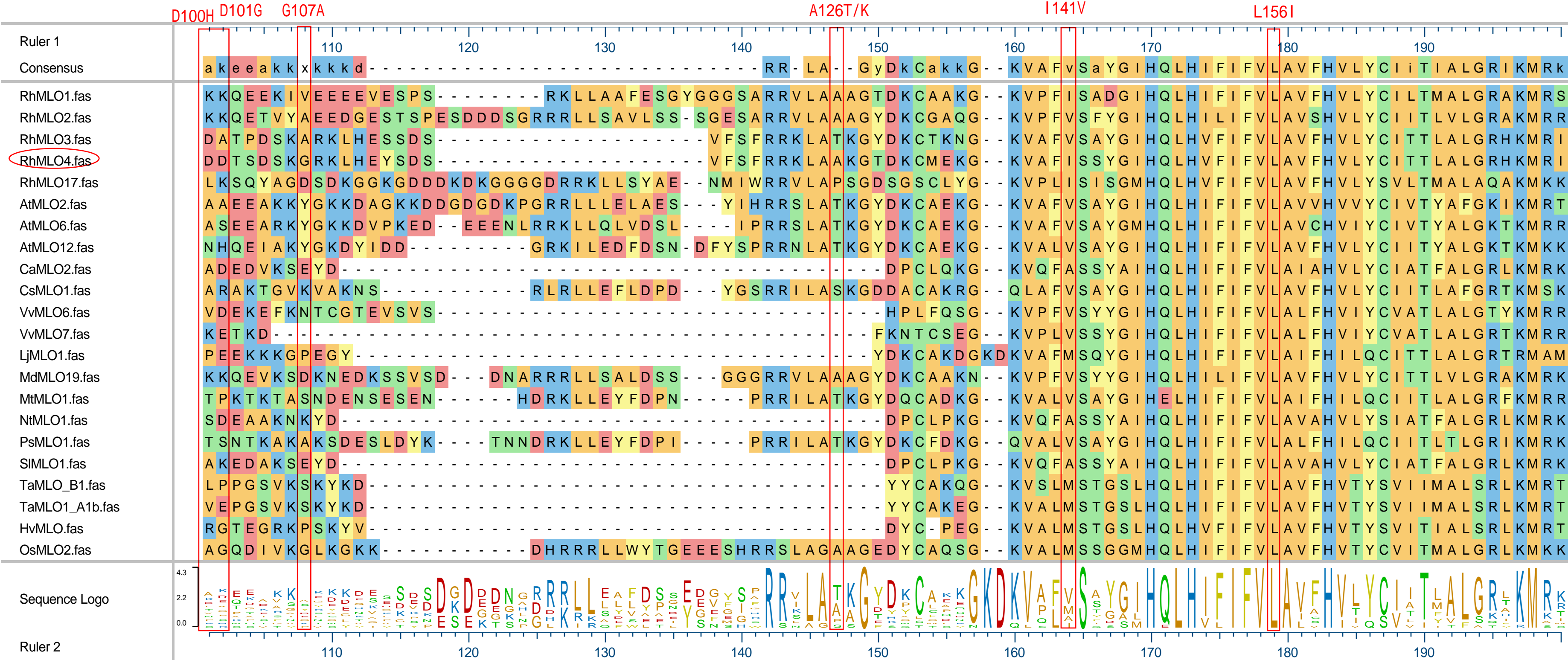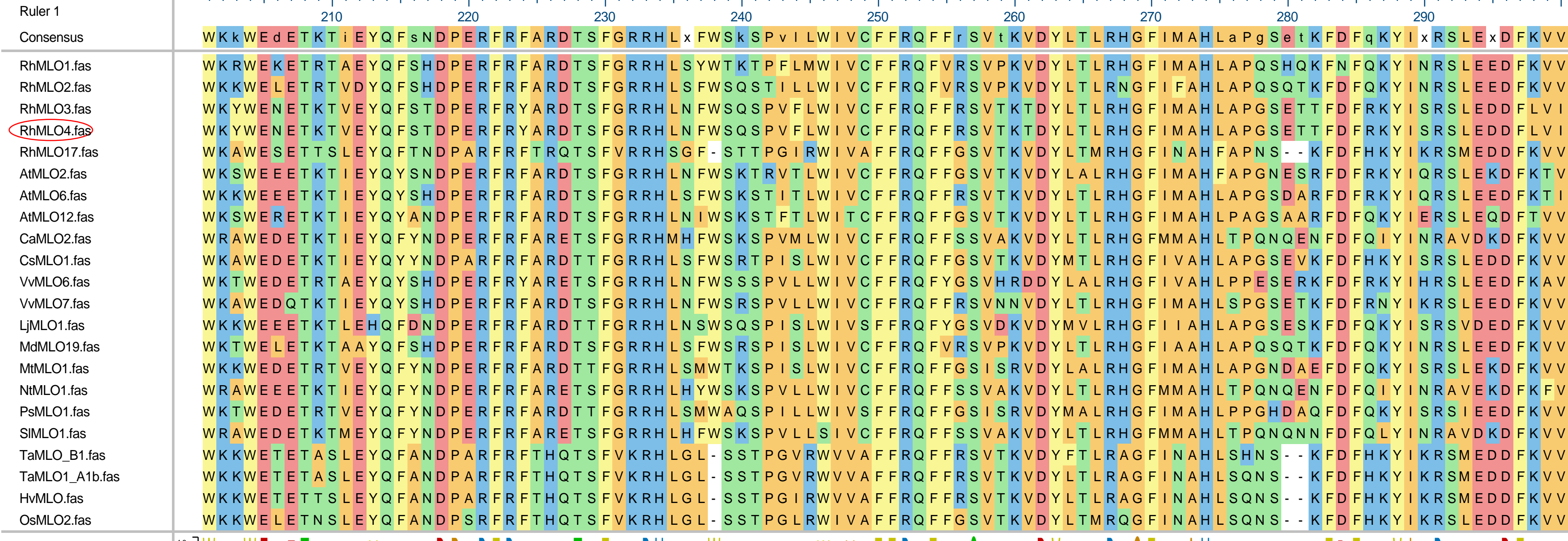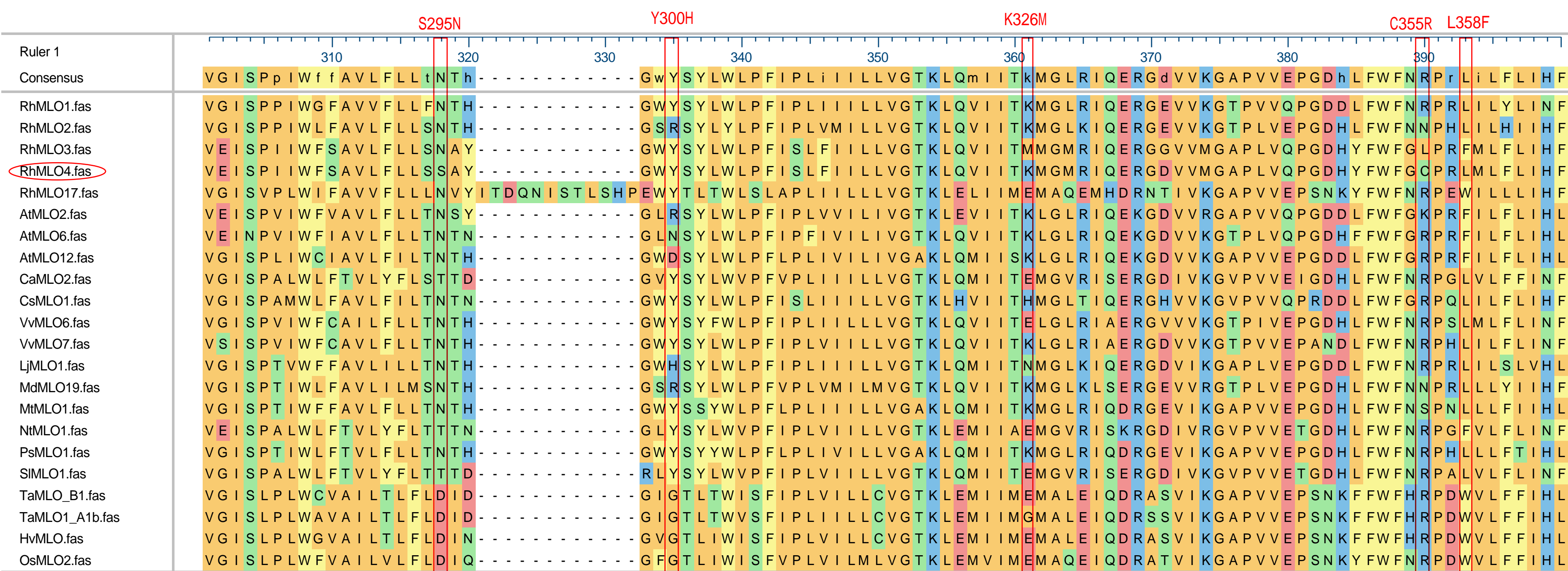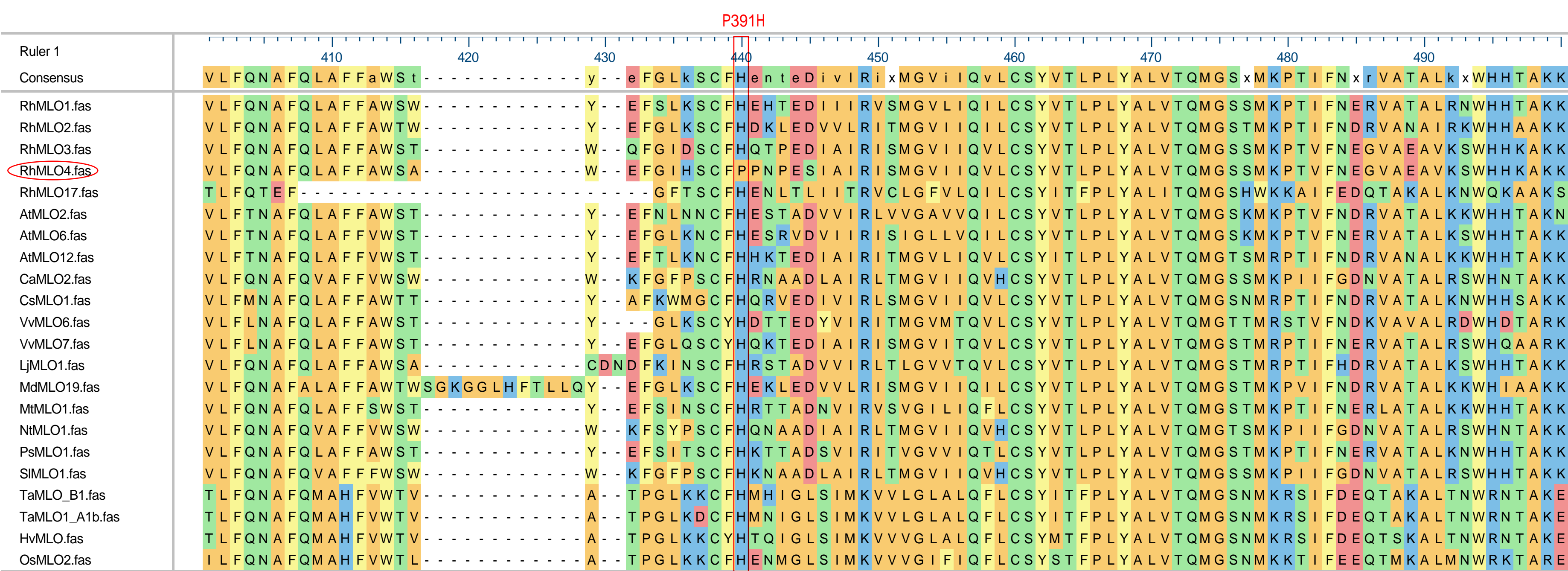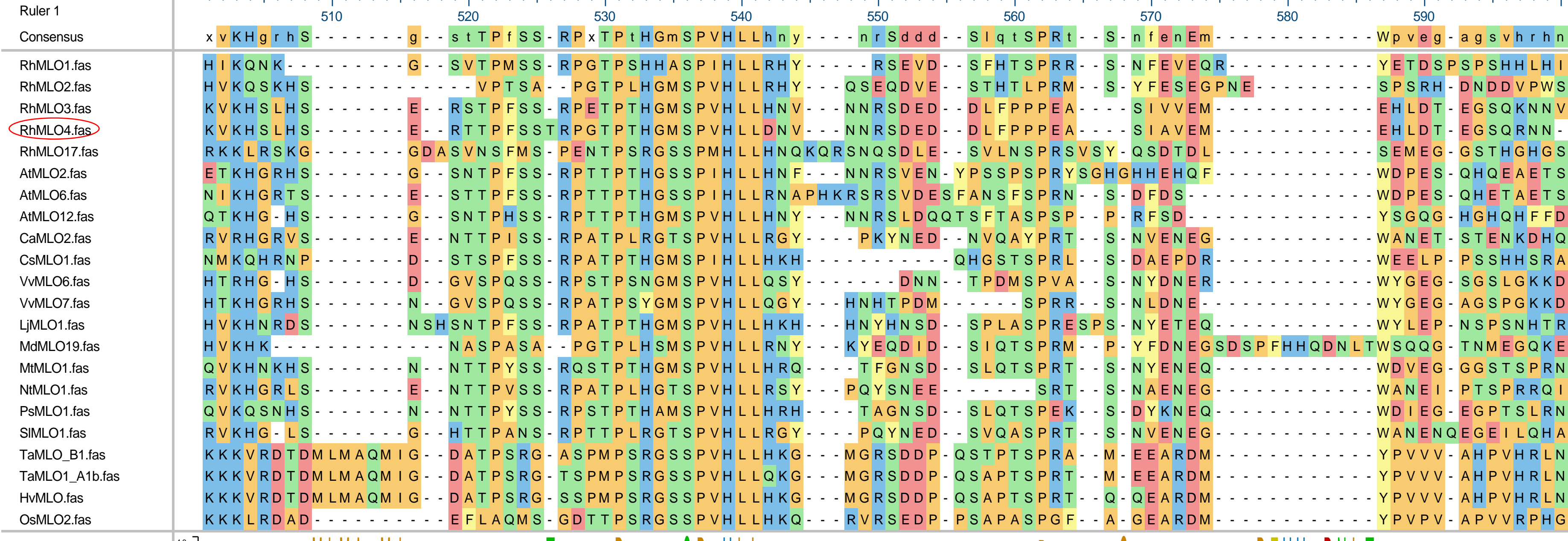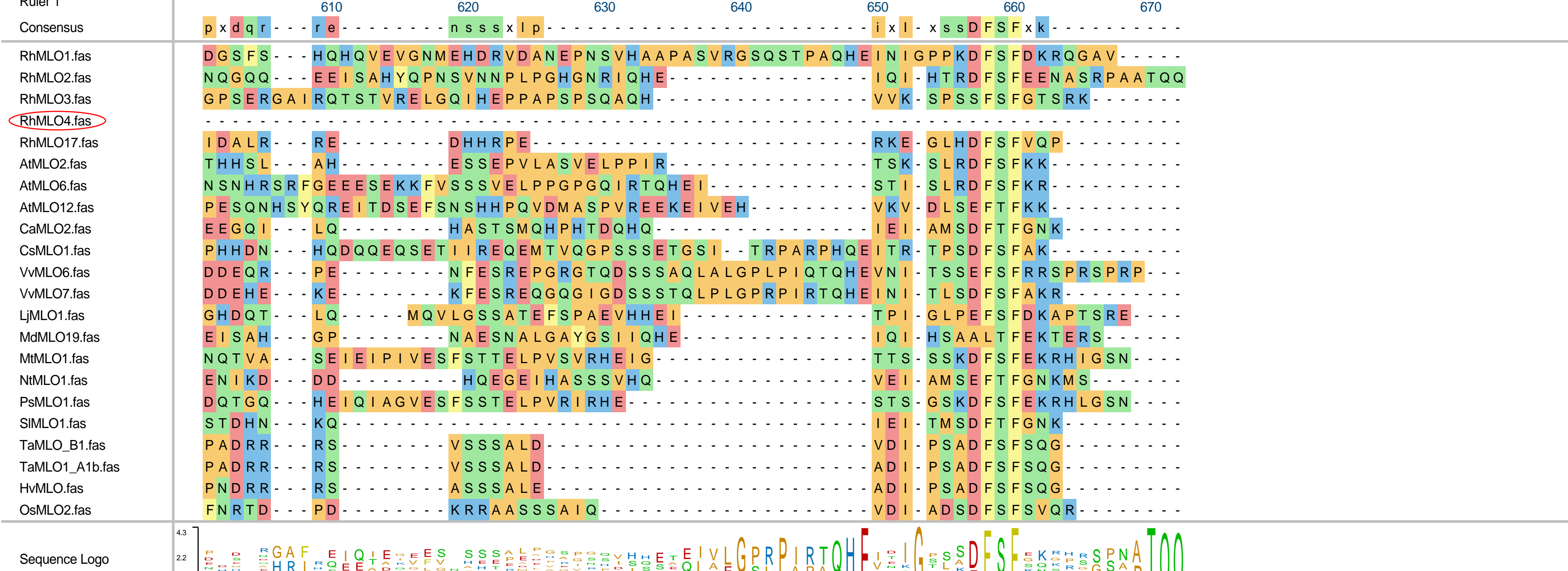

Supplement: Supplementary file 11 — Supplementary file11 (PDF 8427 KB) [file 122_2021_3838_MOESM11_ESM.pdf]

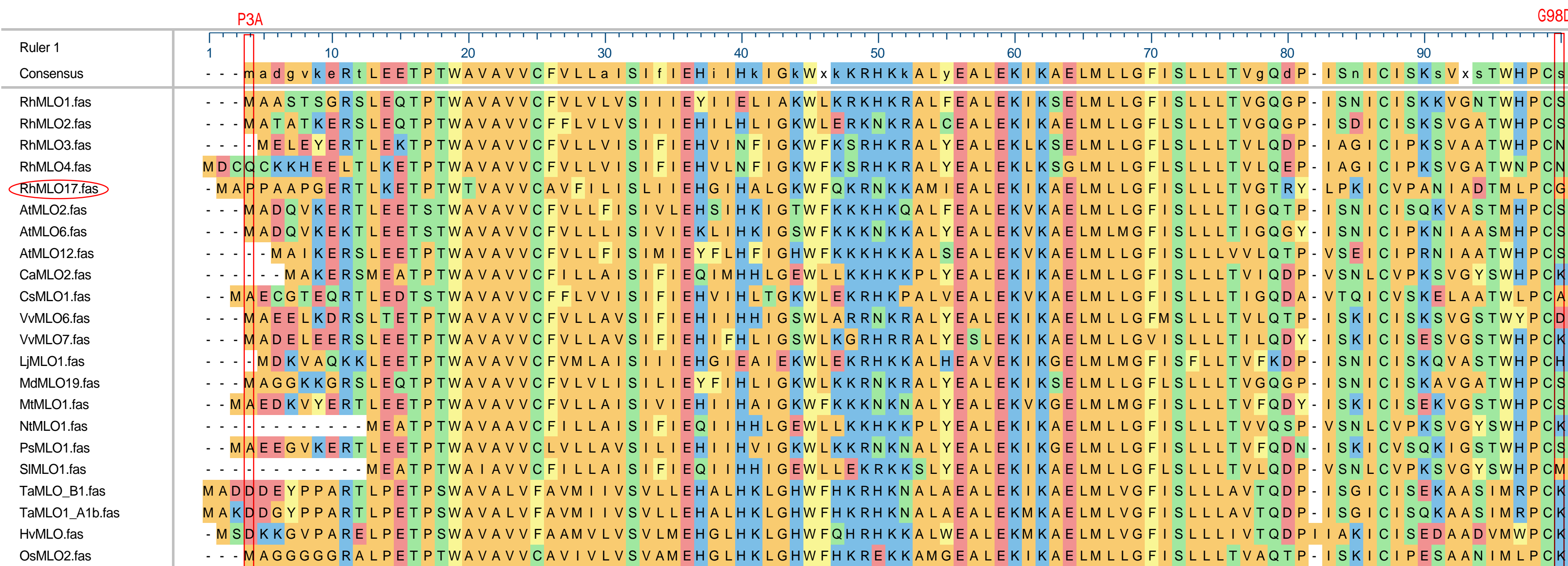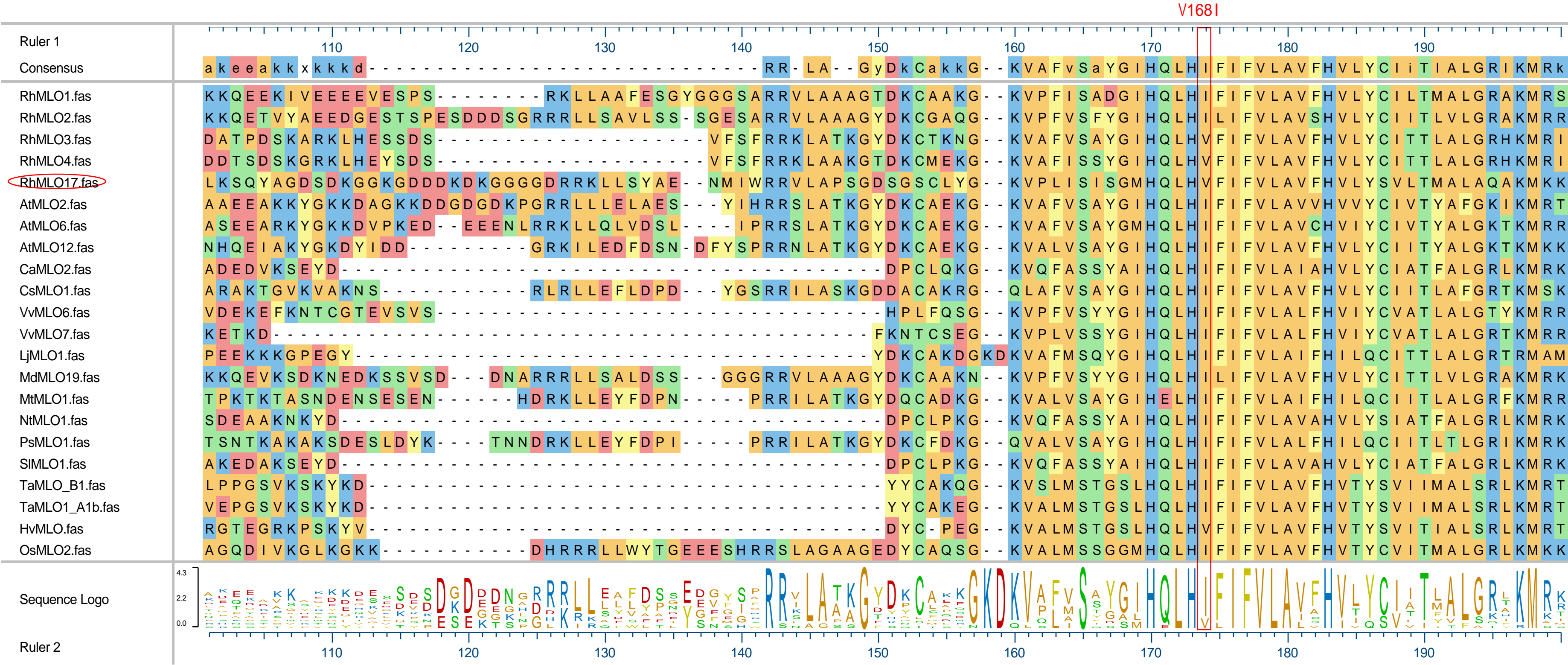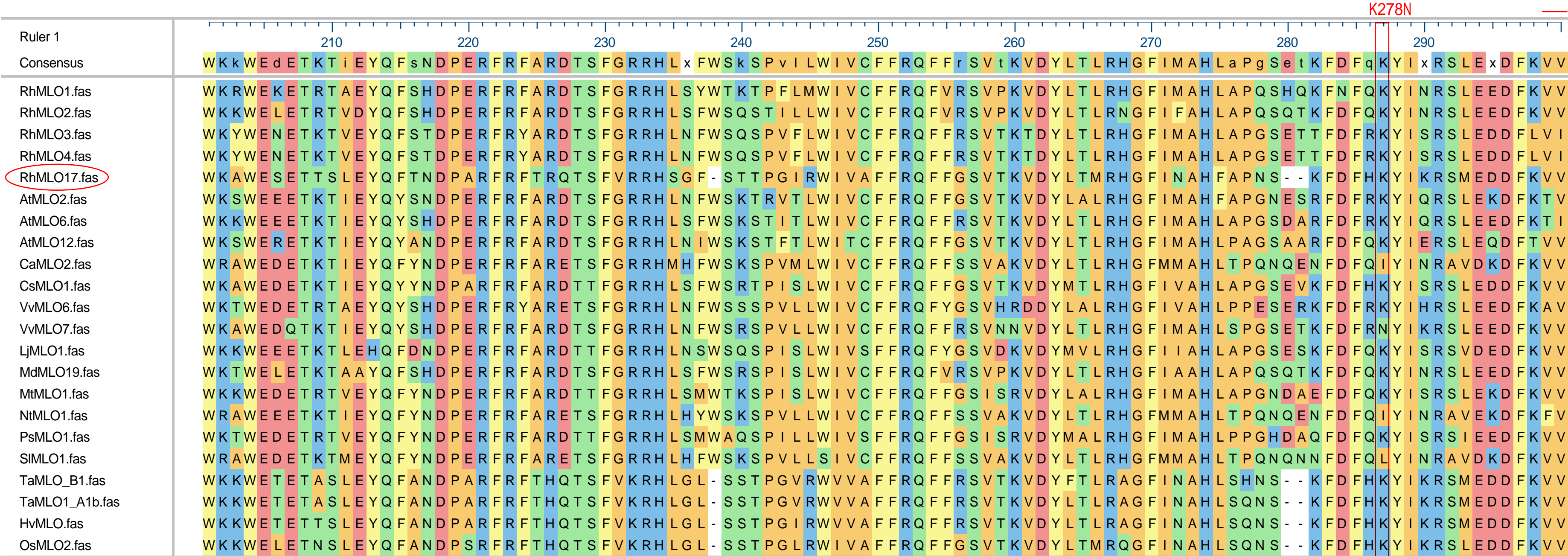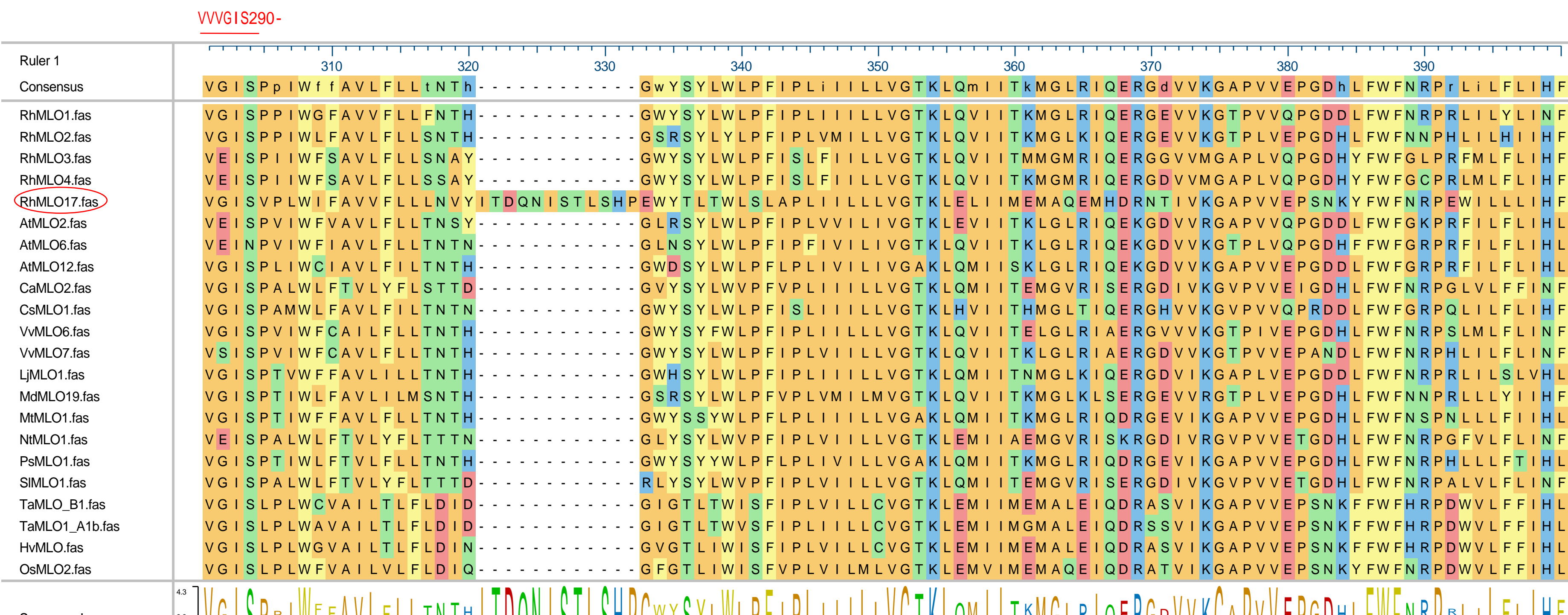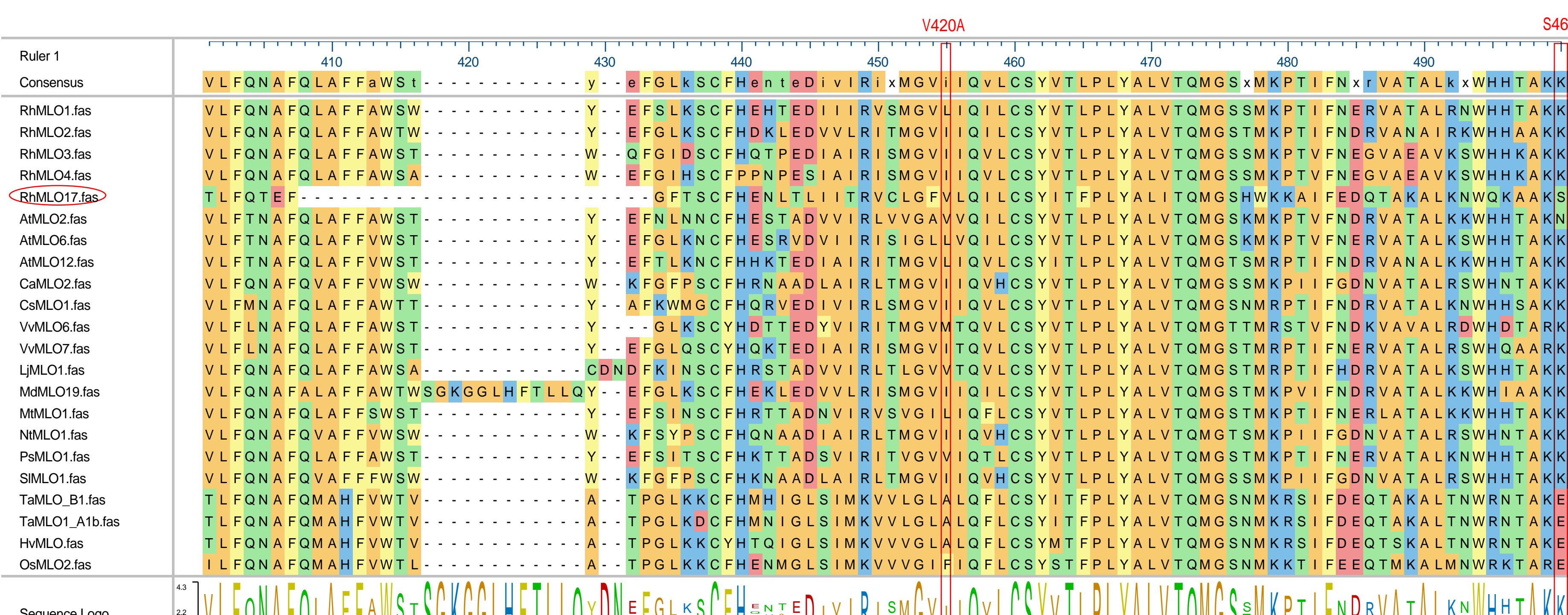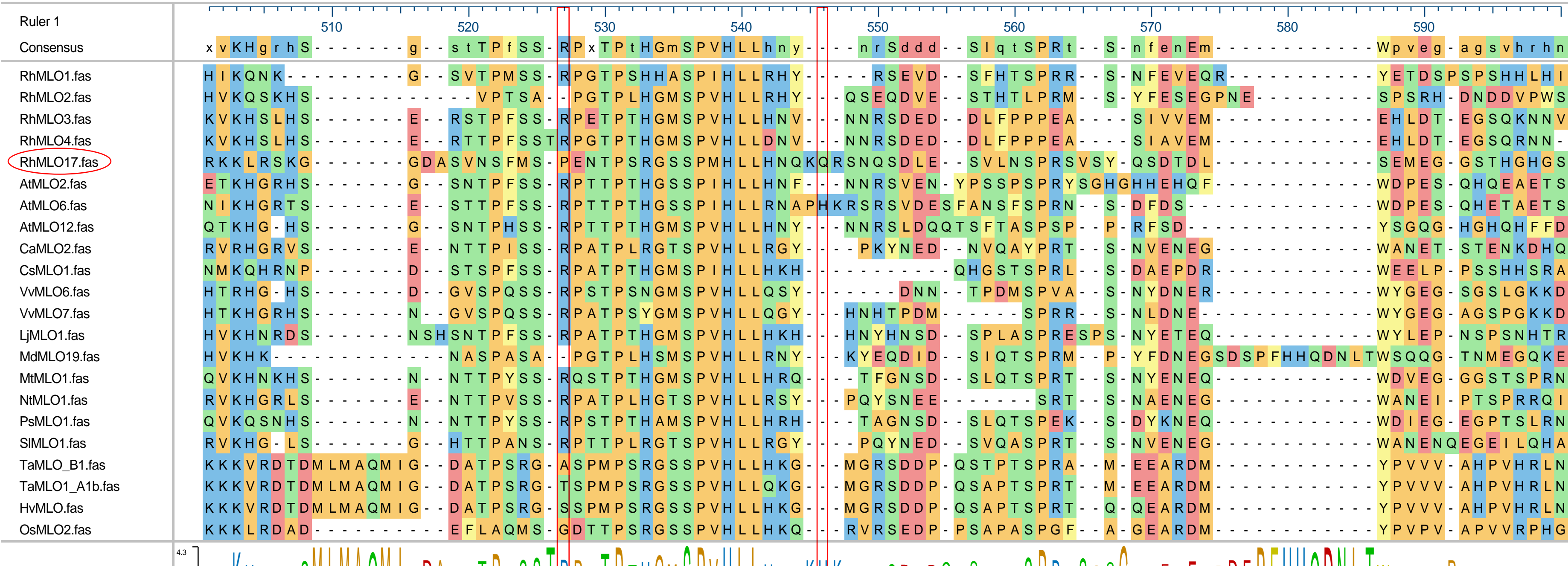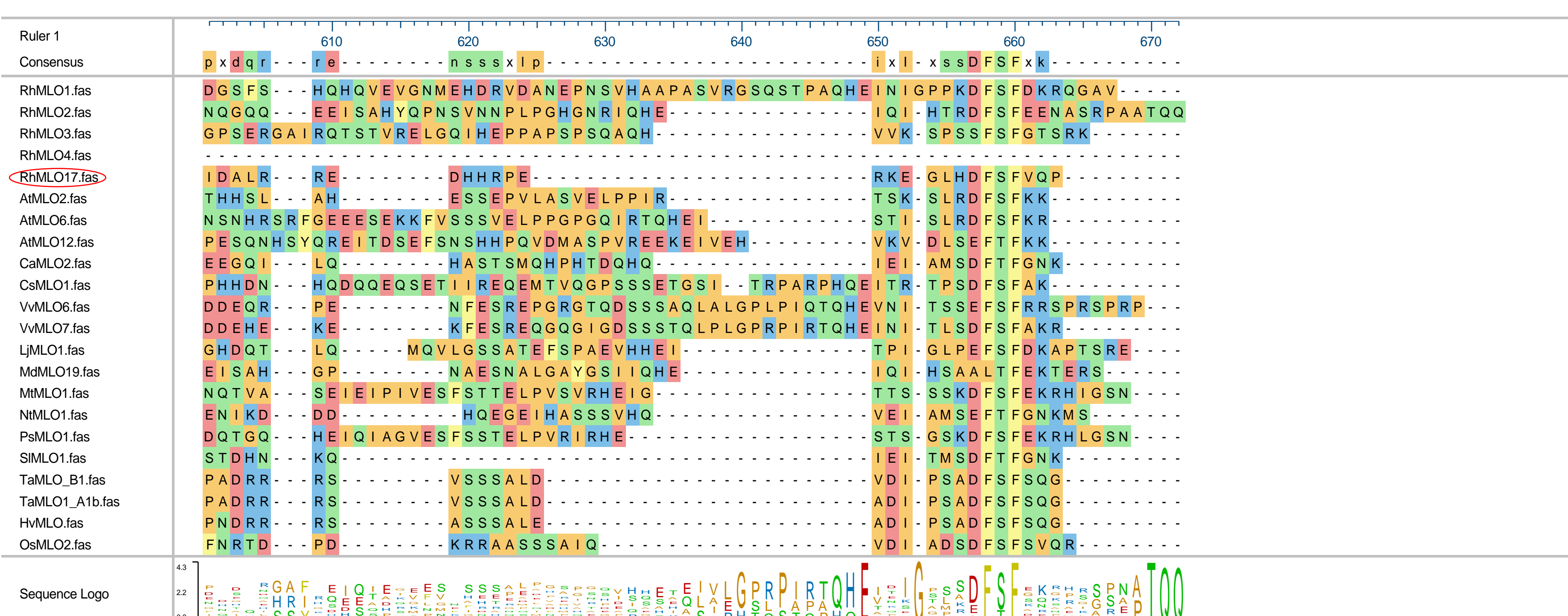

Supplement: Supplementary file 12 — Supplementary file12 (PDF 8412 KB) [file 122_2021_3838_MOESM12_ESM.pdf]
