# Supplementary material for: Analysis of allelic variants of RhMLO genes in rose and functional studies on susceptibility to powdery mildew related to clade V homologs
Source: Theor Appl Genet. 2021 May 2;134(8):2495–515. doi: 10.1007/s00122-021-03838-7 (PMC8277636; doi:10.1007/s00122-021-03838-7)
Supplement: Supplementary file 13 — Supplementary file13 (DOCX 17 KB) [file 122_2021_3838_MOESM13_ESM.docx]

**Supplementary Table S1.** Primer sequences used in study.

| Primer | Primer sequence |
| --- | --- |
| RhMLO1-F | 5’-GGGGACAAGTTTGTACAAAAAAGCAGGCTCC AGGCACTCCATCCCACCATG-3’ |
| RhMLO1-R | 5’-GGGGACCACTTTGTACAAGAAAGCTGGGTC TTATACAGCACCTTGTCTCTTATCAAATG-3’ |
| RhMLO2-F | 5’-GGGGACAAGTTTGTACAAAAAAGCAGGCTCC TATCAATCCGAACAGGACGTGG-3’ |
| RhMLO2-R | 5’-GGGGACCACTTTGTACAAGAAAGCTGGGTC TCATTGTTGTGTTGCAGCTGGTC-3’ |
| RhMLO3/4-F | 5’-GGGGACAAGTTTGTACAAAAAAGCAGGCTCC AGGGATGGCAGAAGCAGTG-3’ |
| RhMLO3/4-R | 5’-GGGGACCACTTTGTACAAGAAAGCTGGGTC TCACTTTCTTGATGTACCAAATGAG-3’ |
| RhActin5-F | 5’-GAGCGTTTCAGATGCCCAGA-3’ |
| RhActin5-R | 5’-TGGTGGGGCAACCACCTTA-3’ |
| RhUBI2-F | 5’-CACAAGCACGCAAACCCTAT-3’ |
| RhUBI2-R | 5’-GGAGCATGAGCCAAATGGAG-3’ |
| ITS-F | 5'-CGTAGGTGAACCTGCGGAAGGAT-3' |
| ITS-R | 5'-TGCACGAGTTGGGGCTTCTCTG-3' |
| RhMLO1-F | 5’-CCACAGCTCTACGCAACTGGCATCACA-3’ |
| RhMLO1-R | 5’-AAGCTGTCCACCTCGCTGCGGTAATGC-3’ |
| RhMLO2-F | 5’-AGTGGCACCATGCAGCAAAGAAGCA-3’ |
| RhMLO2-R | 5’-CGAGATGGGGACTCGTTTGGACCTTCA-3’ |
| RhMLO3-F | 5’-CCAAACCCCTGAAGATATCGCCATCAG-3’ |
| RhMLO3-R | 5’-GATGCCAACTTTTCACTGCTTCTGCCA-3’ |
