# Supplementary material for: Analysis of allelic variants of RhMLO genes in rose and functional studies on susceptibility to powdery mildew related to clade V homologs
Source: Theor Appl Genet. 2021 May 2;134(8):2495–515. doi: 10.1007/s00122-021-03838-7 (PMC8277636; doi:10.1007/s00122-021-03838-7)
Supplement: Supplementary file 14 — Supplementary file14 (DOCX 103 KB) [file 122_2021_3838_MOESM14_ESM.docx]

***R.chinensis var. spontanea*:18**

| Query | Subject | Identity % | Alignment length | mismatches | Gap opens | q.start | q.end | s.start | s.end | e-value | Bit score | Length of subject |
| --- | --- | --- | --- | --- | --- | --- | --- | --- | --- | --- | --- | --- |
| RhMLO1 | DE_g1140 | 100 | 591 | 0 | 0 | 1 | 591 | 1 | 591 | 0 | 1225 | 591 |
| RhMLO2 | DE_g28536 | 97.959 | 588 | 0 | 1 | 1 | 588 | 1 | 576 | 0 | 1186 | 576 |
| RhMLO3 | DE_g72155 | 97.674 | 172 | 4 | 0 | 1 | 172 | 1 | 172 | 2.88E-118 | 347 | 177 |
| RhMLO4 | DE_g51772 | 99.505 | 404 | 2 | 0 | 1 | 404 | 1 | 404 | 0 | 832 | 404 |
| RhMLO5 | DE_g56341 | 99.19 | 247 | 2 | 0 | 273 | 519 | 87 | 333 | 6.85E-179 | 506 | 333 |
| RhMLO6 | DE_g27540 | 89.512 | 410 | 0 | 1 | 1 | 410 | 1 | 367 | 0 | 731 | 377 |
| RhMLO7 | DE_g26611 | 100 | 541 | 0 | 0 | 1 | 541 | 1 | 541 | 0 | 1127 | 541 |
| RhMLO8 | DE_g45024 | 100 | 508 | 0 | 0 | 1 | 508 | 1 | 508 | 0 | 1044 | 508 |
| RhMLO9 | DE_g21721 | 86.17 | 564 | 45 | 4 | 1 | 562 | 1 | 533 | 0 | 940 | 533 |
| RhMLO10 | DE_g18014 | 98.981 | 589 | 4 | 1 | 1 | 587 | 1 | 589 | 0 | 1188 | 589 |
| RhMLO11 | DE_g18735 | 93.023 | 559 | 5 | 3 | 1 | 550 | 1 | 534 | 0 | 1043 | 534 |
| RhMLO12 | DE_g17395 | 93.333 | 495 | 9 | 2 | 43 | 516 | 926 | 1417 | 0 | 946 | 1417 |
| RhMLO13 | DE_g22501 | 84.268 | 642 | 16 | 4 | 1 | 641 | 1 | 558 | 0 | 1055 | 558 |
| RhMLO14 | DE_g27084 | 88.498 | 626 | 4 | 4 | 1 | 624 | 1 | 560 | 0 | 1106 | 560 |
| RhMLO15 | DE_g96 | 99.808 | 521 | 1 | 0 | 1 | 521 | 1 | 521 | 0 | 1080 | 521 |
| RhMLO16 | DE_g39975 | 90.761 | 552 | 1 | 1 | 1 | 552 | 1 | 502 | 0 | 1019 | 502 |
| RhMLO17 | DE_g61092 | 88.66 | 388 | 13 | 2 | 192 | 567 | 1 | 369 | 0 | 697 | 369 |
| RhMLO18 | DE_g2325 | 99.198 | 499 | 4 | 0 | 1 | 499 | 1 | 499 | 0 | 1029 | 500 |
| RhMLO19 |  |  |  |  |  |  |  |  |  |  |  |  |

***R.laevigata*:18**

| Query | Subject | Identity % | Alignment length | mismatches | Gap opens | q.start | q.end | s.start | s.end | e-value | Bit score | Length of subject | Note | Allele |
| --- | --- | --- | --- | --- | --- | --- | --- | --- | --- | --- | --- | --- | --- | --- |
| RhMLO1 | DH_g19368 | 97.631 | 591 | 14 | 0 | 1 | 591 | 1 | 591 | 0 | 1202 | 664 |  |  |
| RhMLO2 | DH_g48532 | 93.878 | 588 | 20 | 2 | 1 | 588 | 1 | 572 | 0 | 1075 | 572 |  |  |
| RhMLO3 | DH_g60275 | 88.8 | 375 | 16 | 1 | 1 | 349 | 347 | 721 | 0 | 653 | 1044 |  |  |
| RhMLO4 |  |  |  |  |  |  |  |  |  |  |  |  |  |  |
| RhMLO5 | DH_g21686 | 85.185 | 540 | 5 | 4 | 1 | 519 | 1 | 486 | 0 | 835 | 486 |  |  |
| RhMLO6 | DH_g19989 | 88.293 | 410 | 27 | 2 | 1 | 410 | 1 | 389 | 0 | 720 | 399 |  |  |
| RhMLO7 | DH_g30101 | 97.417 | 542 | 13 | 1 | 1 | 541 | 1 | 542 | 0 | 1097 | 542 |  |  |
| RhMLO8 | DH_g15341 | 96.267 | 509 | 17 | 2 | 1 | 508 | 1 | 508 | 0 | 1000 | 508 |  |  |
| RhMLO9 | DH_g9560 | 83.894 | 565 | 56 | 4 | 1 | 562 | 1 | 533 | 0 | 895 | 533 |  |  |
| RhMLO10 | DH_g27616 | 93.888 | 589 | 14 | 5 | 1 | 587 | 1 | 569 | 0 | 1107 | 569 |  |  |
| RhMLO11 | DH_g3456 | 90.311 | 547 | 19 | 3 | 1 | 538 | 1 | 522 | 0 | 937 | 543 |  |  |
| RhMLO12 | DH_g39713 | 83.829 | 538 | 14 | 4 | 1 | 516 | 1 | 487 | 0 | 887 | 487 |  |  |
| RhMLO13 | DH_g42387 | 84.095 | 547 | 72 | 2 | 61 | 607 | 1 | 532 | 0 | 915 | 718 | c | 2 |
| RhMLO13 | DH_g62326 | 92.57 | 323 | 10 | 1 | 319 | 641 | 1 | 309 | 0 | 602 | 309 | b | 1b |
| RhMLO13 | DH_g9881 | 94.22 | 173 | 10 | 0 | 1 | 173 | 1 | 173 | 1.09E-111 | 333 | 180 | a | 1a |
| RhMLO14 |  |  |  |  |  |  |  |  |  |  |  |  |  |  |
| RhMLO15 | DH_g1179 | 97.876 | 518 | 8 | 1 | 1 | 518 | 1 | 515 | 0 | 1041 | 523 |  |  |
| RhMLO16 | DH_g2842 | 83.274 | 562 | 21 | 3 | 1 | 539 | 1 | 512 | 0 | 946 | 1136 |  |  |
| RhMLO17 | DH_g49208 | 88.083 | 579 | 46 | 3 | 1 | 567 | 1 | 568 | 0 | 1036 | 568 |  |  |
| RhMLO18 | DH_g16398 | 91.383 | 499 | 42 | 1 | 1 | 499 | 1 | 498 | 0 | 902 | 499 |  |  |
| RhMLO19 |  |  |  |  |  |  |  |  |  |  |  |  |  |  |

***R.moschata*:22**

| Query | Subject | Identity % | Alignment length | mismatches | Gap opens | q.start | q.end | s.start | s.end | e-value | Bit score | Length of subject | Note | Allele |
| --- | --- | --- | --- | --- | --- | --- | --- | --- | --- | --- | --- | --- | --- | --- |
| RhMLO1 | DI_g24871 | 97.8 | 591 | 12 | 1 | 1 | 591 | 1 | 590 | 0 | 1195 | 590 |  |  |
| RhMLO2 | DI_g26170 | 94.898 | 588 | 14 | 2 | 1 | 588 | 1 | 572 | 0 | 1139 | 572 |  |  |
| RhMLO3 |  |  |  |  |  |  |  |  |  |  |  |  |  |  |
| RhMLO4 | DI_g43941 | 93.467 | 199 | 13 | 0 | 114 | 312 | 18 | 216 | 1.60E-134 | 389 | 239 |  |  |
| RhMLO5 | DI_g38248 | 89.407 | 236 | 3 | 2 | 60 | 274 | 1 | 235 | 4.95E-147 | 425 | 335 | a | 1a |
| RhMLO5 | DI_g38247 | 77.692 | 260 | 13 | 2 | 260 | 519 | 102 | 316 | 3.15E-117 | 348 | 316 | b | 1b |
| RhMLO6 | DI_g35044 | 99.606 | 254 | 1 | 0 | 1 | 254 | 1 | 254 | 0 | 524 | 288 | a | 1a |
| RhMLO6 | DI_g56702 | 93.805 | 113 | 7 | 0 | 298 | 410 | 1 | 113 | 5.86E-72 | 225 | 211 | b | 1b |
| RhMLO7 | DI_g50709 | 97.053 | 543 | 14 | 2 | 1 | 541 | 1 | 543 | 0 | 1088 | 543 |  |  |
| RhMLO8 | DI_g36405 | 97.047 | 508 | 10 | 2 | 1 | 508 | 1 | 503 | 0 | 1000 | 503 |  |  |
| RhMLO9 | DI_g26059 | 97.756 | 312 | 7 | 0 | 251 | 562 | 3 | 314 | 0 | 634 | 314 | b | 1b |
| RhMLO9 | DI_g26060 | 98.507 | 134 | 2 | 0 | 64 | 197 | 1 | 134 | 8.21E-91 | 275 | 142 | a | 1a |
| RhMLO10 | DI_g28589 | 93.623 | 345 | 9 | 2 | 243 | 587 | 14 | 345 | 0 | 606 | 345 | b | 1b |
| RhMLO10 | DI_g35300 | 96.789 | 218 | 4 | 1 | 1 | 215 | 1 | 218 | 5.46E-146 | 421 | 220 | a | 1a |
| RhMLO11 | DI_g21592 | 91.344 | 543 | 13 | 3 | 1 | 534 | 1 | 518 | 0 | 936 | 543 |  |  |
| RhMLO12 | DI_g57576 | 90.747 | 281 | 5 | 1 | 257 | 516 | 70 | 350 | 0 | 514 | 350 |  |  |
| RhMLO13 | DI_g23054 | 86.895 | 641 | 23 | 4 | 1 | 641 | 1 | 580 | 0 | 1061 | 580 | a | 1 |
| RhMLO13 | DI_g56777 | 82.995 | 641 | 79 | 3 | 1 | 641 | 1 | 611 | 0 | 1025 | 611 | b | 2 |
| RhMLO14 | DI_g10020 | 77.29 | 524 | 24 | 7 | 103 | 624 | 1 | 431 | 0 | 783 | 431 |  |  |
| RhMLO15 | DI_g18443 | 99.04 | 521 | 5 | 0 | 1 | 521 | 1 | 521 | 0 | 1071 | 521 |  |  |
| RhMLO16 | DI_g7415 | 89.855 | 552 | 6 | 1 | 1 | 552 | 1 | 502 | 0 | 1011 | 502 |  |  |
| RhMLO17 | DI_g24909 | 87.737 | 579 | 48 | 3 | 1 | 567 | 1 | 568 | 0 | 1031 | 568 |  |  |
| RhMLO18 | DI_g6134 | 92.786 | 499 | 36 | 0 | 1 | 499 | 1 | 499 | 0 | 918 | 500 |  |  |
| RhMLO19 |  |  |  |  |  |  |  |  |  |  |  |  |  |  |

***R.minutifolia*:19**

| Query | Subject | Identity % | Alignment length | mismatches | Gap opens | q.start | q.end | s.start | s.end | e-value | Bit score | Length of subject | Note | Allele |
| --- | --- | --- | --- | --- | --- | --- | --- | --- | --- | --- | --- | --- | --- | --- |
| RhMLO1 | DG_g21834 | 98.139 | 591 | 11 | 0 | 1 | 591 | 1 | 591 | 0 | 1205 | 591 |  |  |
| RhMLO2 | DG_g25343 | 95.073 | 548 | 11 | 2 | 41 | 588 | 22 | 553 | 0 | 1067 | 553 |  |  |
| RhMLO3 | DG_g4598 | 93.606 | 563 | 34 | 1 | 1 | 563 | 1 | 561 | 0 | 1040 | 561 |  |  |
| RhMLO4 |  |  |  |  |  |  |  |  |  |  |  |  |  |  |
| RhMLO5 | DG_g51235 | 81.967 | 244 | 1 | 1 | 276 | 519 | 309 | 509 | 1.07E-131 | 392 | 509 |  |  |
| RhMLO6 | DG_g13051 | 100 | 219 | 0 | 0 | 1 | 219 | 1 | 219 | 7.50E-160 | 453 | 337 |  |  |
| RhMLO7 | DG_g36070 | 96.269 | 402 | 10 | 1 | 145 | 541 | 54 | 455 | 0 | 805 | 455 |  |  |
| RhMLO8 | DG_g46739 | 97.835 | 508 | 8 | 1 | 1 | 508 | 1 | 505 | 0 | 1016 | 505 |  |  |
| RhMLO9 | DG_g23343 | 97.436 | 312 | 8 | 0 | 251 | 562 | 22 | 333 | 0 | 633 | 333 | b | 1b |
| RhMLO9 | DG_g23342 | 99.492 | 197 | 1 | 0 | 1 | 197 | 1 | 197 | 9.53E-140 | 403 | 205 | a | 1a |
| RhMLO10 | DG_g9046 | 95.455 | 594 | 7 | 4 | 1 | 587 | 1 | 581 | 0 | 1136 | 581 |  |  |
| RhMLO11 | DG_g5922 | 91.344 | 543 | 13 | 3 | 1 | 534 | 1 | 518 | 0 | 938 | 543 |  |  |
| RhMLO12 | DG_g13384 | 93.145 | 496 | 6 | 3 | 42 | 516 | 935 | 1423 | 0 | 945 | 1423 |  |  |
| RhMLO13 | DG_g15066 | 86.271 | 641 | 27 | 4 | 1 | 641 | 1 | 580 | 0 | 1036 | 580 | a | 1 |
| RhMLO13 | DG_g39748 | 83.444 | 453 | 61 | 1 | 189 | 641 | 4 | 442 | 0 | 735 | 442 | b | 2 |
| RhMLO14 | DG_g18392 | 79.073 | 626 | 25 | 8 | 1 | 624 | 1 | 522 | 0 | 937 | 522 |  |  |
| RhMLO15 | DG_g9782 | 99.424 | 521 | 3 | 0 | 1 | 521 | 1 | 521 | 0 | 1074 | 521 |  |  |
| RhMLO16 | DG_g11167 | 89.855 | 552 | 6 | 1 | 1 | 552 | 1 | 502 | 0 | 1010 | 502 |  |  |
| RhMLO17 | DG_g14998 | 90.674 | 579 | 23 | 2 | 1 | 567 | 1 | 560 | 0 | 1024 | 560 |  |  |
| RhMLO18 | DG_g2703 | 92.385 | 499 | 36 | 1 | 1 | 499 | 1 | 497 | 0 | 907 | 498 |  |  |
| RhMLO19 |  |  |  |  |  |  |  |  |  |  |  |  |  |  |

***R. persica*:18**

| Query | Subject | Identity % | Alignment length | mismatches | Gap opens | q.start | q.end | s.start | s.end | e-value | Bit score | Length of subject | Note | Allele |
| --- | --- | --- | --- | --- | --- | --- | --- | --- | --- | --- | --- | --- | --- | --- |
| RhMLO1 | DL_g47157.t1 | 96.324 | 272 | 10 | 0 | 320 | 591 | 1 | 272 | 0 | 550 | 272 |  |  |
| RhMLO2 | DL_g13332.t1 | 82.529 | 601 | 73 | 7 | 1 | 588 | 1 | 582 | 0 | 925 | 582 |  |  |
| RhMLO3 | DL_g3054.t1 | 92.895 | 563 | 21 | 1 | 1 | 563 | 1 | 544 | 0 | 1018 | 544 |  |  |
| RhMLO4 |  |  |  |  |  |  |  |  |  |  |  |  |  |  |
| RhMLO5 | DL_g35297.t1 | 79.823 | 565 | 14 | 5 | 1 | 519 | 1 | 511 | 0 | 824 | 511 |  |  |
| RhMLO6 | DL_g26969.t1 | 93.659 | 205 | 12 | 1 | 1 | 205 | 1 | 204 | 4.71E-136 | 392 | 316 |  |  |
| RhMLO7 | DL_g8965.t1 | 91.683 | 517 | 42 | 1 | 1 | 516 | 1 | 517 | 0 | 943 | 580 |  |  |
| RhMLO8 | DL_g22569.t1 | 95.481 | 509 | 16 | 3 | 1 | 508 | 1 | 503 | 0 | 983 | 503 |  |  |
| RhMLO9 | DL_g39075.t1 | 94.138 | 290 | 17 | 0 | 273 | 562 | 199 | 488 | 0 | 545 | 488 |  |  |
| RhMLO10 | DL_g25357.t1 | 86.882 | 587 | 13 | 6 | 1 | 587 | 1 | 523 | 0 | 1000 | 523 |  |  |
| RhMLO11 | DL_g45919.t1 | 90.055 | 543 | 20 | 3 | 1 | 534 | 1 | 518 | 0 | 919 | 543 |  |  |
| RhMLO12 | DL_g21102.t1 | 96.8 | 125 | 4 | 0 | 392 | 516 | 3 | 127 | 1.12E-86 | 263 | 127 |  |  |
| RhMLO13 | DL_g22247.t1 | 88.07 | 285 | 20 | 1 | 357 | 641 | 23 | 293 | 3.42E-166 | 476 | 293 |  |  |
| RhMLO14 | DL_g38112.t1 | 85.163 | 337 | 23 | 3 | 289 | 623 | 169 | 480 | 0 | 531 | 481 |  |  |
| RhMLO15 | DL_g19709.t1 | 97.374 | 457 | 12 | 0 | 42 | 498 | 760 | 1216 | 0 | 933 | 1216 |  |  |
| RhMLO16 | DL_g29405.t1 | 81.574 | 559 | 59 | 4 | 1 | 552 | 1 | 522 | 0 | 911 | 522 |  |  |
| RhMLO17 | DL_g55109.t1 | 91.096 | 292 | 21 | 1 | 1 | 292 | 1 | 287 | 0 | 534 | 323 | a | 1a |
| RhMLO17 | DL_g32145.t1 | 93.077 | 130 | 9 | 0 | 438 | 567 | 4 | 133 | 5.62E-81 | 249 | 133 | b | 1b |
| RhMLO18 | DL_g35723.t1 | 90.419 | 334 | 32 | 0 | 166 | 499 | 1 | 334 | 0 | 589 | 335 |  |  |
| RhMLO19 |  |  |  |  |  |  |  |  |  |  |  |  |  |  |

***R.rugosa*:32**

| Query | Subject | Identity % | Alignment length | mismatches | Gap opens | q.start | q.end | s.start | s.end | e-value | Bit score | Length of subject | Note | Allele |
| --- | --- | --- | --- | --- | --- | --- | --- | --- | --- | --- | --- | --- | --- | --- |
| RhMLO1 | g32164.t1 | 94.581 | 203 | 11 | 0 | 1 | 203 | 1 | 203 | 1.62E-125 | 373 | 328 | b | 2 |
| RhMLO1 | g101964.t1 | 96.757 | 185 | 5 | 1 | 1 | 184 | 1 | 185 | 1.48E-121 | 358 | 199 | a | 1 |
| RhMLO2 | g73771.t1 | 98.333 | 300 | 5 | 0 | 1 | 300 | 1 | 300 | 0 | 606 | 328 | a | 1a |
| RhMLO2 | g62934.t1 | 89.868 | 227 | 11 | 1 | 343 | 569 | 1 | 215 | 3.74E-146 | 421 | 218 | b | 1b |
| RhMLO3 | g3749.t1 | 95.737 | 563 | 24 | 0 | 1 | 563 | 399 | 961 | 0 | 1028 | 961 | a | 1 |
| RhMLO3 | g72264.t1 | 78.02 | 505 | 43 | 5 | 72 | 563 | 11 | 460 | 0 | 741 | 460 | b | 2 |
| RhMLO4 | g38895.t1 | 94.027 | 519 | 30 | 1 | 1 | 519 | 1 | 518 | 0 | 955 | 564 |  |  |
| RhMLO5 | g63967.t1 | 90.678 | 236 | 1 | 1 | 60 | 274 | 1 | 236 | 2.49E-148 | 434 | 471 | a | 1a |
| RhMLO5 | g94782.t1 | 89.407 | 236 | 3 | 2 | 60 | 274 | 1 | 235 | 4.94E-147 | 425 | 335 | b | 2a |
| RhMLO5 | g70217.t1 | 96 | 125 | 5 | 0 | 395 | 519 | 11 | 135 | 7.97E-71 | 223 | 135 | c | b |
| RhMLO6 | g8880.t1 | 99.213 | 254 | 2 | 0 | 1 | 254 | 1 | 254 | 0 | 523 | 288 | a | 1a |
| RhMLO6 | g92923.t1 | 94.69 | 113 | 6 | 0 | 298 | 410 | 1 | 113 | 9.11E-73 | 226 | 166 | b | 1b |
| RhMLO7 | g59321.t1 | 97.315 | 298 | 6 | 2 | 1 | 296 | 1 | 298 | 0 | 595 | 308 |  |  |
| RhMLO8 | g68400.t1 | 98.195 | 277 | 5 | 0 | 1 | 277 | 1 | 277 | 0 | 563 | 277 |  |  |
| RhMLO9 | g46432.t1 | 98.985 | 197 | 2 | 0 | 1 | 197 | 1 | 197 | 1.14E-138 | 400 | 205 | a | 1a |
| RhMLO9 | g101133.t1 | 97.838 | 185 | 4 | 0 | 378 | 562 | 12 | 196 | 7.46E-128 | 373 | 196 | b | 1b |
| RhMLO10 | g67434.t1 | 97.484 | 159 | 3 | 1 | 429 | 587 | 1 | 158 | 7.04E-106 | 316 | 158 | b1 | 1b |
| RhMLO10 | g31930.t1 | 97.484 | 159 | 3 | 1 | 429 | 587 | 1 | 158 | 2.17E-105 | 315 | 158 | b2 | 2b |
| RhMLO10 | g3442.t1 | 94.479 | 163 | 6 | 1 | 1 | 160 | 1 | 163 | 1.05E-96 | 293 | 172 | a | a |
| RhMLO11 | g92174.t1 | 96.479 | 284 | 9 | 1 | 266 | 548 | 1 | 284 | 0 | 560 | 295 | b | 1b |
| RhMLO11 | g64058.t1 | 94.915 | 236 | 4 | 1 | 1 | 228 | 1 | 236 | 1.55E-147 | 425 | 270 | a | 1a |
| RhMLO12 | g41269.t1 | 91.622 | 370 | 7 | 2 | 43 | 391 | 567 | 933 | 0 | 695 | 943 |  |  |
| RhMLO13 | g51762.t1 | 93.498 | 323 | 6 | 2 | 319 | 641 | 1 | 308 | 0 | 603 | 308 | a1 | 1 |
| RhMLO13 | g10550.t1 | 93.498 | 323 | 6 | 2 | 319 | 641 | 1 | 308 | 0 | 603 | 308 | a2 | 2 |
| RhMLO13 | g11267.t1 | 86.266 | 233 | 32 | 0 | 409 | 641 | 1 | 233 | 1.89E-130 | 384 | 233 | b | 3 |
| RhMLO14 | g71114.t1 | 95.918 | 98 | 4 | 0 | 103 | 200 | 1 | 98 | 1.56E-51 | 173 | 106 |  |  |
| RhMLO15 | g53393.t1 | 85.556 | 360 | 2 | 1 | 1 | 360 | 1 | 310 | 0 | 613 | 314 | a | 1a |
| RhMLO15 | g4611.t1 | 98.137 | 161 | 3 | 0 | 361 | 521 | 10 | 170 | 1.95E-111 | 328 | 170 | b | 1b |
| RhMLO16 | g102350.t1 | 100 | 116 | 0 | 0 | 255 | 370 | 13 | 128 | 5.94E-79 | 245 | 144 |  |  |
| RhMLO17 | g102354.t1 | 97.222 | 108 | 3 | 0 | 185 | 292 | 1 | 108 | 4.21E-71 | 224 | 122 |  |  |
| RhMLO18 | g67734.t1 | 92.444 | 225 | 17 | 0 | 1 | 225 | 1 | 225 | 1.45E-152 | 435 | 250 | a | 1a |
| RhMLO18 | g98288.t1 | 93.305 | 239 | 16 | 0 | 261 | 499 | 1 | 239 | 1.84E-145 | 417 | 240 | b | 1b |
| RhMLO19 |  |  |  |  |  |  |  |  |  |  |  |  |  |  |

***R.xanthina spontanea*:17**

| Query | Subject | Identity % | Alignment length | mismatches | Gap opens | q.start | q.end | s.start | s.end | e-value | Bit score | Length of subject |
| --- | --- | --- | --- | --- | --- | --- | --- | --- | --- | --- | --- | --- |
| RhMLO1 | DC_g24711 | 97.631 | 591 | 14 | 0 | 1 | 591 | 1 | 591 | 0 | 1201 | 815 |
| RhMLO2 | DC_g38822 | 95.748 | 588 | 13 | 1 | 1 | 588 | 1 | 576 | 0 | 1160 | 576 |
| RhMLO3 | DC_g1808 | 95.737 | 563 | 24 | 0 | 1 | 563 | 293 | 855 | 0 | 1052 | 855 |
| RhMLO4 |  |  |  |  |  |  |  |  |  |  |  |  |
| RhMLO5 | DC_g25694 | 84.815 | 540 | 7 | 4 | 1 | 519 | 1 | 486 | 0 | 855 | 486 |
| RhMLO6 | DC_g17119 | 95.61 | 205 | 8 | 1 | 1 | 205 | 1 | 204 | 2.14E-139 | 400 | 316 |
| RhMLO7 | DC_g31786 | 96.458 | 480 | 16 | 1 | 63 | 541 | 1 | 480 | 0 | 966 | 480 |
| RhMLO8 | DC_g14391 | 94.106 | 509 | 26 | 2 | 1 | 508 | 1 | 506 | 0 | 940 | 506 |
| RhMLO9 | DC_g12594 | 92.705 | 562 | 16 | 1 | 1 | 562 | 1 | 537 | 0 | 1065 | 537 |
| RhMLO10 | DC_g5880 | 93.526 | 587 | 25 | 2 | 1 | 587 | 1 | 574 | 0 | 1047 | 574 |
| RhMLO11 | DC_g22611 | 90.95 | 442 | 15 | 1 | 1 | 442 | 1 | 417 | 0 | 817 | 436 |
| RhMLO12 | DC_g2192 | 91.165 | 498 | 17 | 3 | 43 | 516 | 937 | 1431 | 0 | 884 | 1431 |
| RhMLO13 | DC_g7576 | 93.76 | 641 | 26 | 1 | 1 | 641 | 1 | 627 | 0 | 1182 | 627 |
| RhMLO14 | DC_g22411 | 84.202 | 614 | 27 | 5 | 1 | 612 | 1 | 546 | 0 | 1034 | 775 |
| RhMLO15 | DC_g5043 | 98.656 | 521 | 7 | 0 | 1 | 521 | 1 | 521 | 0 | 1067 | 521 |
| RhMLO16 | DC_g3093 | 86.415 | 530 | 53 | 2 | 1 | 521 | 1 | 520 | 0 | 930 | 663 |
| RhMLO17 | DC_g2200 | 83.932 | 585 | 58 | 5 | 1 | 567 | 1 | 567 | 0 | 966 | 567 |
| RhMLO18 | DC_g12831 | 92.184 | 499 | 39 | 0 | 1 | 499 | 1 | 499 | 0 | 914 | 500 |
| RhMLO19 |  |  |  |  |  |  |  |  |  |  |  |  |

**AH.RSEM**

| Query | Subject | Identity % | Alignment length | mismatches | Gap opens | q.start | q.end | s.start | s.end | e-value | Bit score |
| --- | --- | --- | --- | --- | --- | --- | --- | --- | --- | --- | --- |
| RhMLO1 | 2AH_comp34745_c0_seq1 | 100 | 591 | 0 | 0 | 1 | 591 | 141 | 1913 | 0 | 1083 |
| RhMLO2 | 2AH_comp90968_c0_seq1 | 91.736 | 121 | 6 | 1 | 468 | 588 | 398 | 48 | 8.48E-73 | 228 |
| RhMLO3 | 2AH_comp33601_c0_seq7 | 97.059 | 204 | 6 | 0 | 157 | 360 | 709 | 98 | 2.21E-144 | 415 |
| RhMLO5 | 2AH_comp22048_c0_seq1 | 99.234 | 261 | 2 | 0 | 259 | 519 | 3 | 785 | 4.81E-167 | 476 |
| RhMLO6 | 2AH_comp27117_c0_seq1 | 92.974 | 427 | 13 | 2 | 1 | 410 | 1559 | 279 | 0 | 799 |
| RhMLO8 | 2AH_comp34323_c0_seq1 | 93.91 | 509 | 27 | 2 | 1 | 508 | 59 | 1576 | 0 | 862 |
| RhMLO9 | 2AH_comp29489_c0_seq1 | 99.288 | 562 | 4 | 0 | 1 | 562 | 65 | 1750 | 0 | 1043 |
| RhMLO10 | 2AH_comp32300_c0_seq1 | 98.561 | 556 | 8 | 0 | 32 | 587 | 255 | 1922 | 0 | 1018 |
| RhMLO11 | 2AH_comp20459_c0_seq1 | 96.567 | 233 | 7 | 1 | 303 | 534 | 875 | 177 | 3.20E-121 | 358 |
| RhMLO12 | 2AH_comp30036_c0_seq2 | 98.054 | 514 | 7 | 1 | 3 | 516 | 1949 | 417 | 0 | 905 |
| RhMLO16 | 2AH_comp33673_c0_seq2 | 96.791 | 187 | 6 | 0 | 366 | 552 | 1181 | 621 | 9.86E-115 | 347 |
| RhMLO18 | 2AH_comp66466_c0_seq1 | 96.875 | 160 | 5 | 0 | 336 | 495 | 1 | 480 | 2.08E-100 | 298 |

DB.RSEM

| Query | Subject | Identity % | Alignment length | mismatches | Gap opens | q.start | q.end | s.start | s.end | e-value | Bit score |
| --- | --- | --- | --- | --- | --- | --- | --- | --- | --- | --- | --- |
| RhMLO1 | 6DB_comp21465_c0_seq1 | 99.56 | 455 | 2 | 0 | 137 | 591 | 2 | 1366 | 0 | 890 |
| RhMLO3 | 6DB_comp29149_c0_seq1 | 97.789 | 407 | 9 | 0 | 157 | 563 | 1441 | 221 | 0 | 752 |
| RhMLO6 | 6DB_comp22722_c0_seq1 | 93.677 | 427 | 10 | 2 | 1 | 410 | 1524 | 244 | 0 | 802 |
| RhMLO8 | 6DB_comp29608_c0_seq1 | 99.606 | 508 | 2 | 0 | 1 | 508 | 1794 | 271 | 0 | 893 |
| RhMLO9 | 6DB_comp32229_c0_seq2 | 99.288 | 562 | 4 | 0 | 1 | 562 | 219 | 1904 | 0 | 1043 |
| RhMLO10 | 6DB_comp28865_c0_seq1 | 99.101 | 556 | 5 | 0 | 32 | 587 | 1890 | 223 | 0 | 1023 |
| RhMLO11 | 6DB_comp65865_c0_seq1 | 98.343 | 181 | 2 | 1 | 343 | 522 | 2 | 544 | 2.96E-95 | 287 |
| RhMLO12 | 6DB_comp10179_c0_seq1 | 100 | 144 | 0 | 0 | 305 | 448 | 29 | 460 | 3.08E-100 | 298 |
| RhMLO14 | 6DB_comp12252_c0_seq1 | 100 | 100 | 0 | 0 | 293 | 392 | 1 | 300 | 2.85E-65 | 208 |
| RhMLO16 | 6DB_comp28918_c0_seq14 | 99.275 | 552 | 4 | 0 | 1 | 552 | 1039 | 2694 | 0 | 1104 |
| RhMLO18 | 6DB_comp154266_c0_seq1 | 93.75 | 80 | 5 | 0 | 417 | 496 | 3 | 242 | 5.83E-38 | 134 |

**GT.RSEM**

| Query | Subject | Identity % | Alignment length | mismatches | Gap opens | q.start | q.end | s.start | s.end | e-value | Bit score |
| --- | --- | --- | --- | --- | --- | --- | --- | --- | --- | --- | --- |
| RhMLO1 | 15GT_comp31617_c0_seq1 | 97.462 | 591 | 14 | 1 | 1 | 591 | 2176 | 407 | 0 | 1065 |
| RhMLO5 | 15GT_comp169564_c0_seq1 | 100 | 176 | 0 | 0 | 344 | 519 | 701 | 174 | 6.10E-103 | 308 |
| RhMLO6 | 15GT_comp31957_c0_seq1 | 93.443 | 427 | 11 | 2 | 1 | 410 | 1525 | 245 | 0 | 802 |
| RhMLO8 | 15GT_comp29182_c0_seq1 | 99.213 | 508 | 4 | 0 | 1 | 508 | 1774 | 251 | 0 | 887 |
| RhMLO9 | 15GT_comp34411_c0_seq2 | 99.11 | 562 | 5 | 0 | 1 | 562 | 2776 | 1091 | 0 | 1039 |
| RhMLO10 | 15GT_comp34989_c0_seq4 | 98.741 | 556 | 6 | 1 | 32 | 587 | 1904 | 240 | 0 | 1014 |
| RhMLO11 | 15GT_comp192446_c0_seq1 | 98.171 | 164 | 2 | 1 | 357 | 519 | 3 | 494 | 1.68E-81 | 251 |
| RhMLO12 | 15GT_comp25766_c0_seq2 | 98.884 | 448 | 2 | 1 | 1 | 448 | 4 | 1338 | 0 | 828 |
| RhMLO14 | 15GT_comp3643_c0_seq1 | 100 | 115 | 0 | 0 | 508 | 622 | 346 | 2 | 9.15E-76 | 236 |
| RhMLO16 | 15GT_comp34763_c0_seq7 | 99.275 | 552 | 4 | 0 | 1 | 552 | 507 | 2162 | 0 | 1104 |
| RhMLO18 | 15GT_comp846_c0_seq1 | 96.629 | 89 | 3 | 0 | 337 | 425 | 2 | 268 | 8.14E-56 | 181 |

**He.RSEM**

| Query | Subject | Identity % | Alignment length | mismatches | Gap opens | q.start | q.end | s.start | s.end | e-value | Bit score |
| --- | --- | --- | --- | --- | --- | --- | --- | --- | --- | --- | --- |
| RhMLO1 | 14He_comp26766_c0_seq2 | 97.631 | 591 | 13 | 1 | 1 | 591 | 2081 | 312 | 0 | 1065 |
| RhMLO4 | 14He_comp11984_c0_seq1 | 93.72 | 207 | 13 | 0 | 10 | 216 | 621 | 1 | 2.58E-128 | 372 |
| RhMLO6 | 14He_comp23410_c0_seq2 | 93.677 | 427 | 10 | 2 | 1 | 410 | 1524 | 244 | 0 | 802 |
| RhMLO8 | 14He_comp31169_c0_seq1 | 99.803 | 508 | 1 | 0 | 1 | 508 | 101 | 1624 | 0 | 895 |
| RhMLO9 | 14He_comp30056_c0_seq1 | 98.932 | 562 | 6 | 0 | 1 | 562 | 158 | 1843 | 0 | 1037 |
| RhMLO10 | 14He_comp27278_c0_seq2 | 98.561 | 556 | 7 | 1 | 32 | 587 | 261 | 1925 | 0 | 1012 |
| RhMLO11 | 14He_comp86961_c0_seq1 | 98.551 | 207 | 2 | 1 | 329 | 534 | 795 | 175 | 2.12E-115 | 342 |
| RhMLO12 | 14He_comp18292_c0_seq1 | 98.829 | 427 | 2 | 1 | 22 | 448 | 1 | 1272 | 0 | 772 |
| RhMLO14 | 14He_comp14670_c0_seq1 | 100 | 115 | 0 | 0 | 270 | 384 | 347 | 3 | 1.03E-77 | 241 |
| RhMLO16 | 14He_comp29522_c0_seq2 | 99.275 | 552 | 4 | 0 | 1 | 552 | 555 | 2210 | 0 | 1104 |
| RhMLO18 | 14He_comp117045_c0_seq1 | 91.781 | 73 | 6 | 0 | 325 | 397 | 223 | 5 | 9.77E-43 | 145 |

**JPC.RSEM**

| Query | Subject | Identity % | Alignment length | mismatches | Gap opens | q.start | q.end | s.start | s.end | e-value | Bit score |
| --- | --- | --- | --- | --- | --- | --- | --- | --- | --- | --- | --- |
| RhMLO1 | 12JPC_comp38331_c0_seq1 | 99.168 | 481 | 3 | 1 | 111 | 591 | 7 | 1446 | 0 | 931 |
| RhMLO2 | 12JPC_comp20162_c0_seq1 | 97.248 | 218 | 6 | 0 | 371 | 588 | 732 | 79 | 4.87E-144 | 416 |
| RhMLO3 | 12JPC_comp43209_c0_seq6 | 93.561 | 264 | 17 | 0 | 264 | 527 | 1 | 792 | 1.02E-149 | 434 |
| RhMLO6 | 12JPC_comp35064_c0_seq1 | 91.489 | 423 | 19 | 2 | 5 | 410 | 1 | 1269 | 0 | 778 |
| RhMLO8 | 12JPC_comp44333_c0_seq3 | 95.556 | 405 | 15 | 1 | 104 | 508 | 3 | 1208 | 0 | 706 |
| RhMLO9 | 12JPC_comp45507_c0_seq9 | 98.851 | 261 | 3 | 0 | 302 | 562 | 963 | 181 | 1.69E-165 | 473 |
| RhMLO10 | 12JPC_comp45887_c0_seq3 | 96.25 | 80 | 2 | 1 | 508 | 587 | 518 | 282 | 1.92E-44 | 156 |
| RhMLO11 | 12JPC_comp35005_c0_seq1 | 97.455 | 275 | 6 | 1 | 261 | 534 | 1003 | 179 | 5.28E-168 | 479 |
| RhMLO12 | 12JPC_comp32595_c0_seq3 | 99.373 | 319 | 2 | 0 | 130 | 448 | 1 | 957 | 0 | 628 |
| RhMLO16 | 12JPC_comp43570_c0_seq4 | 95.722 | 187 | 8 | 0 | 366 | 552 | 848 | 288 | 1.36E-116 | 347 |
| RhMLO18 | 12JPC_comp45944_c0_seq3 | 92.555 | 497 | 37 | 0 | 1 | 497 | 46 | 1536 | 0 | 870 |

**MB.RSEM**

| Query | Subject | Identity % | Alignment length | mismatches | Gap opens | q.start | q.end | s.start | s.end | e-value | Bit score |
| --- | --- | --- | --- | --- | --- | --- | --- | --- | --- | --- | --- |
| RhMLO1 | 5MB_comp29477_c0_seq2 | 97.631 | 591 | 14 | 0 | 1 | 591 | 2086 | 314 | 0 | 1071 |
| RhMLO5 | 5MB_comp21838_c0_seq1 | 93.931 | 379 | 2 | 1 | 162 | 519 | 2 | 1138 | 0 | 669 |
| RhMLO7 | 5MB_comp22338_c0_seq1 | 100 | 541 | 0 | 0 | 1 | 541 | 18 | 1640 | 0 | 1015 |
| RhMLO8 | 5MB_comp29450_c0_seq1 | 94.882 | 508 | 21 | 2 | 1 | 508 | 1760 | 252 | 0 | 867 |
| RhMLO9 | 5MB_comp32578_c1_seq2 | 97.756 | 312 | 7 | 0 | 251 | 562 | 2007 | 1072 | 0 | 580 |
| RhMLO11 | 5MB_comp12356_c0_seq1 | 95.872 | 218 | 8 | 1 | 318 | 534 | 1 | 654 | 3.30E-120 | 355 |
| RhMLO14 | 5MB_comp20663_c0_seq1 | 91.837 | 49 | 2 | 1 | 157 | 203 | 60 | 206 | 3.63E-23 | 92.8 |
| RhMLO15 | 5MB_comp93026_c0_seq1 | 100 | 123 | 0 | 0 | 318 | 440 | 397 | 29 | 4.60E-85 | 258 |
| RhMLO16 | 5MB_comp31134_c0_seq2 | 98.113 | 106 | 2 | 0 | 447 | 552 | 1 | 318 | 2.94E-54 | 182 |
| RhMLO18 | 5MB_comp21264_c0_seq3 | 96.988 | 166 | 5 | 0 | 226 | 391 | 1 | 498 | 8.23E-94 | 281 |

**MC.RSEM**

| Query | Subject | Identity % | Alignment length | mismatches | Gap opens | q.start | q.end | s.start | s.end | e-value | Bit score |
| --- | --- | --- | --- | --- | --- | --- | --- | --- | --- | --- | --- |
| RhMLO1 | 10MC_comp34452_c0_seq4 | 98 | 50 | 1 | 0 | 542 | 591 | 1 | 150 | 5.73E-27 | 105 |
| RhMLO3 | 10MC_comp22752_c0_seq1 | 96.894 | 161 | 4 | 1 | 1 | 160 | 485 | 3 | 2.33E-91 | 277 |
| RhMLO6 | 10MC_comp32350_c0_seq1 | 93.208 | 427 | 12 | 2 | 1 | 410 | 109 | 1389 | 0 | 800 |
| RhMLO7 | 10MC_comp216104_c0_seq1 | 100 | 70 | 0 | 0 | 237 | 306 | 210 | 1 | 1.34E-44 | 151 |
| RhMLO8 | 10MC_comp31996_c0_seq2 | 94.685 | 508 | 22 | 2 | 1 | 508 | 32 | 1540 | 0 | 866 |
| RhMLO9 | 10MC_comp30277_c0_seq2 | 97.253 | 182 | 5 | 0 | 381 | 562 | 1644 | 1099 | 2.68E-107 | 336 |
| RhMLO10 | 10MC_comp36247_c0_seq2 | 95.504 | 556 | 23 | 2 | 32 | 587 | 1884 | 223 | 0 | 1008 |
| RhMLO11 | 10MC_comp7998_c0_seq1 | 97.183 | 142 | 3 | 1 | 322 | 462 | 2 | 427 | 1.14E-94 | 285 |
| RhMLO14 | 10MC_comp21872_c0_seq1 | 90.84 | 262 | 2 | 1 | 363 | 624 | 1 | 720 | 1.67E-150 | 436 |
| RhMLO16 | 10MC_comp36389_c0_seq11 | 99.094 | 552 | 5 | 0 | 1 | 552 | 1955 | 300 | 0 | 1107 |
| RhMLO18 | 10MC_comp20328_c0_seq1 | 98.693 | 153 | 2 | 0 | 42 | 194 | 459 | 1 | 3.57E-106 | 312 |

**MF.RSEM**

| Query | Subject | Identity % | Alignment length | mismatches | Gap opens | q.start | q.end | s.start | s.end | e-value | Bit score |
| --- | --- | --- | --- | --- | --- | --- | --- | --- | --- | --- | --- |
| RhMLO1 | 1MF_comp34048_c0_seq1 | 100 | 591 | 0 | 0 | 1 | 591 | 150 | 1922 | 0 | 1083 |
| RhMLO3 | 1MF_comp35813_c0_seq28 | 98 | 350 | 7 | 0 | 214 | 563 | 2 | 1051 | 0 | 632 |
| RhMLO5 | 1MF_comp27426_c0_seq1 | 93.893 | 393 | 3 | 1 | 148 | 519 | 1494 | 316 | 0 | 696 |
| RhMLO7 | 1MF_comp295169_c0_seq1 | 97.468 | 79 | 2 | 0 | 48 | 126 | 1 | 237 | 6.51E-47 | 157 |
| RhMLO8 | 1MF_comp31017_c0_seq1 | 95.866 | 508 | 16 | 2 | 1 | 508 | 139 | 1647 | 0 | 875 |
| RhMLO9 | 1MF_comp29692_c0_seq1 | 99.336 | 301 | 2 | 0 | 262 | 562 | 2013 | 1111 | 0 | 564 |
| RhMLO10 | 1MF_comp21796_c0_seq1 | 98.387 | 124 | 2 | 0 | 32 | 155 | 25 | 396 | 5.80E-59 | 192 |
| RhMLO11 | 1MF_comp28902_c0_seq2 | 98.569 | 489 | 6 | 1 | 47 | 534 | 1624 | 158 | 0 | 886 |
| RhMLO12 | 1MF_comp25610_c0_seq1 | 100 | 302 | 0 | 0 | 147 | 448 | 2 | 907 | 0 | 598 |
| RhMLO15 | 1MF_comp15260_c0_seq1 | 100 | 260 | 0 | 0 | 262 | 521 | 2 | 781 | 4.97E-171 | 483 |
| RhMLO16 | 1MF_comp34025_c0_seq1 | 98.732 | 552 | 7 | 0 | 1 | 552 | 1996 | 341 | 0 | 1101 |
| RhMLO18 | 1MF_comp8650_c0_seq1 | 99.31 | 145 | 1 | 0 | 318 | 462 | 3 | 437 | 1.58E-91 | 275 |

**Ni.RSEM**

| Query | Subject | Identity % | Alignment length | mismatches | Gap opens | q.start | q.end | s.start | s.end | e-value | Bit score |
| --- | --- | --- | --- | --- | --- | --- | --- | --- | --- | --- | --- |
| RhMLO1 | 9Ni_comp30793_c0_seq1 | 97.755 | 579 | 12 | 1 | 1 | 579 | 159 | 1892 | 0 | 1042 |
| RhMLO3 | 9Ni_comp29367_c0_seq2 | 98.396 | 561 | 9 | 0 | 3 | 563 | 76 | 1758 | 0 | 1030 |
| RhMLO5 | 9Ni_comp13883_c0_seq1 | 98.901 | 182 | 2 | 0 | 329 | 510 | 3 | 548 | 3.34E-107 | 317 |
| RhMLO6 | 9Ni_comp26985_c0_seq1 | 92.037 | 427 | 17 | 2 | 1 | 410 | 92 | 1372 | 0 | 792 |
| RhMLO8 | 9Ni_comp27228_c0_seq1 | 99.409 | 508 | 3 | 0 | 1 | 508 | 57 | 1580 | 0 | 893 |
| RhMLO9 | 9Ni_comp27896_c0_seq2 | 99.822 | 562 | 1 | 0 | 1 | 562 | 2787 | 1102 | 0 | 1048 |
| RhMLO10 | 9Ni_comp29428_c0_seq2 | 99.281 | 556 | 4 | 0 | 32 | 587 | 104 | 1771 | 0 | 1024 |
| RhMLO11 | 9Ni_comp17527_c1_seq1 | 98.23 | 226 | 3 | 1 | 310 | 534 | 1 | 678 | 1.80E-128 | 377 |
| RhMLO12 | 9Ni_comp19315_c0_seq1 | 98.42 | 443 | 4 | 1 | 6 | 448 | 1 | 1320 | 0 | 800 |
| RhMLO14 | 9Ni_comp31912_c1_seq1 | 94 | 100 | 4 | 1 | 525 | 624 | 377 | 84 | 2.76E-52 | 191 |
| RhMLO15 | 9Ni_comp4499_c0_seq1 | 100 | 204 | 0 | 0 | 292 | 495 | 612 | 1 | 1.10E-124 | 362 |
| RhMLO16 | 9Ni_comp28460_c0_seq14 | 99.275 | 552 | 4 | 0 | 1 | 552 | 784 | 2439 | 0 | 1104 |
| RhMLO18 | 9Ni_comp18956_c0_seq1 | 98.684 | 152 | 2 | 0 | 276 | 427 | 65 | 520 | 2.70E-81 | 249 |

**P540.RSEM**

| Query | Subject | Identity % | Alignment length | mismatches | Gap opens | q.start | q.end | s.start | s.end | e-value | Bit score |
| --- | --- | --- | --- | --- | --- | --- | --- | --- | --- | --- | --- |
| RhMLO1 | P540_comp5971_c0_seq1 | 100 | 81 | 0 | 0 | 482 | 562 | 245 | 3 | 2.79E-51 | 170 |
| RhMLO8 | P540_comp33513_c0_seq1 | 99.604 | 505 | 2 | 0 | 1 | 505 | 41 | 1555 | 0 | 883 |
| RhMLO9 | P540_comp25298_c0_seq1 | 99.074 | 216 | 2 | 0 | 330 | 545 | 1 | 648 | 4.94E-134 | 388 |
| RhMLO10 | P540_comp53825_c0_seq1 | 99.231 | 130 | 1 | 0 | 456 | 585 | 2 | 391 | 6.69E-66 | 210 |
| RhMLO16 | P540_comp28352_c0_seq2 | 99.32 | 294 | 2 | 0 | 259 | 552 | 1102 | 221 | 0 | 576 |
| RhMLO18 | P540_comp83440_c0_seq1 | 99.16 | 119 | 1 | 0 | 15 | 133 | 1 | 357 | 1.53E-80 | 245 |

**P867.RSEM**

| Query | Subject | Identity % | Alignment length | mismatches | Gap opens | q.start | q.end | s.start | s.end | e-value | Bit score |
| --- | --- | --- | --- | --- | --- | --- | --- | --- | --- | --- | --- |
| RhMLO1 | P867_comp1324_c0_seq1 | 98 | 100 | 2 | 0 | 371 | 470 | 300 | 1 | 1.00E-52 | 174 |
| RhMLO8 | P867_comp32904_c0_seq1 | 100 | 474 | 0 | 0 | 35 | 508 | 1628 | 207 | 0 | 852 |
| RhMLO9 | P867_comp32566_c0_seq1 | 99.088 | 548 | 5 | 0 | 1 | 548 | 1645 | 2 | 0 | 1011 |
| RhMLO10 | P867_comp25545_c0_seq2 | 99.057 | 212 | 2 | 0 | 376 | 587 | 667 | 32 | 1.90E-131 | 382 |
| RhMLO16 | P867_comp25098_c0_seq1 | 99.303 | 287 | 2 | 0 | 1 | 287 | 59 | 919 | 0 | 593 |
| RhMLO18 | P867_comp56630_c0_seq1 | 100 | 101 | 0 | 0 | 18 | 118 | 1 | 303 | 6.65E-67 | 209 |

**PJ.RSEM**

| Query | Subject | Identity % | Alignment length | mismatches | Gap opens | q.start | q.end | s.start | s.end | e-value | Bit score |
| --- | --- | --- | --- | --- | --- | --- | --- | --- | --- | --- | --- |
| RhMLO1 | 3PJ_comp34214_c0_seq1 | 99.831 | 591 | 1 | 0 | 1 | 591 | 2129 | 357 | 0 | 1082 |
| RhMLO3 | 3PJ_comp33095_c0_seq1 | 96.552 | 232 | 8 | 0 | 207 | 438 | 1 | 696 | 8.28E-150 | 429 |
| RhMLO5 | 3PJ_comp21807_c1_seq2 | 99.394 | 330 | 2 | 0 | 190 | 519 | 3 | 992 | 0 | 624 |
| RhMLO6 | 3PJ_comp26129_c0_seq1 | 92.974 | 427 | 13 | 2 | 1 | 410 | 1518 | 238 | 0 | 799 |
| RhMLO7 | 3PJ_comp24052_c0_seq2 | 100 | 235 | 0 | 0 | 307 | 541 | 730 | 26 | 3.34E-146 | 419 |
| RhMLO8 | 3PJ_comp33138_c0_seq1 | 95.079 | 508 | 20 | 2 | 1 | 508 | 1759 | 251 | 0 | 867 |
| RhMLO9 | 3PJ_comp33277_c0_seq1 | 99.11 | 562 | 5 | 0 | 1 | 562 | 172 | 1857 | 0 | 1039 |
| RhMLO10 | 3PJ_comp34253_c0_seq2 | 99.46 | 556 | 3 | 0 | 32 | 587 | 1823 | 156 | 0 | 1025 |
| RhMLO11 | 3PJ_comp18195_c0_seq1 | 96.97 | 132 | 3 | 1 | 404 | 534 | 3 | 398 | 1.76E-56 | 186 |
| RhMLO12 | 3PJ_comp25388_c1_seq1 | 98.884 | 448 | 2 | 1 | 1 | 448 | 147 | 1481 | 0 | 828 |
| RhMLO15 | 3PJ_comp4805_c0_seq1 | 100 | 125 | 0 | 0 | 316 | 440 | 399 | 25 | 2.63E-86 | 261 |
| RhMLO16 | 3PJ_comp34743_c0_seq3 | 98.732 | 552 | 7 | 0 | 1 | 552 | 809 | 2464 | 0 | 1103 |
| RhMLO18 | 3PJ_comp20442_c0_seq1 | 97.778 | 180 | 4 | 0 | 317 | 496 | 642 | 103 | 1.52E-115 | 338 |

**RND.RSEM**

| Query | Subject | Identity % | Alignment length | mismatches | Gap opens | q.start | q.end | s.start | s.end | e-value | Bit score |
| --- | --- | --- | --- | --- | --- | --- | --- | --- | --- | --- | --- |
| RhMLO1 | 11RND_comp33556_c0_seq1 | 98.986 | 592 | 5 | 1 | 1 | 591 | 2087 | 312 | 0 | 1077 |
| RhMLO3 | 11RND_comp31928_c2_seq2 | 93.312 | 314 | 20 | 1 | 215 | 527 | 1232 | 291 | 0 | 524 |
| RhMLO6 | 11RND_comp30438_c0_seq1 | 92.037 | 427 | 17 | 2 | 1 | 410 | 1511 | 231 | 0 | 792 |
| RhMLO8 | 11RND_comp35576_c0_seq8 | 100 | 212 | 0 | 0 | 297 | 508 | 725 | 90 | 2.69E-126 | 367 |
| RhMLO9 | 11RND_comp35801_c0_seq2 | 99.644 | 562 | 2 | 0 | 1 | 562 | 2726 | 1041 | 0 | 1046 |
| RhMLO10 | 11RND_comp32292_c0_seq1 | 98.561 | 556 | 8 | 0 | 32 | 587 | 287 | 1954 | 0 | 1018 |
| RhMLO11 | 11RND_comp13411_c0_seq1 | 98.826 | 426 | 4 | 1 | 110 | 534 | 2 | 1279 | 0 | 793 |
| RhMLO12 | 11RND_comp28073_c0_seq1 | 96.018 | 452 | 11 | 2 | 1 | 448 | 162 | 1508 | 0 | 796 |
| RhMLO13 | 11RND_comp114590_c0_seq1 | 98.701 | 77 | 1 | 0 | 546 | 622 | 1 | 231 | 1.00E-32 | 120 |
| RhMLO15 | 11RND_comp139814_c0_seq1 | 99.338 | 151 | 1 | 0 | 290 | 440 | 477 | 25 | 9.12E-106 | 312 |
| RhMLO16 | 11RND_comp35191_c0_seq9 | 99.094 | 552 | 5 | 0 | 1 | 552 | 508 | 2163 | 0 | 1102 |
| RhMLO18 | 11RND_comp16597_c0_seq1 | 99.213 | 127 | 1 | 0 | 8 | 134 | 3 | 383 | 1.54E-86 | 261 |

**Rh88.RSEM**

| Query | Subject | Identity % | Alignment length | mismatches | Gap opens | q.start | q.end | s.start | s.end | e-value | Bit score |
| --- | --- | --- | --- | --- | --- | --- | --- | --- | --- | --- | --- |
| RhMLO1 | UN043617 | 98.413 | 252 | 4 | 0 | 233 | 484 | 70 | 825 | 0 | 459 |
| RhMLO1 | UN043617 | 100 | 85 | 0 | 0 | 507 | 591 | 894 | 1148 | 0 | 180 |
| RhMLO1 | UN043617 | 96.429 | 28 | 1 | 0 | 211 | 238 | 3 | 86 | 0 | 63.2 |
| RhMLO1 | UN043617 | 91.667 | 24 | 2 | 0 | 485 | 508 | 827 | 898 | 0 | 48.9 |
| RhMLO2 | UN107852 | 98.361 | 244 | 4 | 0 | 345 | 588 | 17 | 748 | 7.95E-166 | 473 |
| RhMLO3 | UN000484 | 98.551 | 138 | 2 | 0 | 254 | 391 | 807 | 1220 | 0 | 249 |
| RhMLO3 | UN000484 | 100 | 113 | 0 | 0 | 141 | 253 | 469 | 807 | 0 | 240 |
| RhMLO3 | UN000484 | 96 | 100 | 4 | 0 | 42 | 141 | 171 | 470 | 0 | 160 |
| RhMLO3 | UN000484 | 97.727 | 44 | 1 | 0 | 1 | 44 | 47 | 178 | 0 | 90.1 |
| RhMLO4 | UN101175 | 95.192 | 104 | 5 | 0 | 261 | 364 | 1 | 312 | 2.94E-62 | 201 |
| RhMLO4 | UN101175 | 100 | 30 | 0 | 0 | 221 | 250 | 360 | 449 | 1.12E-13 | 66.2 |
| RhMLO4 | UN101175 | 91.667 | 12 | 1 | 0 | 209 | 220 | 325 | 360 | 1.12E-13 | 28.5 |
| RhMLO5 | UN036317 | 99.057 | 318 | 3 | 0 | 202 | 519 | 94 | 1047 | 0 | 595 |
| RhMLO7 | UN040767 | 94.017 | 117 | 7 | 0 | 1 | 117 | 154 | 504 | 4.59E-160 | 227 |
| RhMLO7 | UN040767 | 99.13 | 115 | 1 | 0 | 113 | 227 | 491 | 835 | 4.59E-160 | 179 |
| RhMLO7 | UN040767 | 97.561 | 41 | 1 | 0 | 334 | 374 | 1154 | 1276 | 1.41E-18 | 88.6 |
| RhMLO8 | UN016129 | 98.958 | 96 | 1 | 0 | 403 | 498 | 1 | 288 | 2.72E-35 | 128 |
| RhMLO9 | UN040269 | 97.857 | 140 | 3 | 0 | 423 | 562 | 1190 | 771 | 2.45E-78 | 255 |
| RhMLO10 | UN017519 | 96.639 | 119 | 3 | 1 | 469 | 587 | 217 | 570 | 2.66E-84 | 177 |
| RhMLO10 | UN017519 | 96.053 | 76 | 3 | 0 | 398 | 473 | 3 | 230 | 2.66E-84 | 154 |
| RhMLO11 | UN105508 | 96.667 | 30 | 1 | 0 | 504 | 533 | 2 | 91 | 4.86E-11 | 60.1 |
| RhMLO12 | UN091166 | 90.909 | 55 | 5 | 0 | 147 | 201 | 24 | 188 | 3.67E-27 | 107 |
| RhMLO13 | UN109686 | 90 | 10 | 1 | 0 | 309 | 318 | 570 | 599 | 3.34E-22 | 21.6 |
| RhMLO14 | UN042249 | 98.718 | 78 | 1 | 0 | 517 | 594 | 232 | 465 | 7.49E-53 | 165 |
| RhMLO14 | UN042249 | 100 | 31 | 0 | 0 | 594 | 624 | 464 | 556 | 7.49E-53 | 62 |
| RhMLO15 | UN004772 | 100 | 85 | 0 | 0 | 409 | 493 | 2 | 256 | 1.27E-43 | 135 |
| RhMLO16 | UN083783 | 100 | 100 | 0 | 0 | 63 | 162 | 1 | 300 | 7.08E-66 | 210 |
| RhMLO18 | UN084790 | 91.667 | 96 | 1 | 1 | 1 | 96 | 58 | 324 | 5.78E-52 | 172 |
