# Supplementary material for: Analysis of allelic variants of RhMLO genes in rose and functional studies on susceptibility to powdery mildew related to clade V homologs
Source: Theor Appl Genet. 2021 May 2;134(8):2495–515. doi: 10.1007/s00122-021-03838-7 (PMC8277636; doi:10.1007/s00122-021-03838-7)
Supplement: Supplementary file 15 — Supplementary file15 (DOCX 23 KB) [file 122_2021_3838_MOESM15_ESM.docx]

**Supplementary Table S3. The number of times sequences were found in different rose samples**

| **Gene name** | **All rose samples**  **(n=22)** | **Wild rose species**  **(n=8)** | **Garden rose**  **(n=11)** | **Cut rose**  **(n=2)** | **Rh88**  **(n=1)** |
| --- | --- | --- | --- | --- | --- |
| ***RhMLO1*** | 23 | 9 | 11 | 2 | 1 |
| ***RhMLO2*** | 11 | 8 | 2 | 0 | 1 |
| ***RhMLO3*** | 17 | 8 | 8 | 0 | 1 |
| ***RhMLO4*** | 6 | 4 | 1 | 0 | 1 |
| ***RhMLO5*** | 16 | 9 | 6 | 0 | 1 |
| ***RhMLO6*** | 17 | 8 | 9 | 0 | 0 |
| ***RhMLO7*** | 13 | 8 | 4 | 0 | 1 |
| ***RhMLO8*** | 22 | 8 | 11 | 2 | 1 |
| ***RhMLO9*** | 22 | 8 | 11 | 2 | 1 |
| ***RhMLO10*** | 22 | 9 | 10 | 2 | 1 |
| ***RhMLO11*** | 20 | 8 | 11 | 0 | 1 |
| ***RhMLO12*** | 18 | 8 | 9 | 0 | 1 |
| ***RhMLO13*** | 13 | 12 | 1 | 0 | 0 |
| ***RhMLO14*** | 14 | 7 | 6 | 0 | 1 |
| ***RhMLO15*** | 14 | 8 | 5 | 0 | 1 |
| ***RhMLO16*** | 22 | 8 | 11 | 2 | 1 |
| ***RhMLO17*** | 8 | 8 | 0 | 0 | 0 |
| ***RhMLO18*** | 22 | 8 | 11 | 2 | 1 |
| ***RhMLO19*** | 0 | 0 | 0 | 0 | 0 |
| **Total** | 300 | 146 | 127 | 12 | 15 |
