# Supplementary material for: Analysis of allelic variants of RhMLO genes in rose and functional studies on susceptibility to powdery mildew related to clade V homologs
Source: Theor Appl Genet. 2021 May 2;134(8):2495–515. doi: 10.1007/s00122-021-03838-7 (PMC8277636; doi:10.1007/s00122-021-03838-7)
Supplement: Supplementary file 16 — Supplementary file16 (DOCX 43 KB) [file 122_2021_3838_MOESM16_ESM.docx]

**Table S3 Overview of the alleles based on the SNPs in coding region**

|  | **Unique Allele(H)** | **Sequence of concatenated SNPs^#^** | **Species or varieties (followed by a number,**  **if more than one allele was found for an accession)** |
| --- | --- | --- | --- |
| ***RhMLO1*** | 1 | ACATATAAAAACGAAGAGCCTGACG | AH, MF, OB1-genome, *R.chinensis* |
|  | 2 | TATGTACGCCAGGCTGTTCCACCCG | GT, He |
|  | 3 | TATGTACGCCGGGCTAATCCACCCG | MB, *R.rugosa-2* |
|  | 4 | TATGTACGCCAGGCTGTTCCACACG | Ni |
|  | 5 | ACATATAAAAACGAAGAGCCAGACG | PJ |
|  | 6 | ACATAACAAAACGCTGATTGACCTA | *R.laevigata* |
|  | 7 | ACATAACGAAACACTGATCCACCCG | *R.minutifolia* |
|  | 8 | TATGTACGAAACACTGATCCACCCG | *R.moschata* |
|  | 9 | ACATAACAAAACGCTGAGCGACCTA | *R.xanthina* |
|  | 10 | ACATATAAAAACGATGAGCCAGACG | RND |
|  | 11 | TATGTAAAAAACGATGAGNNNNNNN | *R.rugosa*-1 |
|  | 12 | NNNNNNNNNNNNNNNNNNTCAGACG | DB |
|  | 13 | NNNNNNNNNNNNNNNNNNNCACCTG | *R.persica* |
| ***RhMLO2*** | 1 | CGAGGCGCAAACACGGG | OB2-genome, *R.chinensis* |
|  | 2 | TAAGGTGCCGATAGAAC | *R.persica* |
|  | 3 | TAGAATGCCGATGGAAC | *R.laevigata* |
|  | 4 | CAAGGCTTCGATGGAAC | *R.minutifolia* |
|  | 5 | TAGAACTTCAATGGAAC | *R.moschata* |
|  | 6 | TAAGGCGCCGGTAGAGC | *R.xanthina*, JPC |
|  | 7 | CGAGGCGCCAATGGNNN | *R.rugosa* |
|  | 8 | NNNNNNNNNGATAGAGG | Rh88 |
| ***RhMLO3*** | 1 | ACCCAAGGGTGATTTAGCCGGGTAGACGCCAACGAGACC | OB3-genome |
|  | 2 | ACCTAGGGGTGATTTAGCCGGGTAGACGCCAACGAGACC | Ni, Rh88 |
|  | 3 | AGATNNGGGTGACCAAGCCGGCTAAACCCCAACGAGAAC | *R. persica* |
|  | 4 | CGNTTGAAAACACTTTGTCACCGCAACCCGAGCGAGACC | *R.minutifolia* |
|  | 5 | AGATTGGGGTGACCAAGCCGGCGAAACCACAACGACACC | *R.rugosa*-1 |
|  | 6 | NCNTAGGANGCACTTTGTAGGGTCAACCCGAGCGAGACC | *R.rugosa*-2 |
|  | 7 | CGATAGAGGTGACTTAGTCACCGCAACCCCAACGAGCCC | *R.xanthina* |
|  | 8 | NNNNNNNGGTGGCTAAACGGGCGCAGTCCCGAAAGGCAA | RND, JPC |
|  | 9 | NNNNNNNGGTGATTTAGCCGGGTAGACCACAACGACACC | DB |
|  | 10 | NNNNNNNGGTCACTTAGCCGGGTAGACCCCAGCGAGACC | MF |
|  | 11 | NNNNNNNGGTCACTTAGTCACAGCAACNNNNNNNNNNNN | PJ |
|  | 12 | NNNNNAGGGTGACTTAGNNNNNNNNNNNNNNNNNNNNNN | OB-transcriptome |
|  | 13 | NNNNNNNAAACACTAAGNNNNNNNNNNNNNNNNNNNNNN | AH |
|  | 14 | ACCCAGGNNNNNNNNNNNNNNNNNNNNNNNNNNNNNNNN | *R.chinensis* |
|  | 15 | ACCCAGANNNNNNNNNNNNNNNNNNNNNNNNNNNNNNNN | MC |
| ***RhMLO4*** | 1 | AGGAGGCACGTATGC | OB4-genome, *R.chinensis* |
|  | 2 | GACGCAAGAGCTCCA | *R.rugosa* ,He |
|  | 3 | NNNNNNNNNATATCA | OB-transcriptome |
|  | 4 | NNNNNACGCACNNNN | *R.moschata* |
|  | 5 | NNNNNNNNNATTCCT | Rh88 |
| ***RhMLO5*** | 1 | TCCNGC | OB5-genome, *R.chinensis*, GT |
|  | 2 | TTTANA | *R.xanthina*, *R. persica* |
|  | 3 | TCTANA | *R.laevigata* |
|  | 4 | CCTCNA | *R.rugosa*-2 |
|  | 5 | TCCCNA | *R.rugosa*-1 |
|  | 6 | CCTANN | *R.moschata*-1a |
|  | 7 | NNNCAA | MF, MB, PJ, AH, Ni |
|  | 8 | NNNNGA | Rh88 |
| ***RhMLO6*** | 1 | AGGATTAGACGT | DB, He, OB6-genome, *R.chinensis* |
|  | 2 | AGGATTAAAACT | AH, PJ, *R.moschata* |
|  | 3 | ACGATTAGACGT | GT |
|  | 4 | AGTTACAACACC | JPC |
|  | 5 | AGGATTAAACCT | MC |
|  | 6 | GCGTACGAACCT | Ni, RND |
|  | 7 | AGGTACNACACC | *R. persica* |
|  | 8 | AGGTACAAAACT | *R.laevigata* |
|  | 9 | AGGATTNAAACT | *R.minutifolia* |
|  | 10 | AGGATTGAACCT | *R.rugosa* |
|  | 11 | AGTTACNACACC | *R.xanthina* |
|  | 12 | GGNNNNNNNNNN | OB-transcriptome |
| ***RhMLO7*** | 1 | GCGTCGACTCCAAAGGGC | MB, OB7-genome, *R.chinensis*, PJ |
|  | 2 | GTTTTGGACGAGCGAAAC | *R. persica* |
|  | 3 | GCTTTGGCCCCGCAGGAC | *R.laevigata* |
|  | 4 | CCTCTGGCCCCGCGGGTT | *R.moschata* |
|  | 5 | NNTTTGGACGAGCGAAAT | *R.xanthina* |
|  | 6 | GTGTTAACTCCAAANNNN | Rh88, MF |
|  | 7 | CCTCTGGCCCCAANNNNN | *R.rugosa* |
|  | 8 | NNNNNNNNNNNNCGGGAT | *R.minutifolia* |

**Table S3 Overview of the alleles based on the SNPs in coding region (continued)**

|  | **Unique Allele(H)** | **Sequence of concatenated SNPs^#^** | | **Species or varieties(followed by a number, if more than one allele was found)** |
| --- | --- | --- | --- | --- |
| ***RhMLO8*** | 1 | GAGGAGGCCGGACCCGTGTAACG | | OB8-genome,P867, *R.chinensis* |
|  | 2 | GAGAAAGCAATAAAAGTCCGACG | | AH |
|  | 3 | GAAGAGGCCGGAACCGTGTAACG | | DB |
|  | 4 | GAAGGGGCCGGAACCGTGTAACG | | GT |
|  | 5 | GAAGAGGCCGGACCCGTGTAACG | | He, OB-transcriptome |
|  | 6 | AAGGAGGCAGGAAAAATGCTTAG | | MB,MC |
|  | 7 | GAGGAGGCCGGACCCGTCTTTAG | | MF |
|  | 8 | GAAGAGGCCGGACCCGTCTAACG | | Ni,Rh88 |
|  | 9 | GAGGAGGCCGGACCCGTGTTTAG | | P540 |
|  | 10 | AAGGAGGCCGGAAAAATGCTTAG | | PJ |
|  | 11 | GCGAANGCAATAACCGGGCAACG | | *R. persica* |
|  | 12 | GCGGAGGCAAGTAAAGGGTAACC | | *R.laevigata* |
|  | 13 | GAAGAGGCAGGAAAAATGCTTAG | | *R.minutifolia*, *R.rugosa* |
|  | 14 | GAGGAGAAAGGTAAAATGCTTAG | | *R.moschata* |
|  | 15 | GCGAGAAAAATAAAAGGGTAACC | | *R.xanthina* |
|  | 16 | NNNNAGGCCGGAAAAGTGTAACG | | JPC |
| ***RhMLO9*** | 1 | CACAAAGAAAAGCTGGA | | Ni, OB9-genome |
|  | 2 | CACAAGGACAAGTTGGA | | AH, DB,Rh88 |
|  | 3 | CACAAAGAATGACCGGA | | GT, PJ, P867, MC |
|  | 4 | CACAAGGAATGACCGGA | | He |
|  | 5 | GATAGNAAATAGCCAGG | | *R.persica* |
|  | 6 | CACAANGAAAAGCTGGA | | *R.chinensis* |
|  | 7 | CGTAANAAATAGCCAGA | | *R.laevigata* |
|  | 8 | CACAAAGCATAGCCGAA | | *R.minutifolia*-1a |
|  | 9 | CACGANNACAAGTTGGA | | *R.rugosa* |
|  | 10 | GGCAGAGAATAGCTGGG | | *R.xanthina* |
|  | 11 | CACAAAGAAAAGTTGGA | | RND, MF, P540 |
|  | 12 | NACGAAGCATAGCCGAA | | *R.moschata* |
|  | 13 | NNNNNNGAATAGCCGGA | | JPC |
|  | 14 | NNNNNGGACTAGTTGGA | | MB |
| ***RhMLO10*** | 1 | GCAAAAGACAGGACT | | OB10-genome |
|  | 2 | GCAAGTTATAGAGCA | | AH, RND |
|  | 3 | GCAAGAGATCGAACT | | DB |
|  | 4 | GCAAGTTATAGGACA | | GT, P867 |
|  | 5 | GCAAGTTATAGAACA | | He, OB-transcriptome, Rh88 |
|  | 6 | GCGGAAGATCGGAAT | | MC |
|  | 7 | GCAAGATACCGGACT | | Ni |
|  | 8 | GCGGAAGACAGGACT | | PJ, *R.chinensis* |
|  | 9 | AGNNNNNGTAGGAAT | | *R. persica* |
|  | 10 | GCGGAAGATAGGAAT | | *R.laevigata* |
|  | 11 | GCGGAAGGCAGGAAT | | *R.minutifolia* |
|  | 12 | GCGGAAGGTCAGAAT | | *R.moschata* |
|  | 13 | GCAAGTTNNNAGACA | | *R.rugosa*-1 |
|  | 14 | GCAAGTTNNNAGAAT | | *R.rugosa*-2 |
|  | 15 | AGGGAAGGTCGGAAT | | *R.xanthina* |
| ***RhMLO11*** | 1 | CGTTTGGTAATCGTGACAA | OB11-genome | |
|  | 2 | TGTATAGTAAGCGTGAATC | MF | |
|  | 3 | TTAAAGGCAAGAGTAAATC | *R. persica* | |
|  | 4 | CGTTTAGTAATCGTGACAA | *R.chinensis* | |
|  | 5 | CGTAAAATAAGAGAAAATC | *R.laevigata* | |
|  | 6 | TGTAAAGCGAGACAATATC | *R.minutifolia* | |
|  | 7 | TTAATAGCGAGACAAAATC | *R.moschata* | |
|  | 8 | TGTATAGTAAGACAATATC | *R.rugosa* | |
|  | 9 | NGTTTAGTAAGCGTGAATC | RND | |
|  | 10 | NNNNAAGTAAGCGTGAATC | JPC | |
|  | 11 | NNNNNNACGAGCGTGAATC | AH | |
|  | 12 | NNNNNNGCGCGACAATATC | MB,MC | |
|  | 13 | NNNNNNNTACGCGTGAATC | PJ | |
|  | 14 | TTAAAAGNNNNNNNNNNNN | *R.xanthina* | |
| ***RhMLO12*** | 1 | GAAAGCCCGAAA | OB12-genome | |
|  | 2 | ACATGCAACAAA | AH | |
|  | 3 | GAAAGCCCCAAA | GT, He, PJ | |
|  | 4 | AAAAGCAACAAA | Ni | |
|  | 5 | ACGTGGAAGAAA | *R.chinensis* | |
|  | 6 | GCGAACCANAAA | *R.laevigata* | |
|  | 7 | GCGAGCCAGAAA | *R.minutifolia* | |
|  | 8 | GCGCAGAAGAGT | *R.xanthina*, *R.persica* | |
|  | 9 | ACGTGGAACAAA | RND | |
|  | 10 | GCATGCAAGTNN | *R.rugosa* | |
|  | 11 | NNNNNNNNNTAA | *R.moschata* | |

**Table S3 Overview of the alleles based on the SNPs in coding region (continued)**

|  | **Unique Allele**  **(H)** | **Sequence of concatenated SNPs^#^** | | **Species or varieties**  **(followed by a number,**  **if more than one allele**  **was found)** |
| --- | --- | --- | --- | --- |
| ***RhMLO13*** | 1 | TTTCGCCGATTAAACATGAATAGTTTTCACGAGTGCAAGAGGACATTACACTTTAGCCGACAGATCCTTACGATAGA | | OB13-genome |
|  | 2 | TTCTCCCGATTNNNNNTGAATAGNNNNCACGAGTGCAAGAGGACATTACACTTTAGCCGACAGATCCTTACGATAGA | | *R.chinensis* |
|  | 3 | TTCTCCCGATTNNNNNNNNNNNNNNNNNNCGAAAGCAAGCGGACATTAAACTCTAGCCGACAGATCCCTACGATAGA | | *R.laevigata*-1 |
|  | 4 | NTTCGGACTATTGTGTCAATGCATCCGGCTAGGTAAGGAAAAGGCGGTATACCTGCGGCGCTATAAACCCGCTNNNN | | *R.laevigata*-2 |
|  | 5 | AGCTCCCGATGAAACATGGATAGNNNNCACGAAAGTAAGCNNNNNNNAAACTCTAGCCGACAGATCCCTACGATAGA | | *R.minutifolia*-1 |
|  | 6 | NNNNNNNNNNNNNTGTCAATGCACCCGGCTAGGTAAGGAAAAGGCGGCATACCCGCGGCGCTATAAACCCGCTGCCG | | *R.minutifolia*-2 |
|  | 7 | NNNNNNNNNNNNNNNNNNNNNNNNNNNNNNNNNNNNNNNNGGACATTAAACCCTAGCCCACTGATCCCTACAATAGA | | *R.persica* |
|  | 8 | AGCTCCCGATGAAACATGGATAGNNNNCACGAAAGTAAGCNNNNNNNAAACTCTAGCCGAAAGATCCCTACGATAGA | | *R.moschata*-1 |
|  | 9 | NTTCGGACTATTGTGTCGATGCACCCGGCTAGGTAAGGAAAAGGCGGCATACCCGCGGCGCTATAAACCCGCTGCCG | | *R.moschata*-2 |
|  | 10 | NNNNNNNNNNNNNNNNNNNNNNNNNNNNNCGAAAGTAAGCGGACATTAAACTCTAGCCGAAAGATCCCTACGATAGA | | *R.rugosa*-1 |
|  | 11 | NNNNNNNNNNNNNNNNNNNNNNNNNNNNNCGAAAGTAAGCGGACATTACACTTTAGCCGAAAGATCCCTACGATAGA | | *R.rugosa*-2 |
|  | 12 | NNNNNNNNNNNNNNNNNNNNNNNNNNNNNNNNNNNNNNNNNNNNCGGCATACCTGCGGCGCTATAAACCCGCTGCCG | | *R.rugosa*-3 |
|  | 13 | TTTTCCAGATTAGACATGAATAGTTTTCACGAGTGCGAGAGGACATTAAACTCTAGCCCACTGATCCCTAGGTTAGA | | *R.xanthina* |
| ***RhMLO14*** | 1 | TGTTGAAAGACCTT | | OB14 genome |
|  | 2 | TGTTGAAGGACCTT | | *R.chinensis* |
|  | 3 | AGTTGGCATTGTAC | | *R.minutifolia*, *R.moschata* |
|  | 4 | TACCCGAAGAGTNC | | *R. persica* |
|  | 5 | AACGCGAGGAGTAC | | *R.xanthina* |
|  | 6 | NNTTGGAATTGTNN | | *R.rugosa* |
|  | 7 | NNNNNNNAGTGTNN | | MB |
| ***RhMLO15*** | 1 | TAAATA | | OB15-genome,*R.chinensis*,  OB-transcriptome ,MF |
|  | 2 | TATATC | | *R.minutifolia* |
|  | 3 | TGCATC | | *R.xanthina*, *R. persica* |
|  | 4 | GACATC | | *R.moschata* |
|  | 5 | TACATC | | *R.laevigata* |
|  | 6 | GAAGAC | | *R.rugosa* |
|  | 7 | NNAGAA | | Rh88 |
| ***RhMLO16*** | 1 | GCGAACCTAG | | OB16-genome |
|  | 2 | ACAAACCTAT | | DB, GT, He, Ni ,AH,MB,  P540 |
|  | 3 | ACAAACATCG | | MC |
|  | 4 | AAAAACATCG | | MF, PJ |
|  | 5 | GGAGCTACCG | | *R. persica* |
|  | 6 | GCNAACCTAG | | *R.chinensis* |
|  | 7 | AANAACATCG | | *R.minutifolia*, *R.moschata* |
|  | 8 | GAGGCTACCG | | *R.xanthina* |
|  | 9 | ACAAACCTCT | | RND |
|  | 10 | NNNNNTATCG | | JPC |
| ***RhMLO17*** | 1 | CGGGTGCG | | OB17-genome, *R.chinensis* |
|  | 2 | GGGGCAGG | | *R.laevigata* |
|  | 3 | GAGTTACC | | *R.moschata* |
|  | 4 | CAATCAGC | | *R.minutifolia* |
|  | 5 | CAAGNAGC | | *R. persica* |
|  | 6 | CAAGCAGG | | *R.xanthina* |
| ***RhMLO18*** | 1 | | GCCCGGGAAGACGGCGAAGAGGCGGCAGAAAGGTAGGACGTGAGATAAGGCTGAAAGAGAGTT | OB18-genome |
|  | 2 | | GCCCGGGAAGACGGCGAAGAGGCGGCAGAAAGGTAGGACGTGAGATAAGGCTGAAAGAAAGTT | *R.chinensis* |
|  | 3 | | ACAGAAATGGANCGCAAGACAAATCTTAAATGGTTGGCGGTAAGAAAAGGCTGGCACAAGGTT | *R.laevigata* |
|  | 4 | | ACAAAAATGGAGCGCAAGACAACTCTTAAATGGTTGGACGNNAGAAAGGACTGGCGGAAGGTT | *R.minutifolia* |
|  | 5 | | AGAAAGGAAGAGCAAGTAACAACTCTAGGATGGTTAGCCGTGCAATAGGACTTGCAAAAGGTT | *R.moschata* |
|  | 6 | | GGAAAAGTAGCCCAAAAAGCGGCGGCAGGATGGTTAGCCGTGCAATAGGACTTGCAAAAGGTT | *R.rugosa* |
|  | 7 | | GCCAGAGTAGCGCGCGAAACAACTCTTGGAACACTGCTTGGAAGGTGAAATAGGCGGAGGTCC | *R.xanthina* |
|  | 8 | | GCCCGGGAAAACGGCGTAACAACTCTTGAATCACTGCTTGGGAGCTGGAATAGACGGAGGTCC | JPC |
|  | 9 | | NNNNNGGTCAAGCGCGAAACAAATCCTGAATCACTGGTTGGAAGCAGGAATAGACGGAGCTCC | *R.persica* |
|  | 10 | | NNNNNNNNNNNNNNNNNNNCGGCGGCAGAGAGGTAGGACGTGAGATAAGGCTGAAAGAAGGTT | PJ |
|  | 11 | | NNNNNNNNNNNNNNNNNNNNGGCGGCAGAGAGGTAGGACGTGAGATAAGGCTGAAAGAGANNN | MF |
|  | 12 | | NNNNNNNNNNNNNNNNNNNNNNNGGCAGAAAGGTAGGACATGAAATAAGGCTGAAAGGAGGTT | AH, DB |
|  | 13 | | NNNNNNNNNNNNNNNNNNNCGGCGGCAGAAAGGTTGNNNNNNNNNNNNNNNNNNNNNNNNNNN | Ni |
|  | 14 | | NNNNNNNNNNNNNNNGTAACGGCGGCAGNNNNNNNNNNNNNNNNNNNNNNNNNNNNNNNNNNN | MB |
|  | 15 | | NNNNNNNNNNNNNNNNNNNNNNNNNTTGAAAGGTTNNNNNNNNNNNNNNNNNNNNNNNNNNNN | GT |
|  | 16 | | NNNNNNNNNNNNNNNNNNNNNACTCTTGGATNNNNNNNNNNNNNNNNNNNNNNNNNNNNNNNN | He |

^#^For SNP and indel positions, see supplementary excel.
