# Supplementary material for: Analysis of allelic variants of RhMLO genes in rose and functional studies on susceptibility to powdery mildew related to clade V homologs
Source: Theor Appl Genet. 2021 May 2;134(8):2495–515. doi: 10.1007/s00122-021-03838-7 (PMC8277636; doi:10.1007/s00122-021-03838-7)
Supplement: Supplementary file 17 — Supplementary file17 (DOCX 14 KB) [file 122_2021_3838_MOESM17_ESM.docx]

Supplementary Material

**Supplementary Table S1. Primer sequences used in study.**

**Supplementary Table S2. The complete or partial sequences for the 19 RhMLO detected for the 19 *RhMLO*s in the 22 rose accessions.**

**Supplementary Table S3. The number of times sequences were found in different rose samples**

**Supplementary File S1. RoMLO19.rar**

**Supplementary Figure S1. Multiple amino acid sequence alignment of *RhMLO*s.** The alignment was generated by Clustalx1.83 using default parameters. Dark blue boxes indicate identical amino acids and pink or blue boxes indicated similar amino acids. Seven transmembrane domains (TM) are underlined by black bars and the position of the calmodulin-binding site (CaMBD) is indicated by a gray bar. Two conserved domains identified by Panstruga (2005) within the highly polymorphic C-termini are highlighted in numbered boxes (I and II).

**Supplementary Figure S2. Expression profiles of transcripts encoding 19 *RhMLO genes* associated with flower** **development and scent.** The heatmap was generated based on available RPKM data from *ROSAseq* (<https://lipm-browsers.toulouse.inra.fr/plants/R.chinensis>) using the Heml analysis tool. Black boxes indicted that these genes were not found in *ROSAseq.*

**Supplementary Figure S3. Gene structure of the RhMLO1 to 4 with the localization of the cloned fragments.** The black boxes represent the 15 exons of the *RhMLO* genes, the lines represent the intron regions. The red bars above exon no. 15 mark the gene regions that were cloned into the vector p9U10-RNAi. The black bar indicates the scale of 1 kb in length. Figure from Geike et al. (2015), slightly modified after Kaufmann et al. (2012).

**Supplementary Figure S4. Transient silencing of rose MLO genes via RNAi.** Relative expression (RQ-value) of RhMLO1, RhMLO2 and RhMLO3/4 in comparison to control infiltrations with RNAi GUS constructs. Significance * = P <0.05, ** = P<0.001 Due to high similarities between RhMLO3 and RhMLO4 expression was only monitored for RhMLO4. Horizontal lines in boxes represent the median of four independent experiments with three technical replications each. The means of these experiments are also shown.

**Supplementary Excel File S1. Best Blastp hits for the *MLO* genes in different plant species.** We used an e-value of 1e^-5^ as minimum.

**Supplementary Excel File S2. Position of all SNPs and indels in the 19 RhMLO genes.**

**Supplementary PDF S1. Sequence alignment of *RhMLO1***

**Supplementary PDF S2. Sequence alignment of *RhMLO2***

**Supplementary PDF S3. Sequence alignment of *RhMLO3***

**Supplementary PDF S4. Sequence alignment of *RhMLO4***

**Supplementary PDF S5. Sequence alignment of *RhML17***
